# A review of recent advances in electrochemical and photoelectrochemical late-stage functionalization classified by anodic oxidation, cathodic reduction, and paired electrolysis

**DOI:** 10.3762/bjoc.20.214

**Published:** 2024-10-09

**Authors:** Nian Li, Ruzal Sitdikov, Ajit Prabhakar Kale, Joost Steverlynck, Bo Li, Magnus Rueping

**Affiliations:** 1 KAUST Catalysis Center (KCC), King Abdullah University Science and Technology (KAUST), Thuwal 23955-6900, Saudi Arabia; 2 Institute for Experimental Molecular Imaging, RWTH Aachen University, Forckenbeckstrasse 55, 52074, Aachen, Germany

**Keywords:** electrochemistry, late-stage functionalization, paired electrolysis, pharmaceutical drugs, photoelectrochemistry

## Abstract

With the resurgence of electrosynthesis in organic chemistry, there is a significant increase in the number of routes available for late-stage functionalization (LSF) of drugs. Electrosynthetic methods, which obviate the need for hazardous chemical oxidants or reductants, offer unprecedented control of reactions through the continuous variation of the applied potential and the possibility of combination with photochemical processes. This capability is a substantial advantage for performing electrochemical or photoelectrochemical LSF. Ultimately, these protocols are poised to become a vital component of the medicinal chemist's toolkit. In this review, we discuss electrochemical protocols that have been demonstrated to be applicable for the LSF of pharmaceutical drugs, their derivatives, and natural substrates. We present and analyze representative examples to illustrate the potential of electrochemistry or photoelectrochemistry for the LSF of valuable molecular scaffolds.

## Introduction

Organic electrochemistry is gaining increasing interest in both academia and industry due to its numerous advantages and potential applications [1–2]. Electrochemical methods can reduce costs and waste generation by eliminating the need for chemical oxidants or reductants, and they can be safely and easily scaled up in flow reactors for industrial applications. The potential for upscaling and applicability towards LSF makes electrosynthesis particularly appealing for the fine-chemical and pharmaceutical industry. As the electrode potential can be fine-tuned over a continuous scale, higher functional group compatibility can be achieved compared to many classical methods. In light of the general trend towards more chemoselective protocols with broader functional group compatibility, there has been a growing interest in exploring the potential of electrosynthesis for the late-stage functionalization of complex scaffolds. Additionally, the increased interest of medicinal chemists in electrochemical methods, combined with their continuous search for new LSF strategies, will further extend the applicability of newly established electrochemical methods.

LSF is a rapidly growing field that offers new opportunities in drug discovery [3]. Transition-metal-catalyzed LSF strategies have been well-established over the past decades. More recently, with the vigorous development of photochemistry and electrochemistry, numerous innovative reports on LSF using photo-, electro-, and photoelectrochemistry have emerged. These areas have been systematically summarized, classifying them by targeted C–H bond functionalization and the newly formed bonds [4–5].

**Figure 1:**
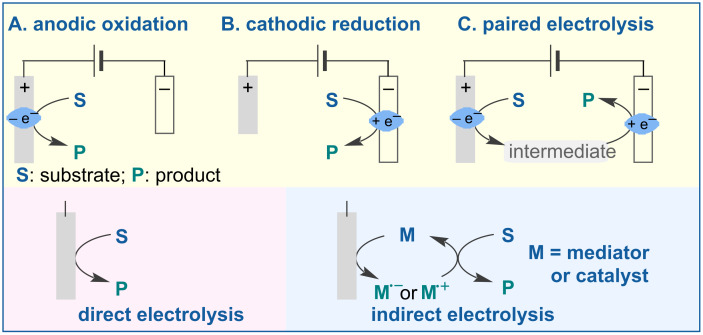
Classification of LSF reactions in this review.

In this review, we aim to provide a comprehensive classification and overview of the currently available electrochemical and photoelectrochemical methods for the LSF of pharmaceutical drugs and natural products. We classify these advancements into three types: anodic oxidation, cathodic reduction, and paired electrolysis (Figure 1). This review considers direct electrolysis (oxidation or reduction), mediator-induced electrolysis, and metal-catalyzed and photocatalyzed electrochemical transformations. Detailed reaction conditions, such as electrolyte, electrode material, and the use of constant current or constant voltage, are presented. Additionally, we discuss the mechanisms of some representative reactions and provide selected examples of LSF of relevant bioactive compounds.

## Review

### 1 LSF via anodic oxidation

To date, the majority of electrosynthetic methods in organic chemistry consists of anodic oxidations. These techniques are generally more robust and can often be performed outside of a glovebox, making them particularly attractive for larger scale applications in industrial settings. An anodic oxidation is frequently employed for C–H functionalization, which can simplify late-stage functionalization strategies. Additionally, many of these synthetic methods do not require precious metals, enhancing their appeal in terms of sustainability and cost-effectiveness. However, it should be noted that anodic oxidations often require electrodes with high resistance to oxidation, such as platinum electrodes, or inert electrodes with a highly developed surface, like reticulated vitreous carbon (RVC). Anodic oxidations generally involve the evolution of hydrogen (indicated in schemes as H_2_↑) in the cathodic half-reaction, which will however not be addressed in greater detail in this review.

#### 1.1 Direct anodic oxidation of substrates

**Scheme 1:**
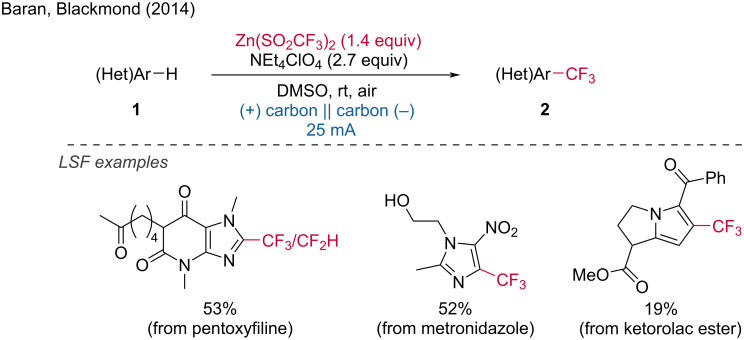
C(sp^2^)–H trifluoromethylation of heteroarenes.

**1.1.1 C–H bond functionalization. *C–H bond carbofunctionalization:*** CF_3_ groups can be installed on heteroarenes at a late stage via a TM-free electrochemical method. This route was reported in 2014 by the Baran and Blackmond groups [6]. A commercially available reagent, Zn(SO_2_CF_3_)_2_, was used as the CF_3_ radical source in the reaction. Additionally, a series of substrates could be difluoromethylated under the reported electrochemical conditions. A comparison was made between the developed electrochemical conditions for each substrate and an analogous non-electrochemical method using peroxide for CF_3_ radical generation. In all cases, the electrochemical route delivered improved yields (Scheme 1).

**Scheme 2:**
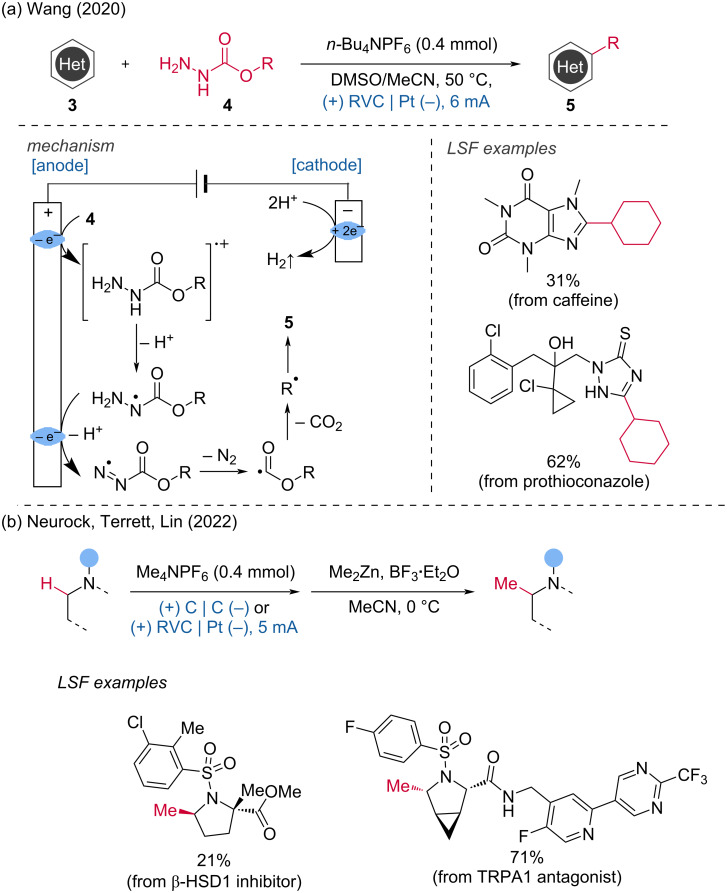
C(sp^2^)–H and C(sp^3^)–H alkylation of complex molecules.

The Wang group later discovered a C(sp^2^)–H functionalization method where primary, secondary, and tertiary alkyl radicals can be readily generated through the sequential anodic oxidative fragmentation of alkyl carbazates, enabling the functionalization of N-heteroarenes [7]. This transformation is particularly valuable as the cleavage of the C–O bond to activate alcohols presents a significant synthetic challenge. The carbazate substrates are easily prepared from ubiquitous alcohol precursors. The first stage of the transformation involves the sequential anodic oxidation of the carbazate and subsequent deprotonation to form a diazenecarboxylate. Further anodic oxidation cleaves the diazene, resulting in the formation of an acyl radical and the release of molecular nitrogen. The subsequent step involves the decarboxylation of the acyl radical to produce an alkyl radical. This method was successfully applied to the late-stage functionalization of bioactive compounds such as caffeine and prothioconazole (Scheme 2a). Additionally, Lin, Terrett and Neurock's group [8] reported the electrochemical C(sp^3^)–H methylation of complex molecules. This strategy enabled the synthesis of the "magic methyl" product, a TRPA1 antagonist (Scheme 2b).

**Scheme 3:**
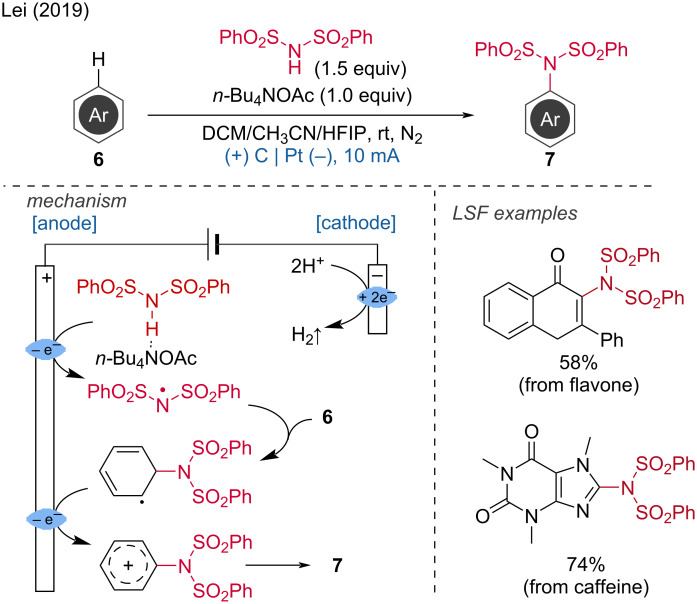
Electrochemical oxidation-induced intermolecular aromatic C–H sulfonamidation.

***C–H bond amination*:** Direct and selective CH-aminations and amidations are challenging reactions. In this context, the regioselective sulfonamidation of (hetero)aromatic groups was achieved by the Lei group via dehydrogenative aryl C–H/N–H cross-coupling [9]. A crucial step in this transformation is the generation of sulfamidyl radicals via a concerted proton-coupled electron transfer (PCET). This process occurs after the formation of a hydrogen bond between dibenzenesulfonimide and *n*-Bu_4_NOAc. The formed sulfamidyl radical can directly react with the (hetero)aromatic ring. Subsequent anodic oxidation produces a carbocation intermediate, which rearomatizes through proton loss. Concurrently, the cathodic reduction of the generated protons produces H_2_. In addition to (hetero)aromatic groups, alkene scaffolds also underwent this reaction (Scheme 3).

**Scheme 4:**
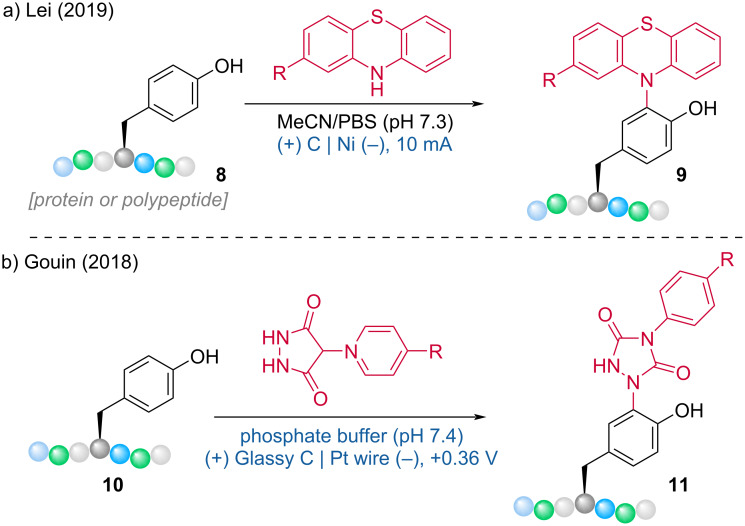
Bioconjugation of tyrosine with (a) phenothiazine and (b) urazole derivatives.

In the same year, the Lei group [10] extended the electrochemical C(sp^2^)–H functionalization C–N coupling reaction by developing an electrochemical method for the bioconjugation of tyrosine in proteins/polypeptides with phenothiazine residues, achieving excellent site- and chemoselectivity (Scheme 4a). This method was inspired by an earlier work from the Gouin group, which reported the merger of electrochemistry and bioconjugation in 2018 (Scheme 4b) [11].

**Scheme 5:**
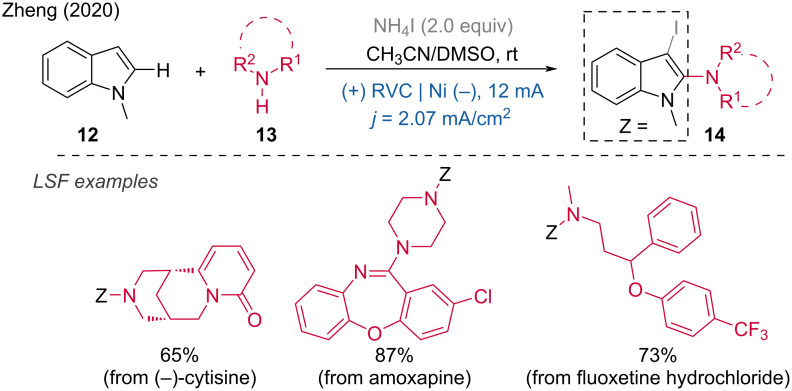
Electrochemical iodoamination of indoles using unactivated amines.

In 2020, Zheng and coworkers developed an interesting iodoamination of indoles using unactivated amines and benzotriazoles [12]. This difunctionalization reaction was carried out in an undivided cell with an RVC anode and a foamed Ni cathode, at a constant current of 12 mA in DMSO at room temperature under atmospheric conditions. The reaction has been applied to more than 80 examples, including the late-stage functionalization of natural products and pharmaceuticals, as well as the synthesis and radiosynthesis of ¹³¹I-labeled compounds. For example, the late-stage iodoamination of cytisine, amoxapine, and fluoxetine hydrochloride was achieved with yields of 65%, 87%, and 73%, respectively. Additionally, this transformation was successful for gram-scale synthesis via batch and flow chemistry, indicating significant potential for further industrial and medicinal chemistry applications (Scheme 5).

**Scheme 6:**
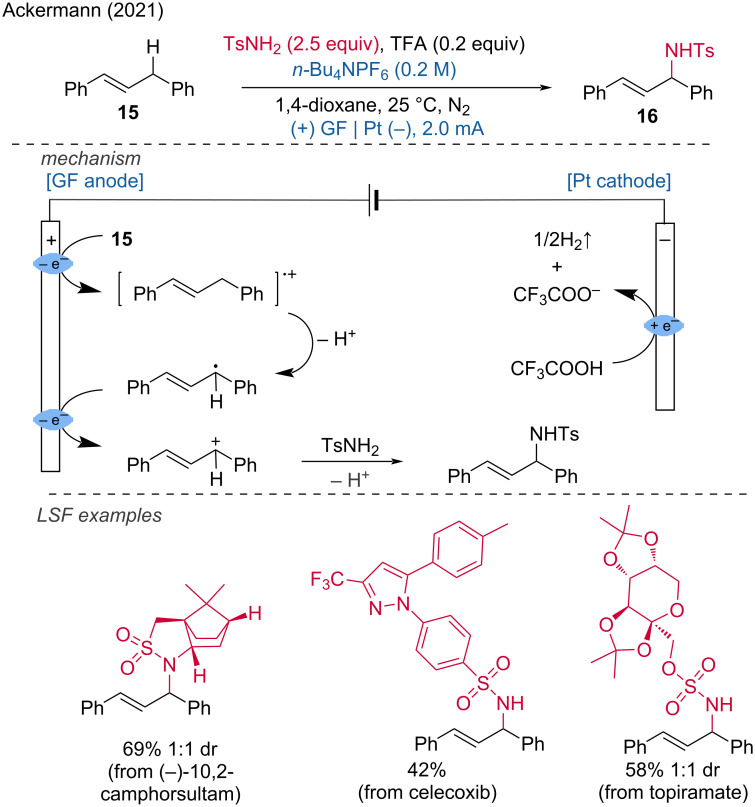
Allylic C(sp^3^)–H aminations with sulfonamides.

Furthermore, Ackermann and coworkers described a straightforward C(sp^3^)–H amination of 1,3-diarylpropenes with sulfonamides via direct oxidation of allylic C(sp^3^)–H bonds [13]. During the reaction process, a radical cation is formed by oxidation of the substrate at the anode. This radical cation is subsequently deprotonated to produce an allyl radical. The allyl radical is further oxidized to form the allyl cation, which is then attacked by the nucleophilic sulfonamide, leading to the formation of the desired C–N-bond product. To demonstrate the mildness of the LSF reaction conditions, celecoxib and topiramate sulfonamides were easily functionalized with 1,3-diarylpropene in moderate yields (Scheme 6).

**Scheme 7:**
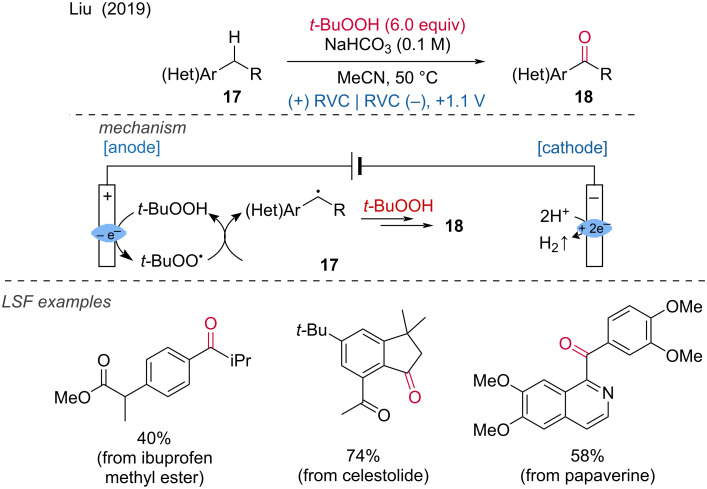
Electrochemical benzylic oxidation of C–H bonds.

***C–H bond oxygenation:*** In addition to electrochemical C–H aminations, C–H oxygenations have also been reported. For example, Liu and colleagues demonstrated the electrochemical oxidation of benzylic C–H bonds to ketones using *tert*-butyl hydroperoxide as the radical initiator [14]. This method was applied to functionalize bioactive molecules, with celestolide, ibuprofen methyl ester, and papaverine being oxidized at the benzylic position in good yields. A gram-scale test was conducted to confirm the potential for large-scale applications. According to the authors, the electrochemical oxidation of *t*-BuOOH at the anode leads to a *tert*-butyl peroxyl radical that activates the C–H bond at the benzylic position of the substrate. The formed radical reacts with *t*-BuOOH to produce the corresponding ketone, with *tert-*butanol as a byproduct (Scheme 7).

**Scheme 8:**
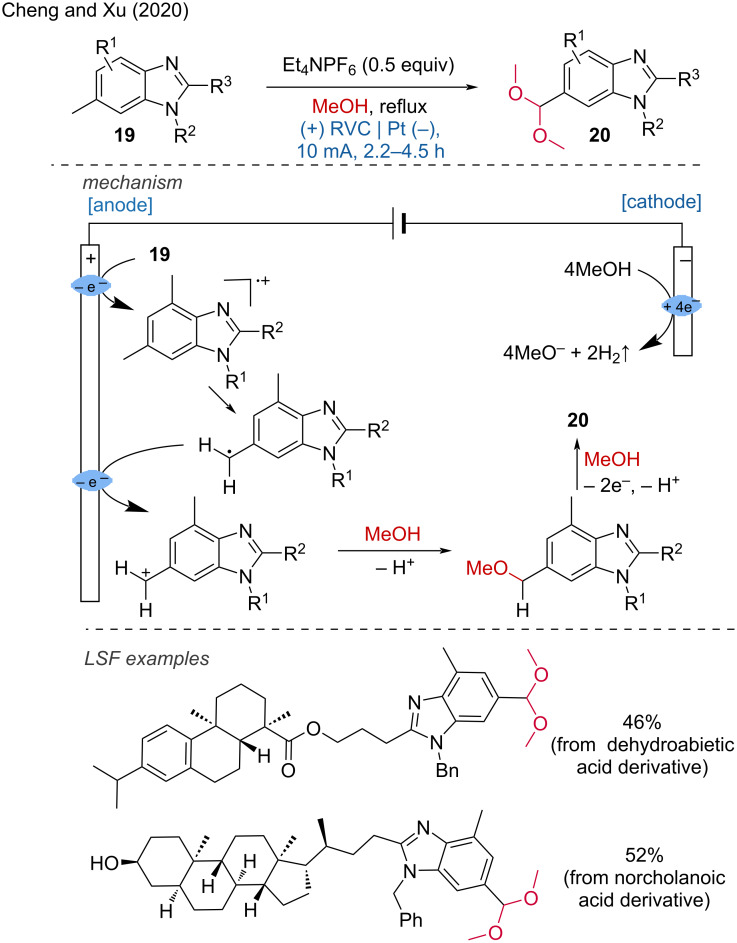
Site-selective electrooxidation of methylarenes to aromatic acetals.

A closely related transformation was developed by Cheng and Xu in 2020 for the electrooxidation of methylarenes to aromatic acetals [15]. With this method, several structurally diverse aromatic acetals have been synthesized. Dehydroabietic and norcholanoic acid derivatives have been effectively modified using the developed protocol. The reaction is reported to involve the oxidation of the benzene core, followed by electron transfer to the radical cation, and subsequent C–H abstraction. The methylarene undergoes oxidation, deprotonation, and a second oxidation before being captured by MeOH to produce a monomethoxylated product. This intermediate then undergoes a second oxidation round to yield the final product. Additionally, the same group disclosed an aromatic C–H hydroxylation process by combining continuous flow chemistry and electrochemistry (Scheme 8) [16].

**Scheme 9:**
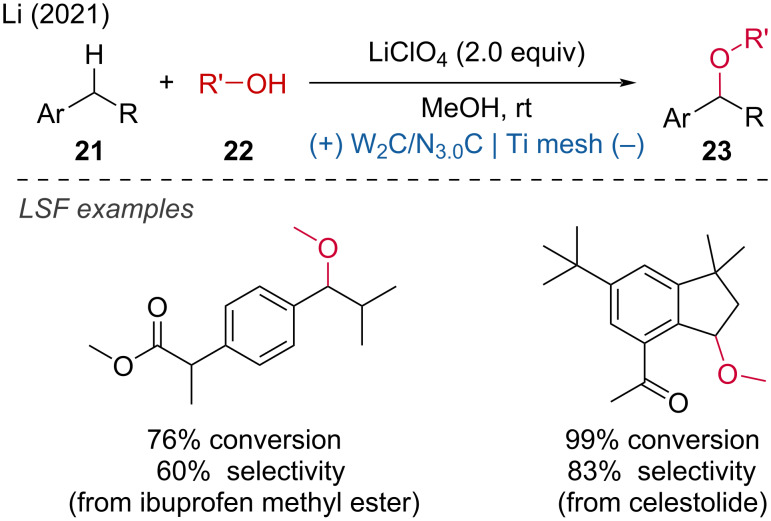
Electrochemical activation of C–H by electron-deficient W_2_C nanocrystals.

The surface modification of electrodes can lead to improved reactivity and selectivity. In this regard, Li and coworkers developed electron-deficient W_2_C nanocrystal-based electrodes to enhance the direct activation of C(sp^3^)–H bonds under mild conditions [17]. The pronounced electron-deficient W_2_C nanocatalysts greatly facilitate the direct deprotonation process, ensuring the longevity of the electrode by overcoming self-oxidation. The LSF of drug molecules such as ibuprofen methyl ester and celestolide showed high yields and good selectivity (Scheme 9).

**Scheme 10:**
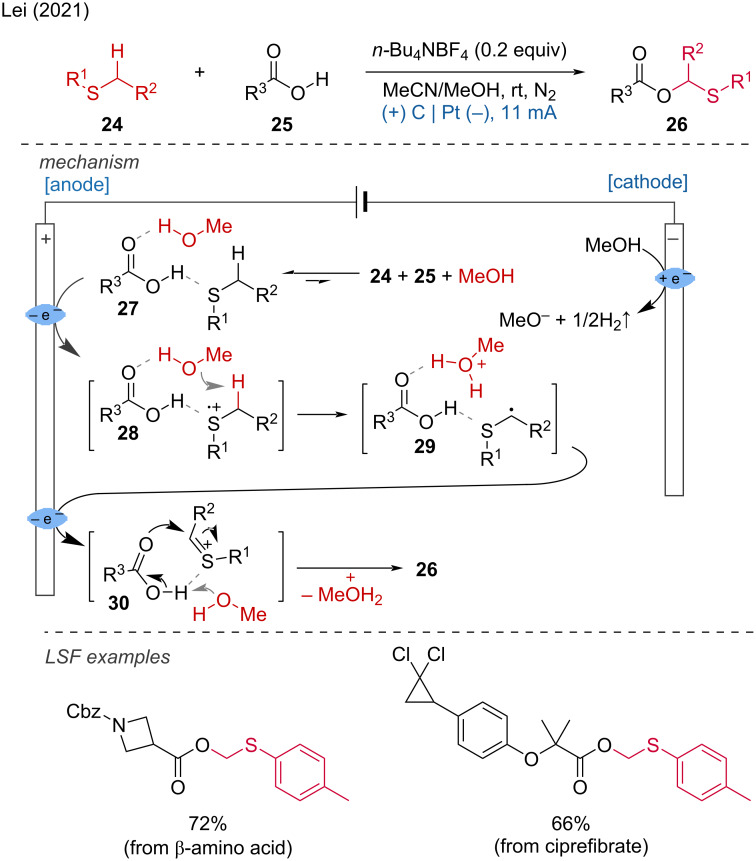
α-Acyloxy sulfide preparation via C–H/OH cross-dehydrogenative coupling.

The Lei group also disclosed another C(sp^3^)–H functionalization involving C–O-bond formation [18]. The reported method allows the straightforward preparation of α-acyloxy sulfides from ubiquitous carboxylic acids and sulfides, providing an alternative to the harsh Pummerer rearrangement. Methanol played a crucial role in achieving the desired transformation and it was suggested to promote the self-assembly of reagents **24** and **25** for the formation of **27**, which allows the selective abstraction of H^+^ from the less sterically hindered side. Subsequently, the generated intermediate **29** is oxidized at the anode, then attacked by the acid to obtain the final product (Scheme 10).

**Scheme 11:**
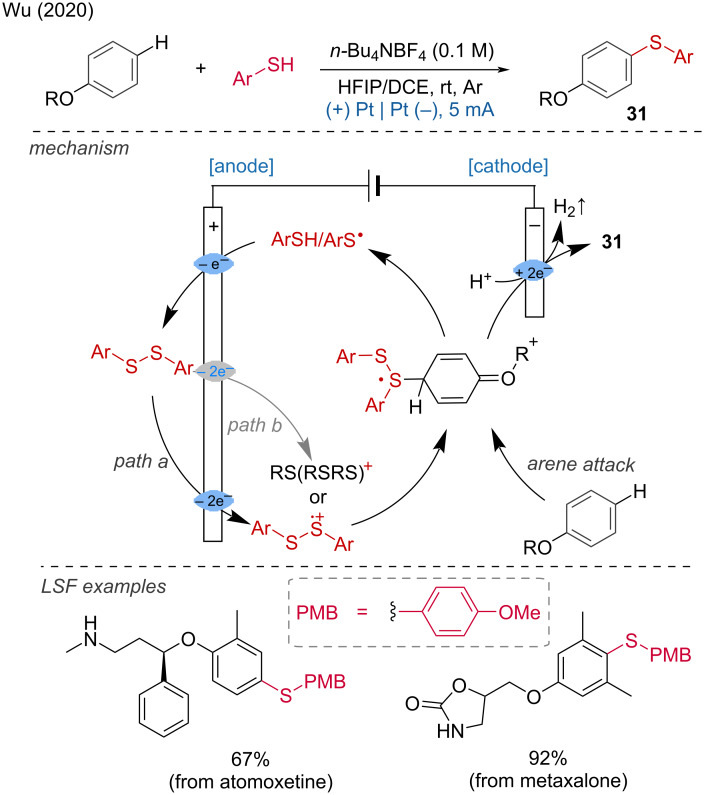
Aromatic C–H-bond thiolation.

***C–H bond sulfur functionalization:*** The direct formation of the CS bond is an attractive way to prepare aryl sulfides. From this perspective, Wu and coworkers developed a method for the regioselective thiolation of aromatic C–H bonds by activating the thiol rather than the arene [19]. For their developed reaction, Pt electrodes were used in an undivided cell with a mixture of HFIP/DCE 3:1 at room temperature under argon. Late-stage functionalization was demonstrated for atomoxetine, metaxalone, and tadalafil. Mechanistically, thiophenol is oxidized at the anode to the corresponding radical by SET, then dimerizes into a disulfide, which is further oxidized into an intermediate cation radical, yielding a highly electrophilic species. Subsequently, a selective anisole attack leads to an intermediate product, which is then deprotonated, generating the thiol radical. This allowed for the preparation of the *para*-thiolation product (Scheme 11).

**Scheme 12:**
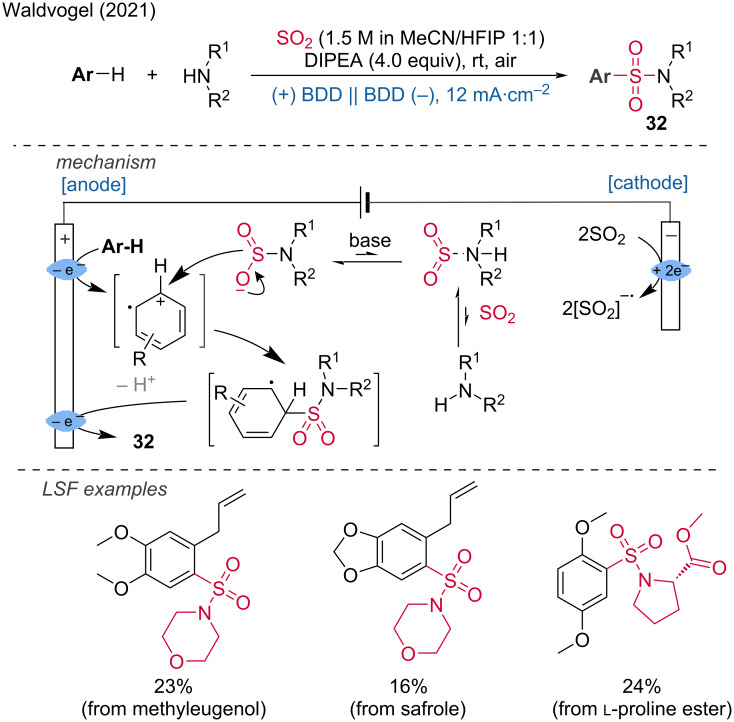
C(sp^2^)–H functionalization for the installation of sulfonamide groups.

Sulfonamides are an important class of bioactive molecules. In 2021, the Waldvogel group disclosed the first C(sp^2^)–H functionalization protocol for the installation of sulfonamide groups using commercially available SO_2_ and amines (Scheme 12) [20]. This method is highly appealing for industrial applications and LSF. The proposed mechanism begins with the anodic oxidation of the arene substrate. The resulting radical cation intermediate is then attacked by the nucleophilic amidosulfinate, which also functions as an electrolyte. The amidosulfinate is generated through the formation of a Lewis acid–base adduct. A subsequent oxidation step, accompanied by deprotonation, yields the sulfonamide product. SO_2_ captures the excess electrons via cathodic reduction and to prevent the reoxidation of the reduced SO_2_ at the anode, a divided cell setup is required (Scheme 12).

**Scheme 13:**
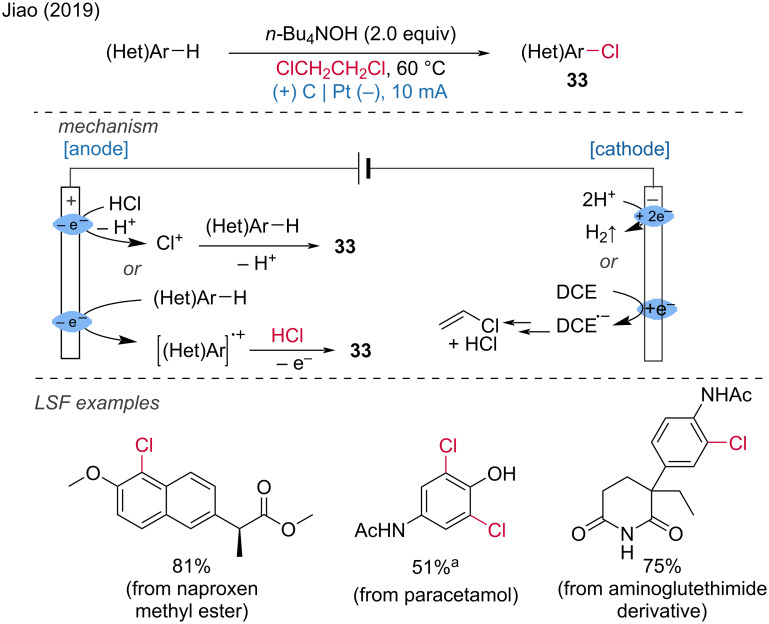
Preparation of (hetero)aryl chlorides and vinyl chloride with 1,2-dichloroethane. ^a^Cu(OAc)_2_ (0.05 equiv) is added.

***C–H bond halogenation:*** Aryl and alkyl halides are important synthetic building blocks for cross-coupling reactions as well as bioactive molecules with applications in agrochemical and pharmaceutical chemistry. In 2019, Jiao and colleagues reported that 1,2-dichloroethane (DCE) could be used as a chlorination reagent for the production of (hetero)aryl chlorides and vinyl chlorides [21]. The reactions were carried out in an undivided cell containing a mixture of DCE in methanol, equipped with a graphite anode and a platinum plate cathode, under a current of 10 mA at 60 °C for 3–20 hours. Several electrochemical LSF of pharmacologically active molecules were tested, including naproxen methyl ester, a derivative of aminoglutethimide, and paracetamol. The corresponding products were obtained in good yields (51–81%). The reaction involves the catalytic dehydrochlorination of DCE at the cathode, simultaneously with anodic oxidative aromatic chlorination using cathodically released HCl as the chloride source (Scheme 13).

Additionally, the Lei group demonstrated a double oxidation strategy to obtain α-chlorosulfoxides from sulfides using hydrochloric acid as a bifunctional reagent [22]. This strategy accommodates a broad range of substrates and offers high diastereoselectivity and regioselectivity.

**Scheme 14:**
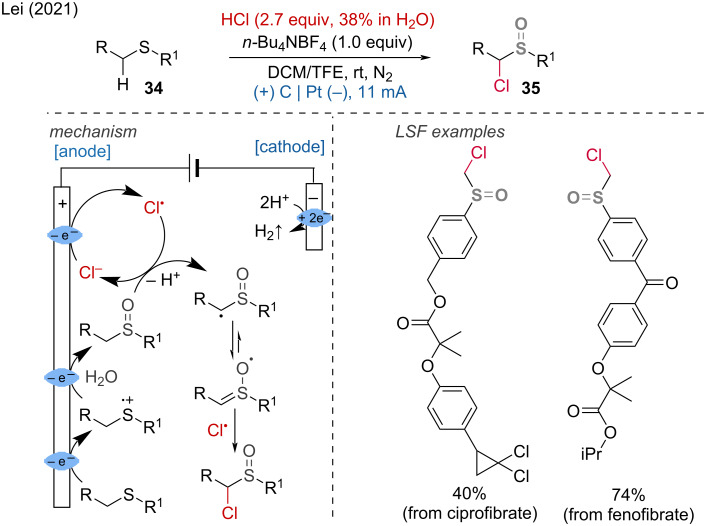
Electrochemical dual-oxidation enables access to α-chlorosulfoxides.

Several LSF modifications of amino acids and pharmaceutical derivatives further emphasized its utility. Mechanistic studies have demonstrated that the key to this selective chemical conversion lies in the dual oxidation process at the anode. The authors suggest that anodic oxidation of the sulfide generates a sulfur radical cation intermediate, which reacts with water at the anode to form a sulfoxide. Subsequent hydrogen atom abstraction by a chlorine radical leads to the formation of an intermediate carbon radical, whose resonant intermediate reacts with another chlorine radical to produce the desired α-chlorosulfoxide product (Scheme 14).

**Scheme 15:**
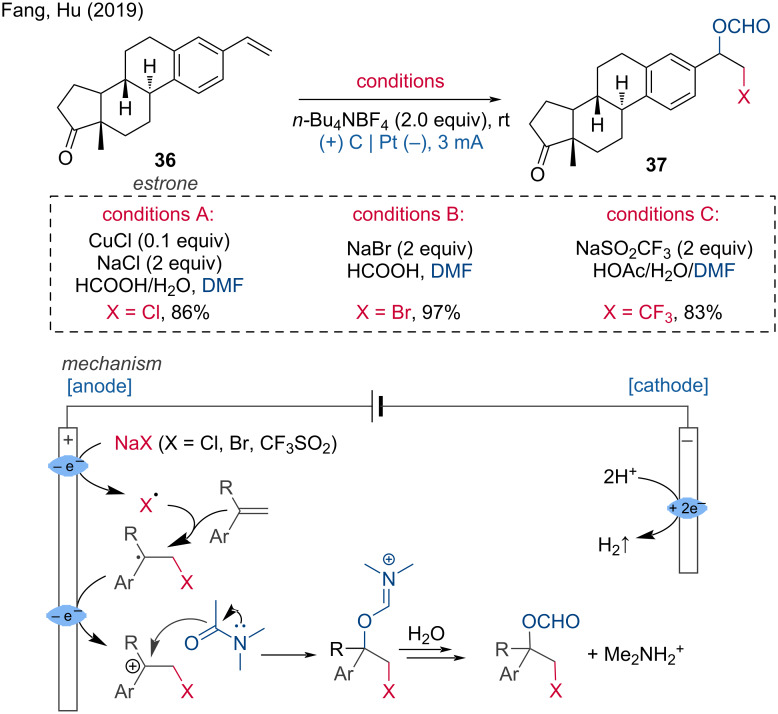
Regio- and chemoselective formyloxylation–bromination/chlorination/trifluoromethylation of alkenes.

**1.1.2 Unsaturated bond functionalization.** Difunctionalizations of double and triple bonds are of high interest as they allow the introduction of two functional groups in a single step. An interesting electrochemical difunctionalization of styrene and cyclic olefin derivatives has been reported by the Hu group [23]. They combined oxyformylation with brominations/chlorinations/trifluoromethylations using DMF and NaBr/NaCl/NaSO_2_CF_3_ as readily available reagents. The reported yields for this regio- and chemoselective transformation are high. For each reaction type, one LSF example was demonstrated using an estrone derivative. Mechanistically, this transformation can be understood as follows: first, a Br/Cl/CF_3_ radical is formed via anodic oxidation, which subsequently attacks the olefin. The newly formed benzyl radical is oxidized to a carbocation, which undergoes nucleophilic attack by DMF. Hydrolysis of the imine delivers the final product (Scheme 15).

**Scheme 16:**
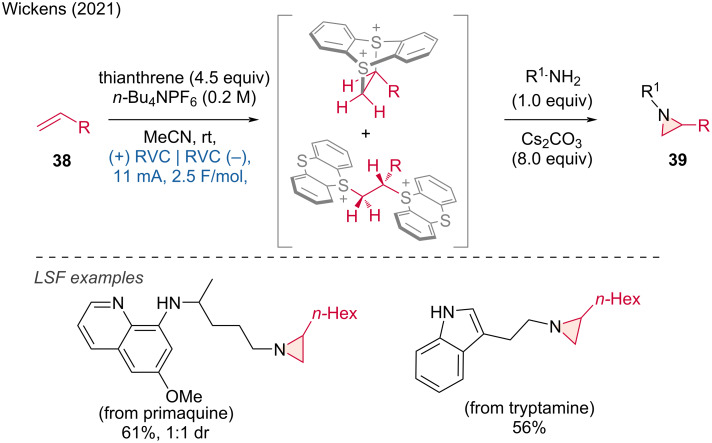
Aziridine formation by coupling amines and alkenes.

The synthesis of aziridines can be achieved via the formation of nitrenes in either a metal-catalyzed or metal-free fashion. In this context, Wickens and colleagues presented a remarkable dication pool strategy for accessing *N*-alkylaziridines via metastable dicationic intermediates derived from the interaction of non-activated alkenes with thianthrene [24]. This procedure has the advantage of separating the oxidative activation of the alkenes from the aziridination step, allowing efficient access to a variety of aziridine building blocks containing sensitive functional groups. This was demonstrated by the LSF of primary natural and pharmaceutical amines carrying potential competing nucleophiles, such as tryptamine and primaquine (Scheme 16).

**Scheme 17:**
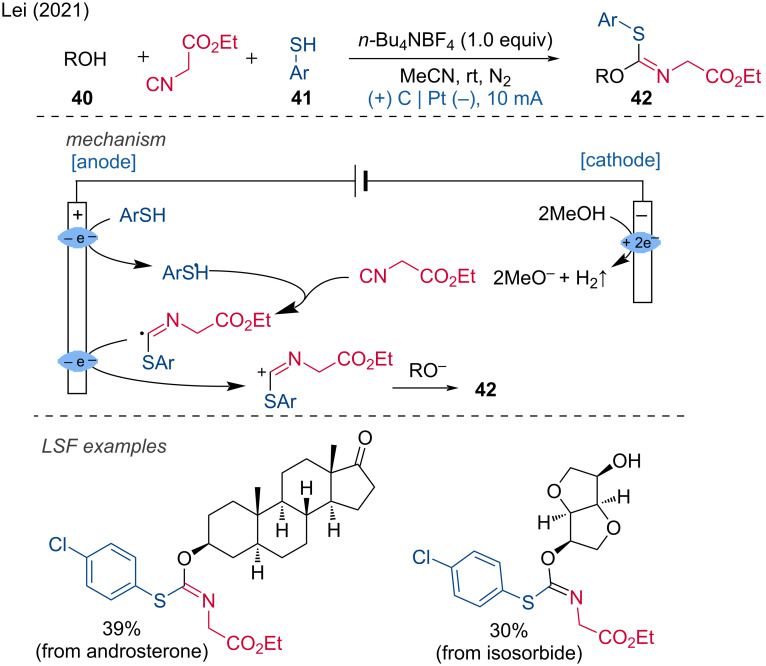
Formation of iminosulfide ethers via difunctionalization of an isocyanide.

In the context of electrochemical difunctionalizations the Lei group published a transition-metal-free electrochemical difunctionalization method for the formation of C–S bonds [25]. This method achieves the difunctionalization of a carbon atom by reacting isocyanide 2-isocyanoacetate with a thiophenol and an alcohol. The scope is very broad for thiophenols, alcohols, and isocyanides, and even alkyl thiols are compatible. In addition to aliphatic alcohols, benzyl alcohols are also suitable reagents. Numerous LSF examples and upscaling were demonstrated. The mechanism involves two anodic oxidations: first, the thiophenol is oxidized at the anode, forming a sulfur radical that attacks the isocyanide. The newly formed carbon radical is then oxidized to a carbocation, which is subsequently attacked by the alkoxide to furnish the final product (Scheme 17).

**Scheme 18:**
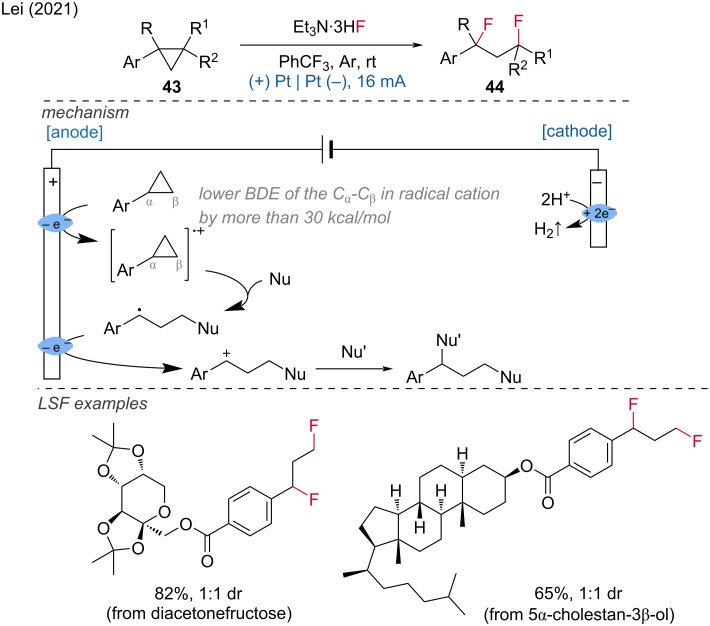
Synthesis of 1,3-difunctionalized molecules via C–C-bond cleavage of arylcyclopropane.

The Lei group also demonstrated C–F-bond formations, particularly developing an electrochemical method for the cleavage of C–C bonds and the 1,3-difunctionalization of arylcyclopropanes [26]. This electrochemical approach provides a convenient strategy for constructing 1,3-difluorinated molecules by employing Et_3_N·3HF as a nucleophilic fluorine source. Due to the mild reaction conditions, the LSF was demonstrated for complex natural precursors such as 5α-cholestan-3β-ol and androsterone scaffolds. During the reaction, the arylcyclopropane is oxidized at the anode to form a radical cation, causing the weakening of the Cα–Cβ bond. The radical cation then undergoes a three-electron S_N_2 reaction to generate a benzylic radical, which loses an electron at the anode to form a benzylic carbocation. Nucleophilic attack on the benzylic carbocation results in a 1,3-difunctionalized product (Scheme 18).

**Scheme 19:**
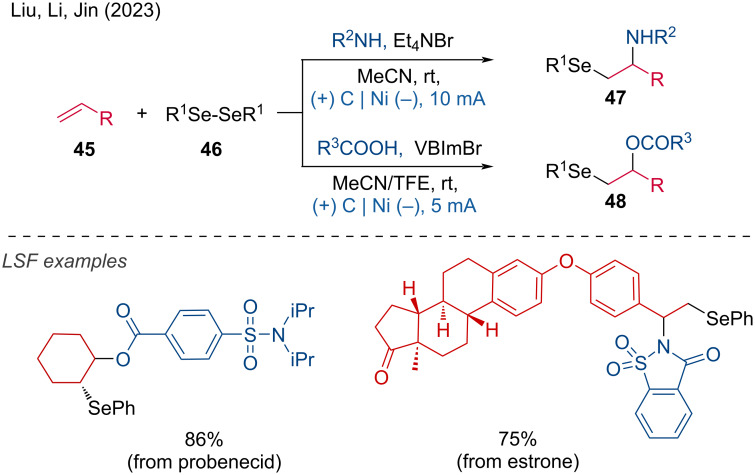
Electrooxidative amino- and oxyselenation of alkenes. VBImBr = 1-butyl-3-vinylimidazolium bromide.

The introduction of two heteroatoms was reported by Liu, Li, and Jin [27]. They developed a method demonstrating excellent tolerance for a wide range of readily available alkenes and *O*,*N*-centered nucleophiles, showcasing 118 examples with good to high yields. The LSF of complex molecules, such as probenecid and estrone, highlighted the potential application of this method in the synthesis of selenium-containing drugs (Scheme 19).

**Scheme 20:**
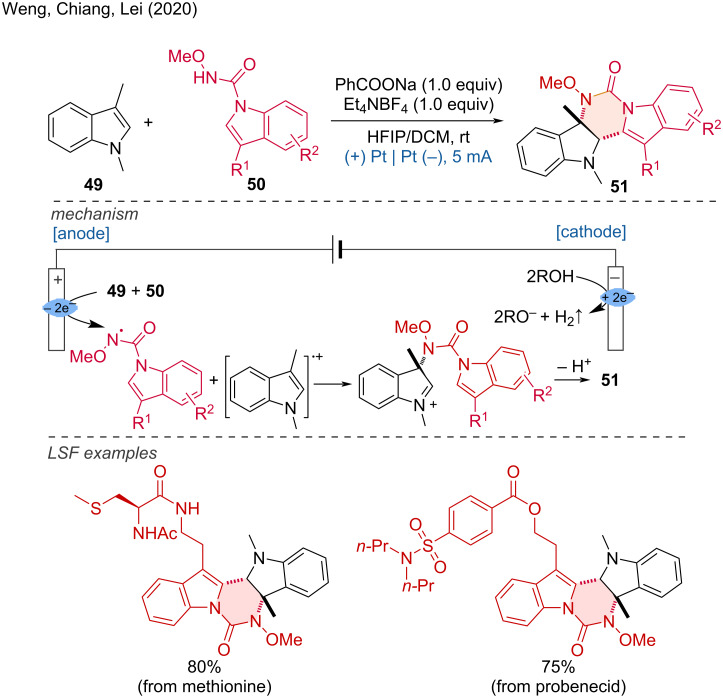
Electrooxidative dehydrogenative [4 + 2] annulation of indole derivatives.

**1.1.3 Annulation.** Annulation refers to the formation of rings, a process that involves building a ring onto a preexisting system, whether cyclic or non-cyclic. The Lei group developed an electrooxidative annulation reaction that facilitates LSF [28]. A regio- and stereoselective protocol was established for the [4 + 2] annulation of indole derivatives, allowing access to highly functionalized pyrimido[5,4-*b*]indoles due to its high functional group tolerance. Multiple examples were demonstrated with indole 1*H*-carboxamides linked to drug molecules or natural products at the R^2^ position. Additionally, an alkyl azide at the R^2^ position and an iodide at the R^1^ position were tolerated, enabling further functionalization. The proposed mechanism involves radical–radical cross-coupling. The indole 1*H*-carboxamide generates a nitrogen-centered radical during anodic oxidation in the presence of a base, while the 1,3-dimethylindole derivative forms an indole radical cation. The radical–radical cross-coupling between these two intermediates, followed by intramolecular cyclization and subsequent deprotonation results in the desired product (Scheme 20).

**Scheme 21:**
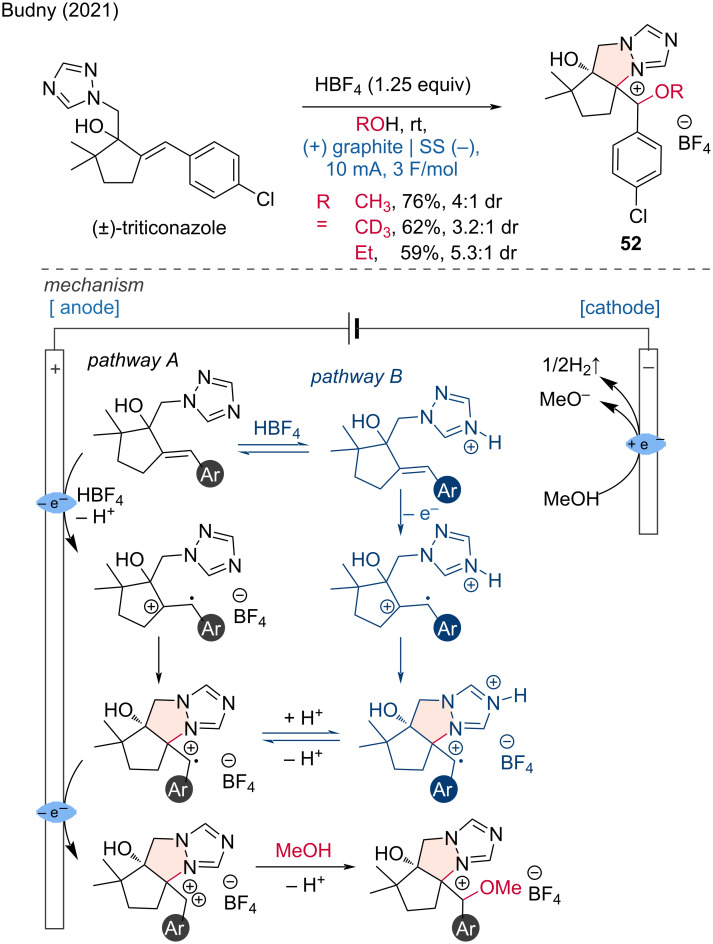
Electrochemical cyclization combined with alkoxylation of triticonazole.

Furthermore, Budny and coworkers demonstrated that (±)-triticonazole and related compounds could be cyclized and alkoxylated to the corresponding 1,2,4-triazolium tetrafluoroborates under electrochemical conditions [29]. The reaction is conducted with a stoichiometric amount of HBF_4_, which converts the substrate to the corresponding cationic intermediate via a protonation, eliminating the need for an additional supporting electrolyte. The proposed mechanism involves the one-electron oxidation of triticonazole to form a radical cation, followed by cyclization to an intermediate. Subsequent anodic oxidation forms a doubly charged cation, which is then captured by methanol and deprotonated to yield the final product (pathway A). Additionally, due to the protonation of triticonazole, the participation of the protonated form in the overall reaction mechanism is also considered in pathway B (Scheme 21).

**Scheme 22:**
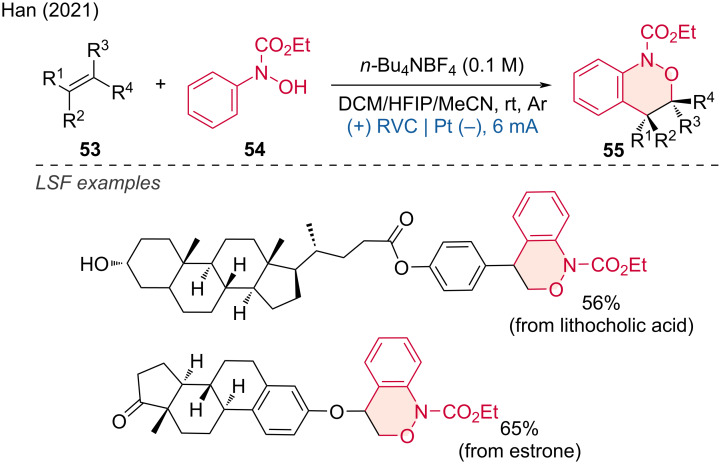
Electrochemically tuned oxidative [4 + 2] annulation of olefins with hydroxamic acids.

Benzo[*c*][1,2]oxazines are useful scaffolds for the synthesis of natural products. In 2021, the Han group developed the electrochemical [4 + 2] annulation of hydroxamic acids **54** with alkenes for approaching benzo[*c*][1,2]oxazines [30]. This method successfully achieved the LSF of several natural products such as lithocholic acid and estrone, affording the following benzo[*c*][1,2]oxazine derivatives in moderate to good yields (Scheme 22).

**Scheme 23:**
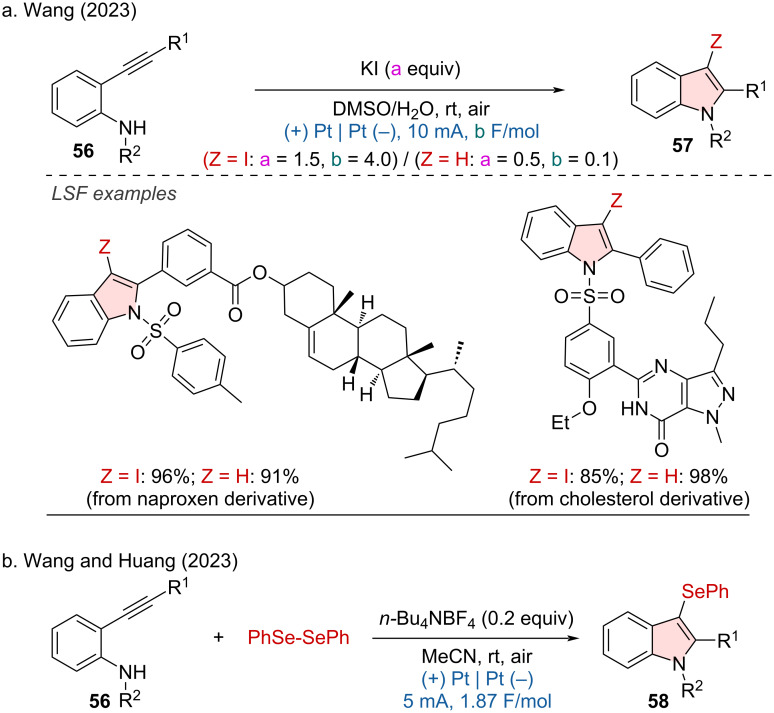
Electrosynthesis of indole derivatives via cyclization of 2-ethynylanilines.

The cyclization of 2-ethynylanilines has been proven to be one of the most effective strategies for synthesizing indole derivatives. In this regard, Wang and coworkers developed the electrosynthesis of 3-iodoindoles from 2-ethynylanilines under mild and straightforward conditions [31]. The functionalization of complex molecules, such as naproxen and cholesterol derivatives, demonstrated good functional group compatibility (Scheme 23a). In the same year, Wang and Huang group reported a similar approach using electrochemical methods to synthesize 3-selenylindoles via the cyclization of 2-ethynylanilines and diselenides (Scheme 23b) [32].

#### 1.2 Organo-mediators-enabled anodic oxidation

**Scheme 24:**
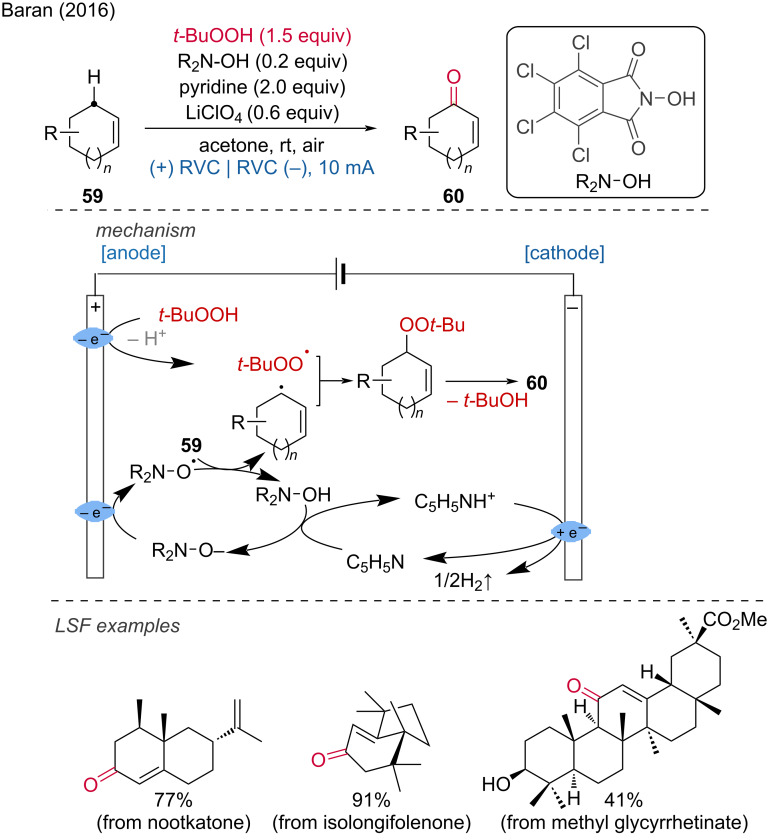
Allylic C–H oxidation of mono-, di-, and sesquiterpenes.

The Baran group has made significant contributions to the field of organic electrochemistry. One of their developments is the electrochemical allylic oxidation, a highly useful C–H functionalization method applicable to several natural products such as mono-, di-, tri-, and sesquiterpenes, along with some steroids [33]. Crucial to their method was the use of a phthalimide-based mediator, adopted from earlier works (1968–1985). RVC electrodes with a highly developed surface area were employed for the reaction. The proposed mechanism is as follows: pyridine deprotonates tetrachloro-*N-*hydroxyphthalimide (R_2_N–OH), which is subsequently anodically oxidized. The resulting *N-*oxyl radical abstracts a hydrogen atom from the position adjacent to the olefin, forming an allylic radical. This allylic radical then reacts with cathodically generated *tert*-butyl peroxide to form an allylic peroxide, which ultimately transforms into an enone upon elimination of *t*-BuOH (Scheme 24).

**Scheme 25:**
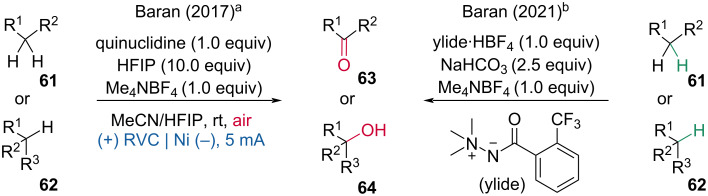
Oxidation of unactivated C–H bonds.

One year later, they developed an electrochemical transformation closely related to their electrochemical allylic oxidation, i.e. the oxidation of unactivated C(sp^3^)–H bonds (Scheme 25a) [34]. Besides, the same group published a comprehensive analysis on *N-*ammonium ylide mediators, which were found to be superior to quinuclidine scaffolds for a chemoselective C(sp^3^)–H oxidation (Scheme 25b) [35].

**Scheme 26:**
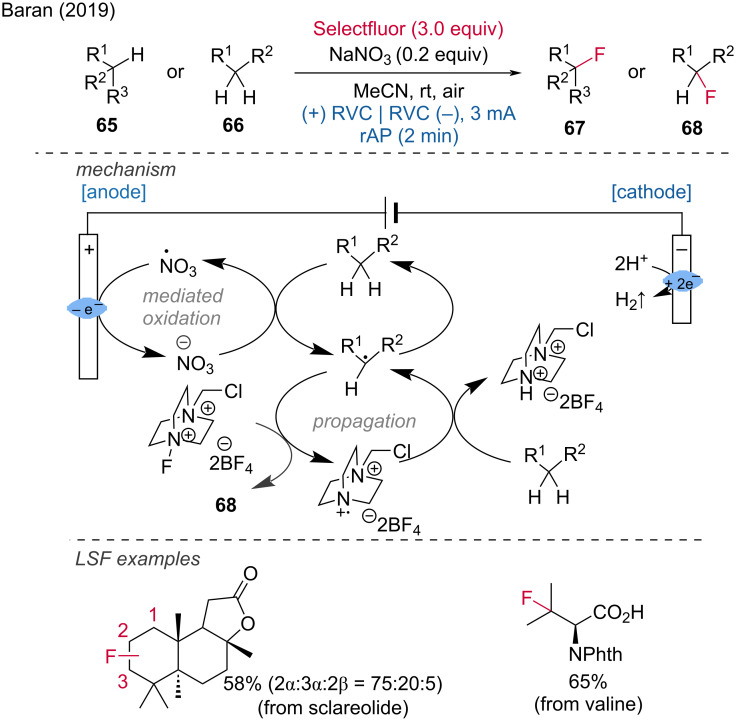
Fluorination of C(sp^3^)–H bonds. rAP = rapid alternating polarity.

The electrochemical C(sp^3^)–H fluorination of unactivated C–H bonds is another important transformation via anodic oxidation realized by the Baran group [36]. The choice of Selectfluor, which plays multiple roles, was crucial. In addition to functioning as a fluorine source, Selectfluor also acts as a mediator similar to quinuclidine and serves as an electrolyte. The method was demonstrated to be scalable for natural products such as sclareolide and protected ʟ-valine. The proposed mechanism involves a radical chain process. Initiation occurs by nitrate-mediated or direct electrochemical anodic oxidation, followed by fluorination with Selectfluor. After fluorination, the Selectfluor reagent abstracts a hydrogen atom from the substrate, which can then undergo further fluorination. The nitrate additive proved helpful as an initiator but is not necessary for certain substrates like sclareolide and protected ʟ-valine (Scheme 26).

**Scheme 27:**
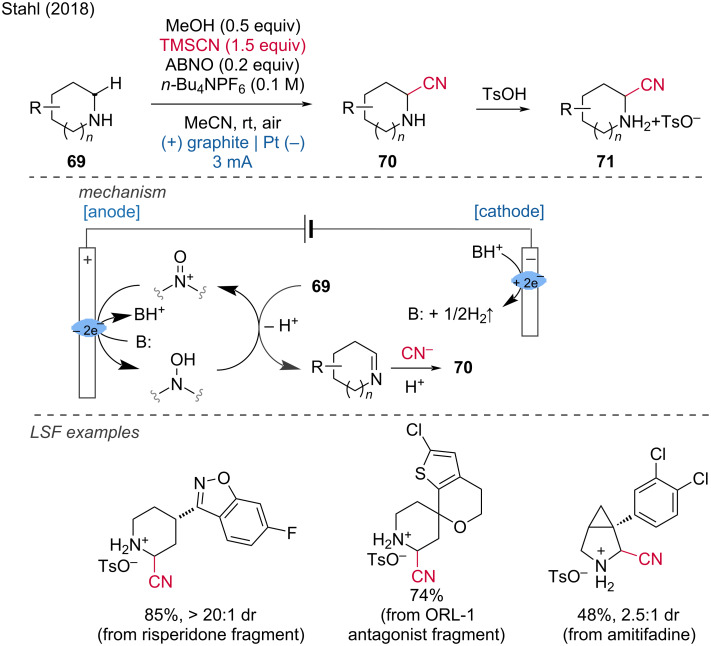
C(sp^3^)–H α-cyanation of secondary piperidines.

An electrochemical C(sp^3^)–H cyanation for LSF was reported by the Stahl group [37]. This protocol relies on the sterically encumbered ABNO (9-azabicyclononane *N-*oxyl) as a mediator, with TMSCN serving as the cyanide source. The reaction operates at low potentials, resulting in high functional group tolerance, even accommodating secondary alcohols. Additionally, pyrrolidine, anazepane, and morpholine scaffolds successfully underwent the reaction. Another notable feature of this method is its high diastereoselectivity. All products were ultimately obtained as *p*-toluenesulfonic acid salts (Scheme 27).

**Scheme 28:**
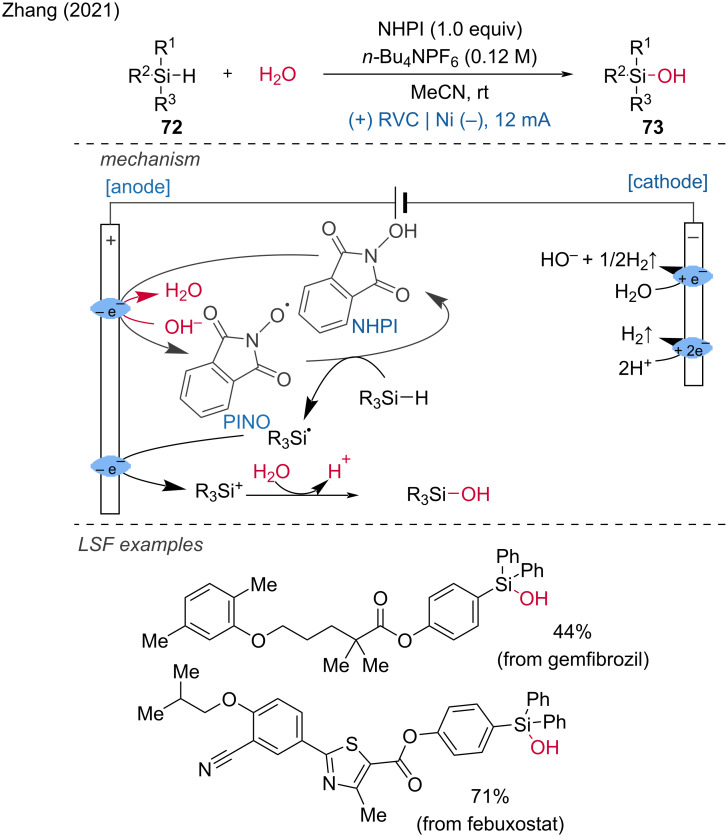
Selective electrochemical hydrolysis of hydrosilanes to silanols.

In 2021, Zhang et al. developed an electrochemical method for the hydrolysis of hydrosilanes to silanols using *N-*hydroxyphthalimide (NHPI) as the hydrogen-atom-transfer (HAT) mediator [38]. To demonstrate the potential of their approach, they showcased the LSF of natural products such as (−)-borneol and (+)-fenchol, as well as pharmaceutical drugs including ibuprofen, febuxostat, and gemfibrozil, achieving moderate to good yields. The proposed mechanism involves the oxidation and deprotonation of NHPI at the cathode to form phthalimide-*N-*oxyl (PINO) radicals. These PINO radicals act as HAT reagents, abstracting a hydrogen atom from the Si–H bond of the hydrosilane to generate a silyl radical. This silyl radical is then oxidized anodically to produce a silyl cation. The silyl cation subsequently abstracts a proton from water (H_2_O), forming the desired silanol product (Scheme 28).

**Scheme 29:**
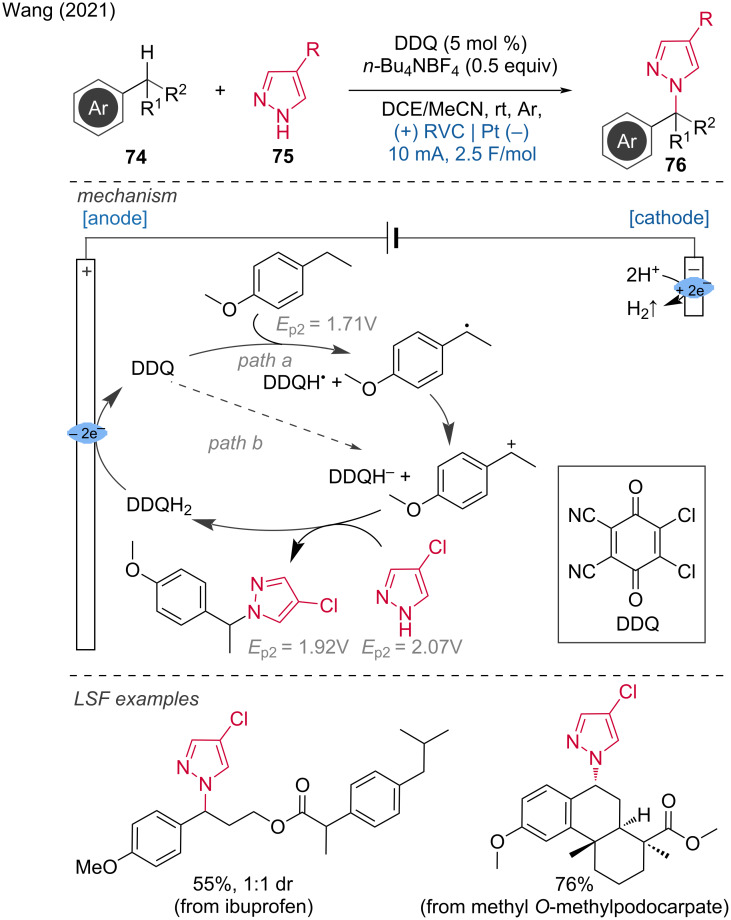
Organocatalytic electrochemical amination of benzylic C–H bonds.

While many methods combining metal catalysis and electrochemistry have been developed, the combination of electrochemistry with organocatalysis is generally less explored. In this context, Wang et al. combined organocatalysis and electrochemistry for the benzyl amination via C–H/N–H dehydrogenative cross-coupling of alkyl arenes with azoles [39]. According to the authors, the reaction proceeds via hydrogen-atom transfer (HAT) at the benzylic position, mediated by DDQ (2,3-dichloro-5,6-dicyano-1,4-benzoquinone). The proposed mechanism includes two possible pathways: In path A, the benzylic position undergoes HAT to form a benzyl radical, which is then oxidized by the DDQH^•^ radical to generate a carbocation and DDQH^−^. In path B, the reaction involves direct hydride transfer to DDQ, forming DDQH^−^ and a carbocation. In both pathways, the amine nucleophile captures the carbocation, resulting in the final amination product after losing a proton. Subsequently, DDQH^−^ is protonated to produce DDQH_2_. The anodic oxidation of DDQH_2_ regenerates DDQ, which re-enters the catalytic cycle (Scheme 29).

Furthermore, Qiu and coworkers disclosed a metal-free electrochemical dihydroxylation of unactivated alkenes using water as the hydroxy source under air conditions [40].

**Scheme 30:**
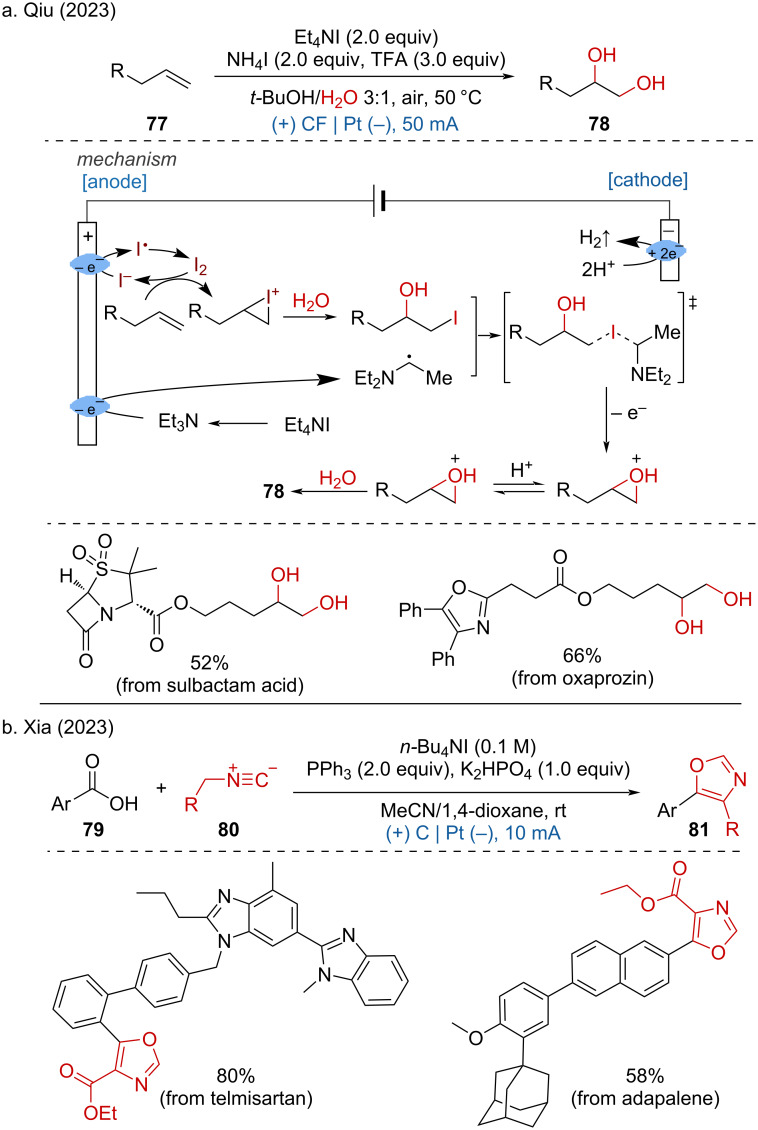
Iodide ion-initiated anodic oxidation reactions.

This mild method proceeds with a broad range of unactivated alkenes, including natural products and pharmaceutical derivatives such as sulbactam acid and oxaprozin. Mechanistic studies revealed that the reaction was initiated by the electrochemical oxidation of iodide ions, generating iodine radicals that dimerize to form iodine (I_2_). Subsequent anodic oxidation of in-situ formed Et_3_N produced an α-amino radical. The iodine then reacts with the alkene to form an iodonium intermediate, which undergoes intramolecular cyclization with losing an electron, and a second water attack to yield the desired product (Scheme 30a). In the same year, the Xia group reported an iodide ion and PPh_3_-induced electrochemical oxidative [3 + 2] cycloaddition of carboxylic acids and isocyanoacetates [41]. The successful LSF of drug molecules such as sulbactam acid and oxaprozin demonstrated the potential applicability of this method (Scheme 30b).

#### 1.3 Metal-assisted anodic oxidation

**Scheme 31:**
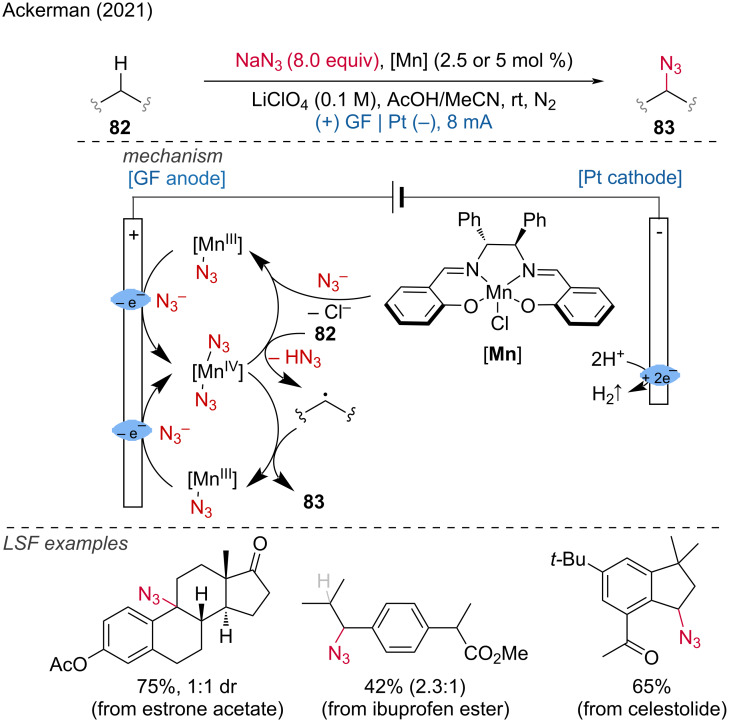
Mn(III/IV) electro-catalyzed C(sp^3^)–H azidation.

**1.3.1 Mn-assisted anodic oxidation.** The Ackermann group was the first to achieve an C–H azidation by use of a manganese-catalyzed anionic oxidation using “traceless electrons” [42]. By employing inexpensive sodium azide and a manganese salen complex, C(sp^3^)–H bonds underwent azidation with high chemoselectivity, even in the absence of a directing group. The proposed mechanism involves the formation of the active catalyst Mn(III)(N_3_) via ligand exchange, followed by anodic oxidation to a Mn(IV)(N_3_)_2_ complex. This high–valent Mn(IV) species undergoes hydrogen-atom transfer (HAT) leading to alkyl radical formation. The manganese-catalyzed azide radical transfer then delivers the product. Unactivated secondary and tertiary C–H bonds, as well as benzylic C–H bonds, were prone to azidation, with the reactivity order being: benzylic > tertiary > secondary. Functional groups such as silyloxy, amides, ethers, esters, enolizable ketones, and nitriles were found to be compatible with this transformation. Late-stage functionalization of different molecules was demonstrated; for example, the azidation of ibuprofen occurred preferentially at the secondary benzylic position (Scheme 31).

**1.3.2 Co-assisted anodic oxidation.** In 2021, Xu and colleagues developed an electrocatalytic approach for the intramolecular oxidative allylic amination and C–H alkylation using cobalt–salen complexes as catalysts [43]. In this reaction, the cobalt catalyst [Co(II)] is first oxidized to [Co(III)]^+^ at the anode, while MeOH undergoes cathodic reduction to form MeO^−^ and H_2_.

**Scheme 32:**
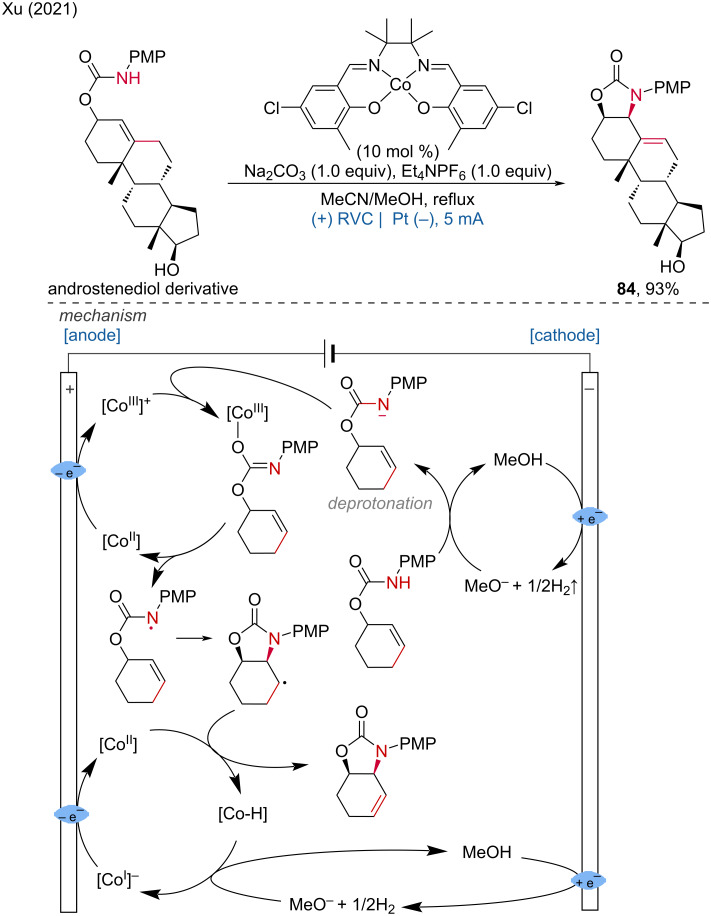
Tailored cobalt–salen complexes enable electrocatalytic intramolecular allylic C–H functionalizations.

**Scheme 33:**
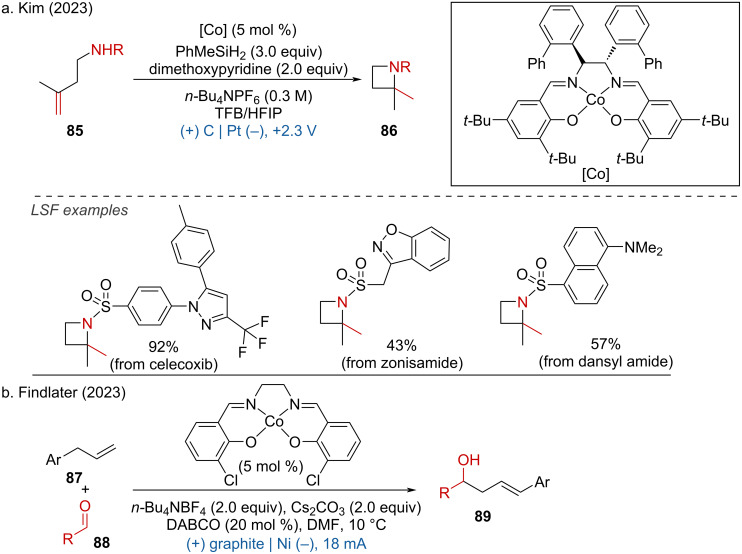
Cobalt–salen complexes-induced electrochemical (cyclo)additions.

The MeO^−^ then deprotonates the carbamate, and the resulting conjugated base is oxidized by the cobalt–salen complex [Co(III)]^+^, generating an amide radical. This amide radical initiates radical cyclization to form a cyclic alkyl radical. The alkyl radical is further oxidized by [Co(III)] to produce the target amination product and a [Co(II)–H] species via direct hydrogen transfer or β-hydride elimination. Deprotonation of [Co(II)–H] by MeO^−^ regenerates the [Co(I)] complex, which is subsequently oxidized back to [Co(II)] at the anode (Scheme 32). Recently, two additional studies on cobalt–salen complex-induced (cyclo)additions were reported by the Kim [44] and Findlater groups [45]. By employing cobalt–salen as a catalyst, along with PhMeSiH_2_ and dimethoxypyridine as additives, *n*-Bu_4_NPF_6_ as the electrolyte, and carbon felt and platinum plate as electrodes, the intramolecular hydroamination proceeded smoothly, yielding azetidines in moderate to good yields. This method was applied to the LSF of celecoxib, zonisamide, and dansyl amide (Scheme 33a). Additionally, the allylation of aldehydes also proceeded efficiently (Scheme 33b).

**Scheme 34:**
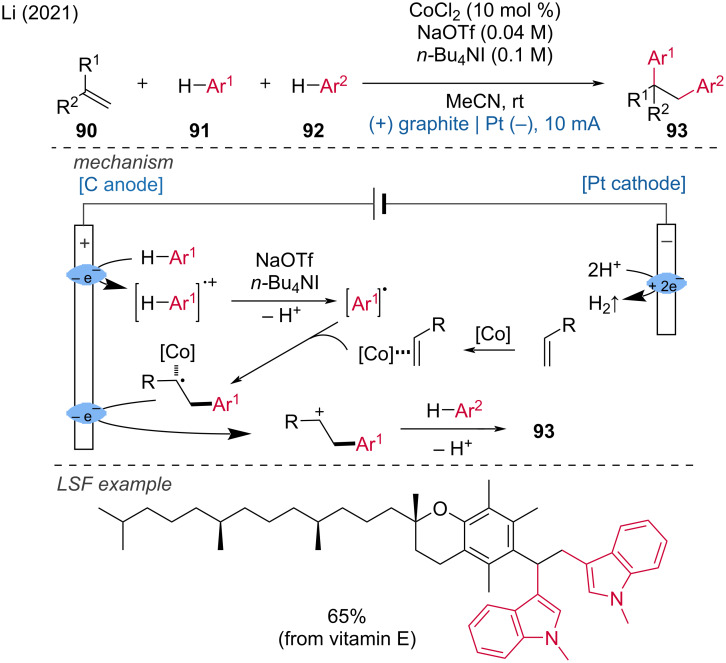
Electrochemical 1,2-diarylation of alkenes enabled by direct dual C–H functionalization of electron-rich aromatic hydrocarbons.

The use of cobalt salts in combination with electrochemistry has also been applied to difunctionalization reactions. Li and coworkers developed a protocol utilizing a Co catalyst for the electrochemical 1,2-diarylation of alkenes with electron-rich aromatic hydrocarbons, employing a radical relay strategy to produce polyaryl-functionalized alkanes [46]. The authors proposed that the initial anodic oxidation of indole generates an indole cation radical intermediate, which is successively deprotonated to form an indolyl carbon-centered radical. This radical then adds to the C=C bond in the Co–alkene complex, forming an intermediate alkyl radical, which is further anodically oxidized to produce an intermediate alkyl cation. Another indole molecule undergoes electrophilic alkylation by this intermediate, forming an indolyl cation, which upon deprotonation yields the final product (Scheme 34).

**Scheme 35:**
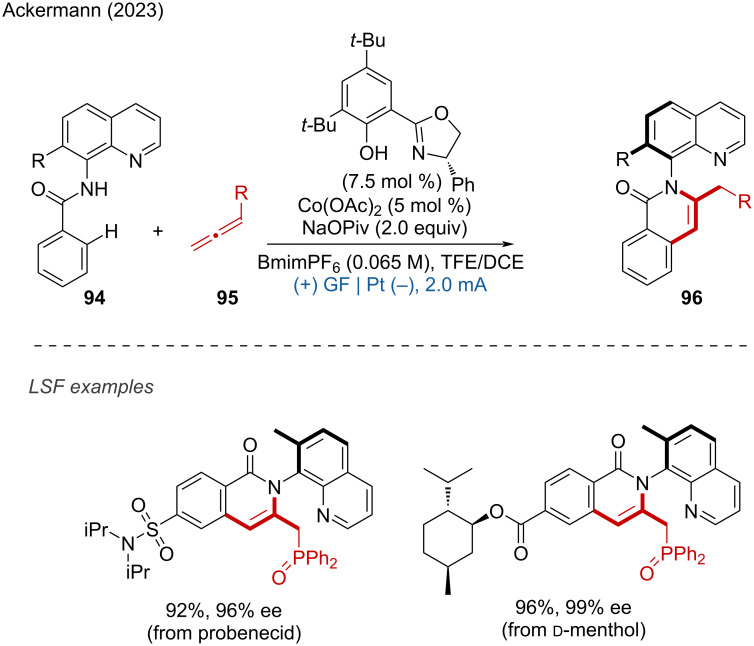
Cobalt-electrocatalyzed atroposelective C–H annulation.

To date, only a few enantioselective reactions using metal catalysis and electrochemistry have been reported. Very recently, Ackermann and coworkers employed Co(OAc)_2_ as a catalyst and a salicyloxazoline derivative as a chiral ligand to achieve the electrochemical atroposelective C–H annulation of benzamides and allenes [47]. This method demonstrated excellent functional group tolerance, yielding a broad range of C–N axially chiral compounds with good yields and enantioselectivities. The practicality of this strategy was further demonstrated by a decagram-scale synthesis and the LSF of complex compounds (Scheme 35). In addition, Niu and coworkers reported a similar work on a cobalt-electrocatalyzed atroposelective C–H annulation of benzamides with acetylenes [48].

**Scheme 36:**
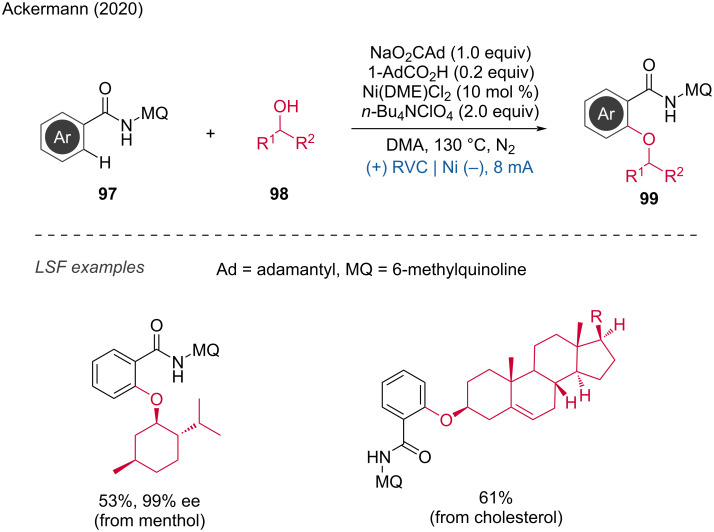
Nickel-electrocatalyzed C(sp^2^)–H alkoxylation with secondary alcohols.

**1.3.3 Ni-assisted anodic oxidation:** Apart from cobalt, nickel complexes have also been applied in anionic oxidations and late-stage functionalizations. In 2020, Ackermann and coworkers reported the challenging C–H alkoxylation of (hetero)arenes with sterically encumbered secondary alcohols via a nickel electrocatalyzed protocol [49]. A traceless removable quinoline amide in the meta position was employed as a directing group. Based on extensive mechanistic studies, they proposed the formation of a formal Ni(IV) complex during the process. Remarkably, nickel proved to be uniquely effective for this protocol, as other transition-metal catalysts based on Cu, Co, Pd, Ir, Ru, and Rh did not catalyze the reaction (Scheme 36).

**Scheme 37:**
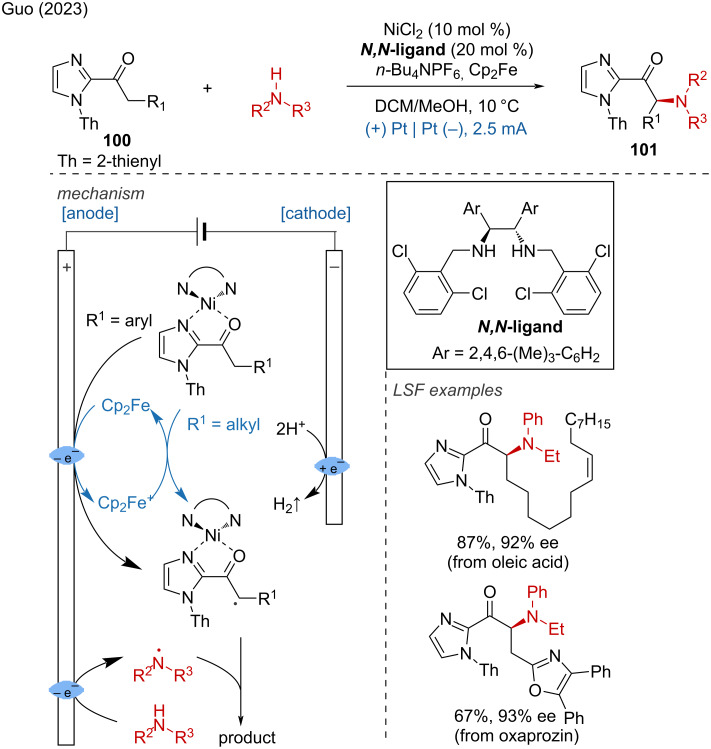
Nickel-catalyzed electrochemical enantioselective amination.

In 2023, the Guo group reported the enantioselective cross-dehydrogenative amination via electrochemical oxidation of C–H and N–H bonds, successfully achieved the LSF of several bioactive molecules and natural products with good yields and high stereoselectivities [50]. The plausible catalytic mechanism starts with the formation of a nickel-chelated enolate intermediate, followed by the anodic oxidation to form the nickel-coordinated carbon-centered radical intermediate. Another mechanism is proposed for alkyl-substituted acylimidazoles. In this case, the additive ferrocene (Cp_2_Fe) serves as mediator between the anode and the nickel-chelated enolate intermediate. Simultaneously, the amine substrate is oxidized at the anode and deprotonated to generate a nitrogen-centered radical. The desired product then is generated by the stereoselective cross-coupling of the carbon-centered radical with the nitrogen-centered radical (Scheme 37).

**Scheme 38:**
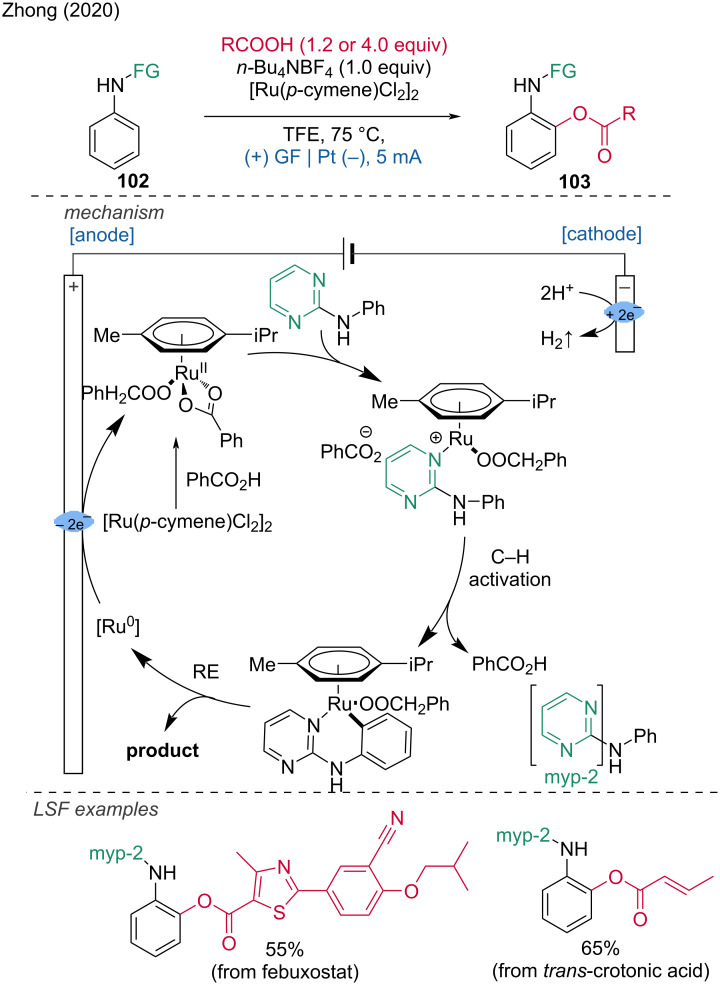
Ruthenium-electrocatalyzed C(sp^2^)–H mono- and diacetoxylation.

**1.3.4 Ru-assisted anodic oxidation.** A ruthenium electrocatalyzed mono- and diacetoxylation of aniline derivatives via a C(sp^2^)–H functionalization was developed by Zhong and coworkers [51]. This transformation requires the presence of a removable directing group bonded to the nitrogen atom of the aniline substrate. The methodology showcases a broad scope for carboxylic acids and demonstrates multiple examples of LSF of pharmaceuticals and natural products. The proposed mechanism begins with the formation of Ru(II) diacetate through ligand exchange between the carboxylate substrate and [Ru(*p*-cymene)Cl_2_]_2_. Subsequently, the Ru complex coordinates with the aniline substrate, followed by C–H activation to form a six-membered Ru species. The final product is generated through reductive elimination, releasing Ru(0), which is then reoxidized on the anode to regenerate the active Ru(II) species, completing the catalytic cycle (Scheme 38). This approach underlines the potential of ruthenium catalysis in achieving site-selective functionalization of complex molecules, thereby expanding the toolkit available for organic synthesis and drug development.

**1.3.5 Rh-assisted anodic oxidation.** In addition to ruthenium-catalyzed electrochemically mediated C–H functionalizations, several groups have also explored rhodium-catalyzed anodic oxidation reactions [52–53]. Wen, Zhang, Xu, and colleagues described an efficient method for the phosphorylation of aryl substrates utilizing a Rh(III) catalyst [54].

**Scheme 39:**
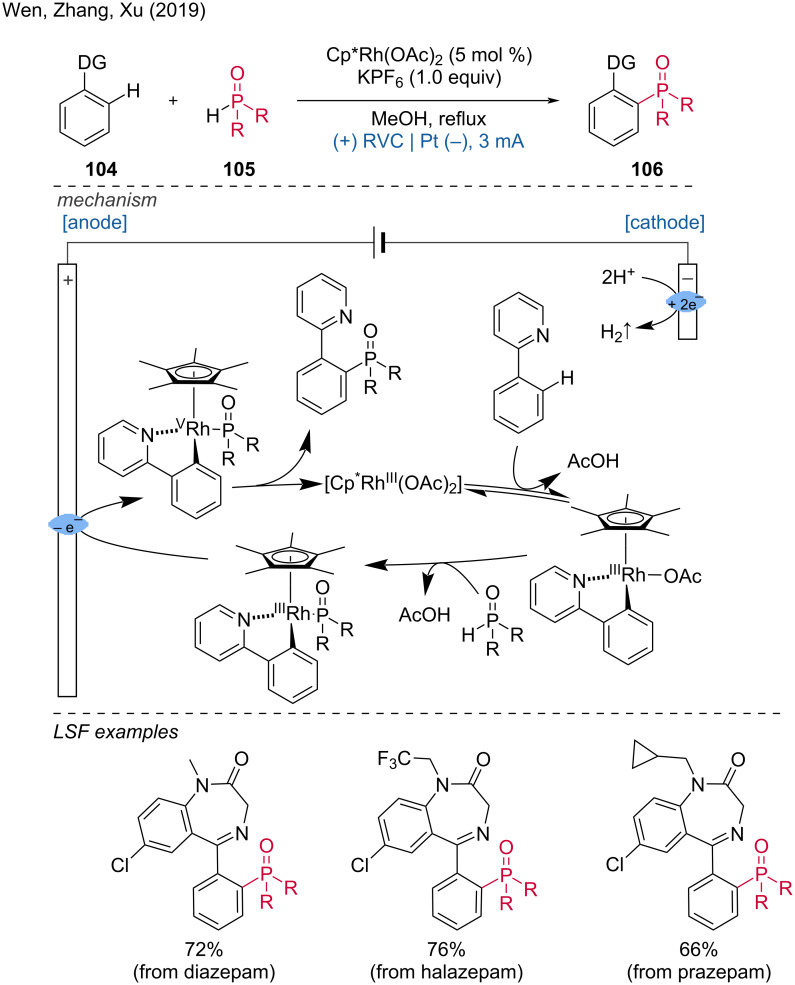
Rhodium(III)-catalyzed aryl-C–H phosphorylation enabled by anodic oxidation-induced reductive elimination.

The critical step in this process is the anodic oxidation of Rh(III) to a high-valent Rh complex on the RVC anode. This transformation necessitates the presence of a directing group in the substrate molecule. Cyclic ketimine was found to direct *ortho*-C–H phosphorylation efficiently, enabling the LSF of benzodiazepine drugs such as diazepam, halazepam, and prazepam. Mechanistically, the Rh complex activates the C–H bond in phenylpyridine and assists in forming a bond with diphenylphosphine oxide, which, after anodic oxidation, yields the target product (Scheme 39).

**Scheme 40:**
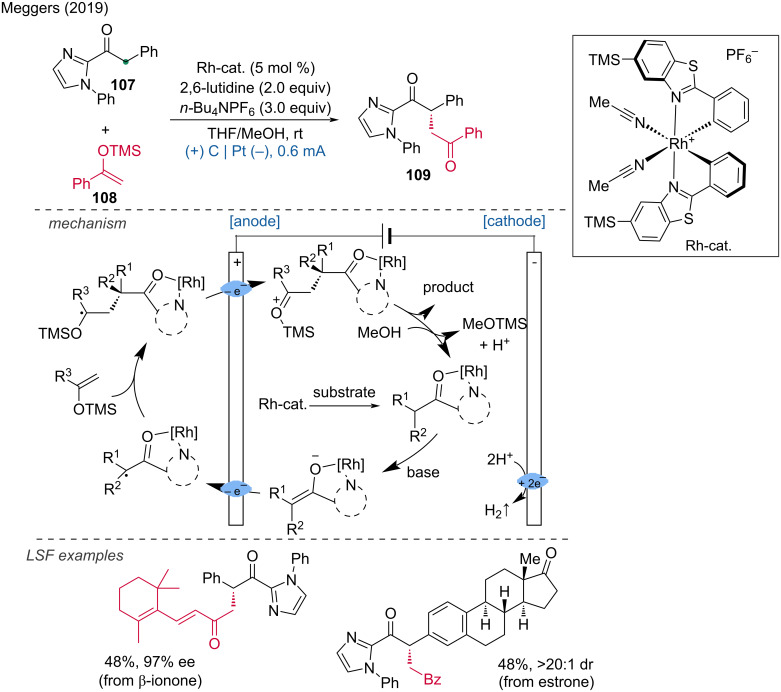
Asymmetric Lewis-acid catalysis for the synthesis of non-racemic 1,4-dicarbonyl compounds.

A chiral Lewis acid complex of Rh has been employed by Meggers and coworkers to functionalize the α-position of 2-acylimidazoles [55]. The reported transformation represents a successful example of a catalytic asymmetric electrosynthesis, which is typically quite challenging. The process was conducted in an undivided cell using a boron-doped diamond (BDD) anode and a platinum cathode at constant current, resulting in 1,4-dicarbonyls with yields up to 91% and enantiomeric excesses (ee) greater than 99%. This methodology was demonstrated in the LSF of two complex natural product derivatives: a β-ionone derivative and an estrone derivative. Mechanistically, the process begins with the coordination of a chiral rhodium-based catalyst to the 2-acylimidazole substrate. Deprotonation by the base 2,6-lutidine activates the substrate for anodic oxidation by raising the level of the highest occupied molecular orbital during enolate formation, thus providing mild redox conditions. After anodic oxidation, a carbon-centered radical at the α-position is formed, which undergoes stereocontrolled C–C-bond formation with the silyl ether, forming a trimethylsilyl (TMS)-ketyl radical. A second anodic one-electron oxidation then yields the intermediate. Subsequent desilylation and substrate/product exchange complete the catalytic cycle (Scheme 40). This approach underlines the potential of asymmetric electrosynthesis in achieving high selectivity and efficiency in complex molecule synthesis, further broadening the applications of electrochemical methods in organic synthesis.

**Scheme 41:**
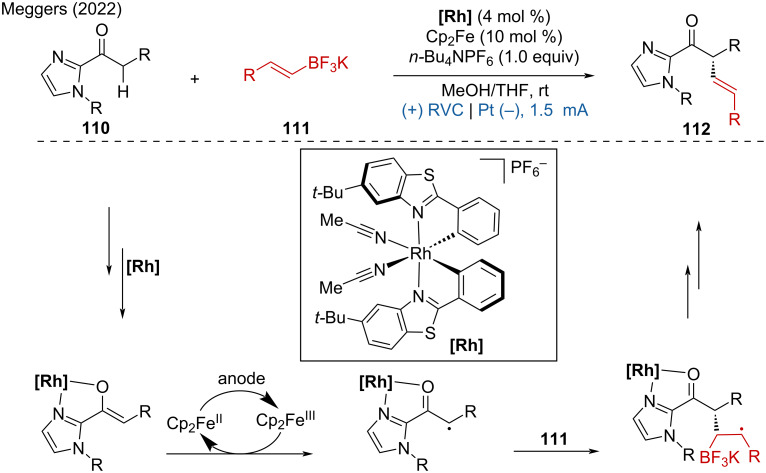
Electrochemical enantioselective C(sp^3^)–H alkenylation.

In this context the Meggers group developed an asymmetric Rh catalyst-promoted alkylation [56]. The Rh complex was used as a chiral catalyst and Cp_2_Fe as an anodic oxidation catalyst to achieve the enantioselective C(sp^3^)–H alkenylation of 2-acylimidazoles with potassium alkenyl trifluoroborates under mild electrochemical conditions (Scheme 41). This method provides an efficient and enantioselective approach to C(sp^3^)–H alkenylation, demonstrating the potential of combining chiral catalysis with electrochemistry for the functionalization of complex molecules.

**Scheme 42:**
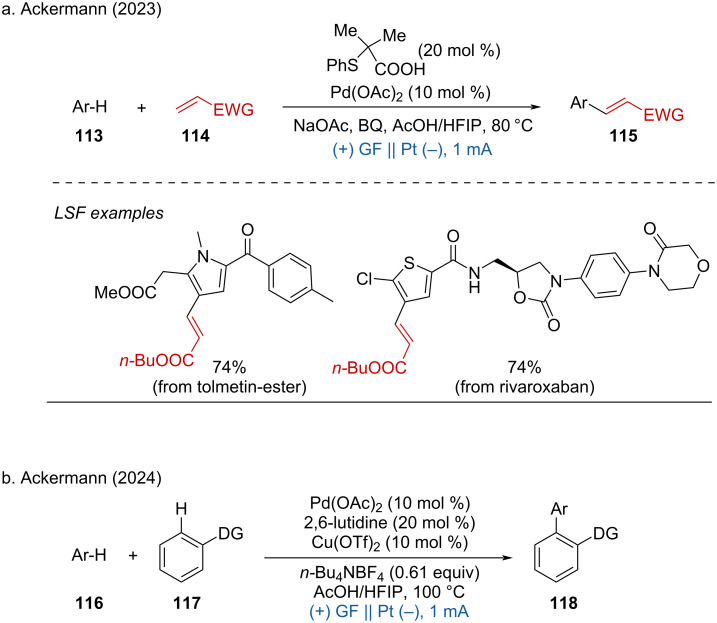
Palladium-catalyzed electrochemical dehydrogenative cross-coupling.

**1.3.6 Pd-assisted anodic oxidation.** In 2023, Ackermann and coworkers reported a Pd-catalyzed anodic oxidation for the alkenylation of arenes without the need for directing groups [57]. Using Pd(OAc)_2_ as the catalyst, 2-methyl-2-(phenylthio)propanoic acid as the ligand, and 1,4-benzoquinone (BQ) as the redox mediator, this method showed excellent tolerance across various arenes and alkenes. The selective LSF of biorelevant complex molecules demonstrated significant potential for drug exploration (Scheme 42a). One year later, the same group disclosed the dehydrogenative cross-coupling of two arenes via palladium-catalyzed electrooxidation, further showcasing the versatility and potential of this approach in organic synthesis (Scheme 42b) [58]. These methodologies underline the expanding role of palladium catalysis in electrochemical transformations, offering robust strategies for the functionalization of complex molecules without the need for directing groups, thereby simplifying the synthesis process and enhancing the exploration of new drug candidates.

**1.3.7 Ir-assisted anodic oxidation.** An Ir-electrocatalyzed vinylic C(sp^2^)–H activation method for the preparation of α-pyrones via annulation of acrylic acids with alkynes was reported by Mei and coworkers [59]. Diverse functional groups on the aryl group connected to the alkyne are compatible with this transformation, and dialkylalkynes can also be effectively reacted. Extensive mechanistic studies have led to the following proposed mechanism.

**Scheme 43:**
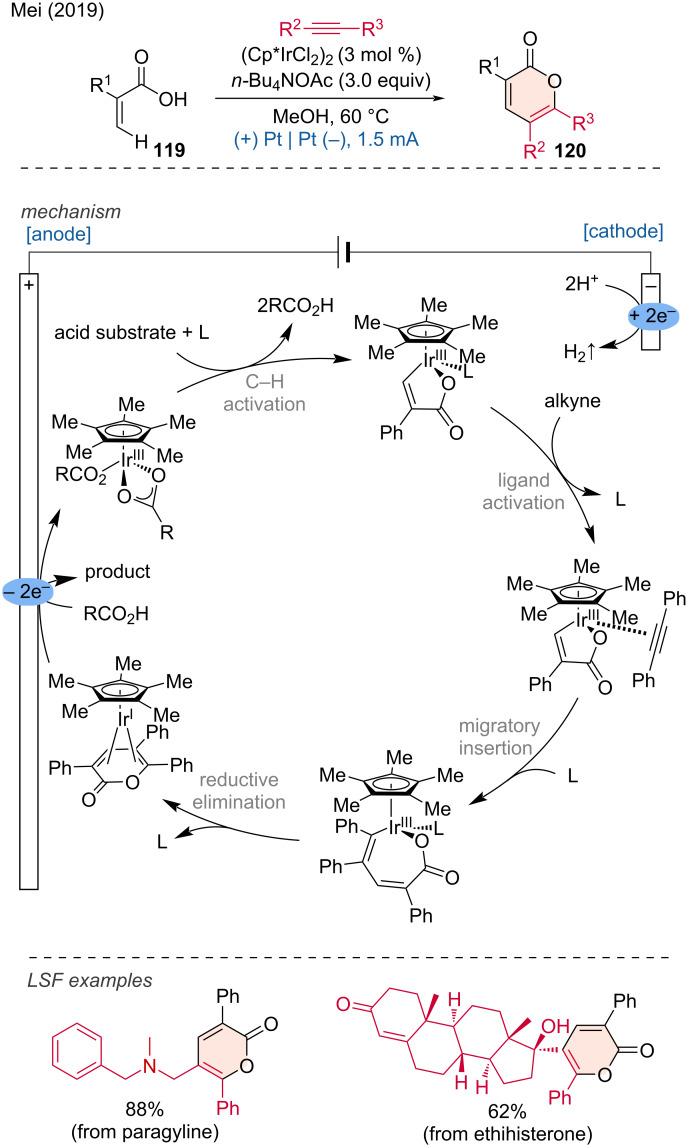
Ir-electrocatalyzed vinylic C(sp^2^)–H activation for the annulation between acrylic acids and alkynes, forming α-pyrones.

Initially, C–H activation occurs, resulting in the formation of a cyclometalated Ir(III) intermediate. Ligand exchange with the alkyne substrate, followed by migratory insertion, leads to the formation of a seven-membered 18-electron Ir(III) complex. This complex then undergoes reductive elimination (RE) to produce an 18-electron Ir(I) complex. The Ir(I) complex is subsequently anodically oxidized back to an Ir(III) complex, with the concomitant elimination of the product. This protocol can be applied to the LSF and diversification of natural products, as demonstrated by the examples of pargyline and ethisterone (Scheme 43).

**Scheme 44:**
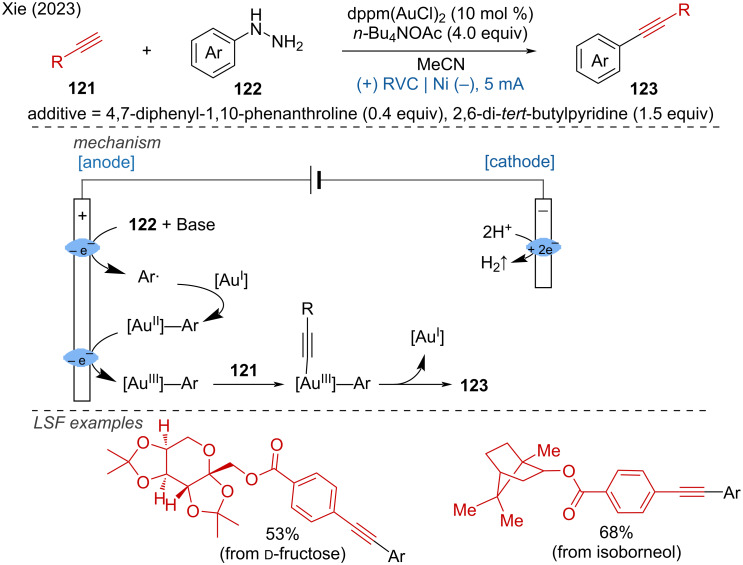
Electrochemical gold-catalyzed C(sp^3^)–C(sp) coupling of alkynes and arylhydrazines.

**1.3.8 Au-assisted anodic oxidation.** A gold-catalyzed C(sp^3^)–C(sp) coupling of diverse alkynes and arylhydrazines under mild electrochemical conditions with the dinuclear gold complex, bis(diphenylphosphino)methane digold(I) dichloride (dppm(AuCl)_2_) as catalyst and *n*-Bu_4_NOAc as electrolyte was developed Xie and coworkers [60]. The reaction showed excellent functional group compatibility and biocompatibility, the LSF of biomolecules such as ᴅ-fructose and isoborneol further proved the synthetic robustness (Scheme 44).

#### 1.4 Anodic photoelectrochemical oxidation

Combining electro- and photocatalysis can lead to highly precise electron-driven reactions that are otherwise inaccessible [61–67]. The photoelectrochemical method for the alkylation of C–H heteroarenes using organotrifluoroborates, developed by Xu and coworkers, has demonstrated excellent results in this respect [68]. This C–H photoelectrochemical functionalization reaction proved to be a mild method, as shown in the alkylation of drug derivatives such as voriconazole and quinine, along with excellent regio- and chemoselectivity.

**Scheme 45:**
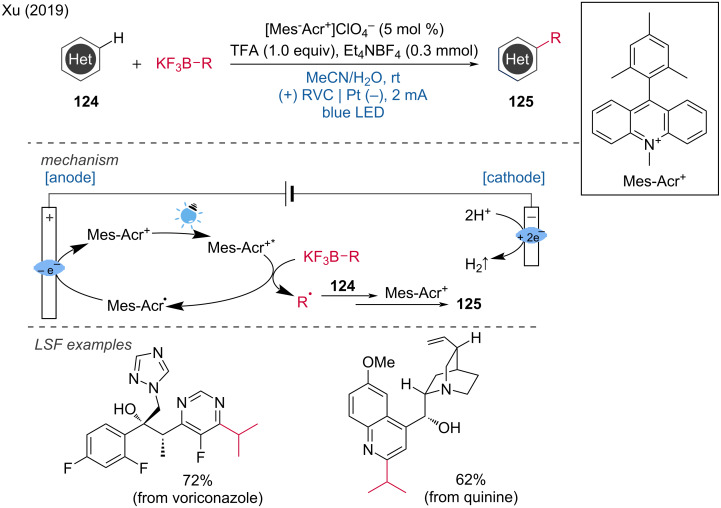
Photoelectrochemical alkylation of C–H heteroarenes using organotrifluoroborates.

The reaction pathway begins with the photoexcitation of Mes-Acr^+^ (9-mesityl-10-methylacridinium) to yield the photoexcited Mes-Acr^+^* (*E*_ox_ = +2.06 V vs. SCE), which then oxidizes the trifluoroborate (*E*_ox_ ≈ +1.50 V vs. SCE) to a radical. This radical reacts with the protonated heteroarene to form a radical cation, which subsequently loses a proton and converts into a carbon radical intermediate. The photocatalyst then oxidizes this intermediate, leading to the final product (Scheme 45). This approach underscores the significant potential of combining electro- and photocatalysis to achieve selective and mild transformations in organic synthesis, particularly in the late functionalization of complex drug molecules.

Due to the versatility of the azide group, the direct C(sp^3^)–H azidation is an extremely valuable transformation. Lei and coworkers achieved this transformation using electrochemistry upon irradiation with blue LEDs [69].

**Scheme 46:**
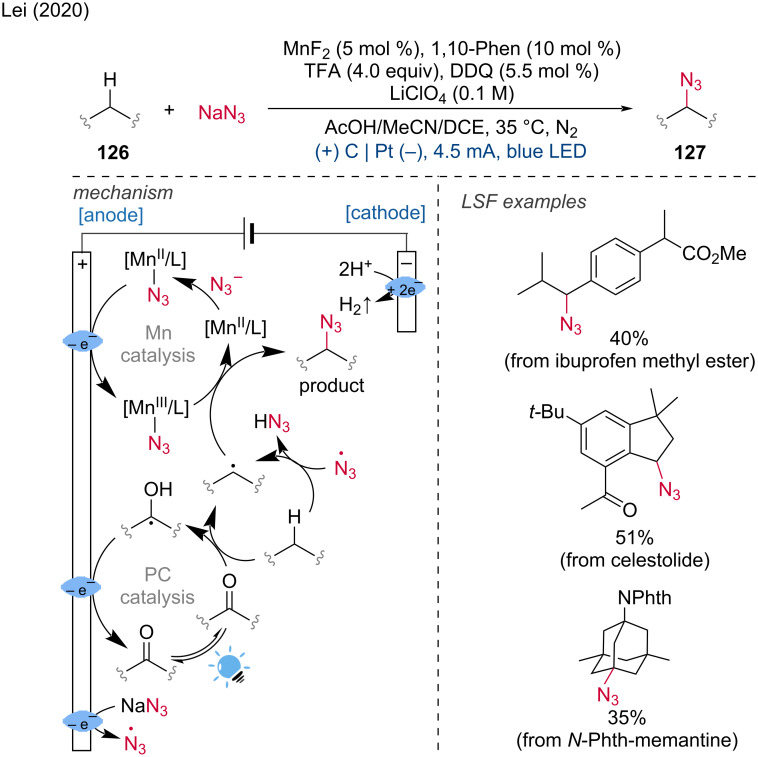
Mn-catalyzed photoelectro C(sp^3^)–H azidation.

Under photoelectric conditions, a combination of a manganese (Mn) catalyst and NaN_3_ delivered the azidation product. This protocol was effective for azidating unactivated secondary and tertiary carbon bonds as well as benzylic C–H bonds and was applied to the LSF of certain drugs and natural products. Mechanistic studies led the authors to propose the following mechanism: The process begins with the anodic oxidation of Mn(II) coordinated with N_3_^−^ to generate a Mn(III) species. In the photocatalytic cycle, the excitation of DDQ (2,3-dicyano-5,6-dichlorobenzoquinone) as hydrogen-atom-transfer (HAT) catalyst results in the formation of an alkyl radical. The HAT catalyst is regenerated via anodic oxidation. The final product is formed through azide radical transfer from the Mn catalyst. Notably, the alkyl radical can also possibly be formed by HAT with the azide radical generated through anodic oxidation. The mechanism involved a Mn(II)/Mn(III) cycle at the anode, which differs from Ackermann’s reported Mn(III)/Mn(IV) cycle at the anode (Scheme 46).

**Scheme 47:**
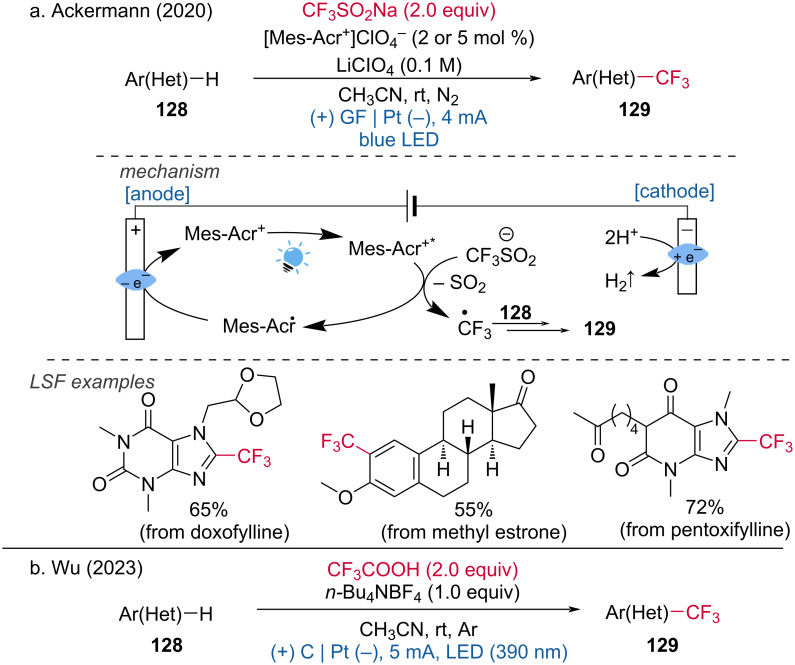
Photoelectrochemical undirected C–H trifluoromethylations of (Het)arenes.

A photoelectrochemistry method for the C–H trifluoromethylation of arenes using the Langlois trifluoromethylation reagent (CF_3_SO_2_Na) was developed by Ackermann and coworkers [70]. The mildness of this reaction was demonstrated through the late-stage C–H trifluoromethylation of ascapheine, pentoxifylline, doxophylline, theobromine, methylethrone, and tryptophan derivatives. During the reaction, irradiation of the organic dye Mes-Acr^+^ leads to the formation of its oxidized excited state, Mes-Acr^+^*. This excited state then reductively interacts with the sulfinate anion to produce a CF_3_ radical. The CF_3_ radical subsequently attacks the target substrate, forming an intermediate radical, which undergoes further oxidation to yield the desired product (Scheme 47a). The Wu group disclosed a similar trifluoromethylation of arenes under photoelectrochemical reaction conditions but without the addition of a photocatalyst, using trifluoroacetic acid as the CF_3_ source (Scheme 47b) [71]. This alternative approach further underscores the versatility and applicability of trifluoromethylation techniques in organic synthesis, expanding the toolkit available for modifying complex molecules.

**Scheme 48:**
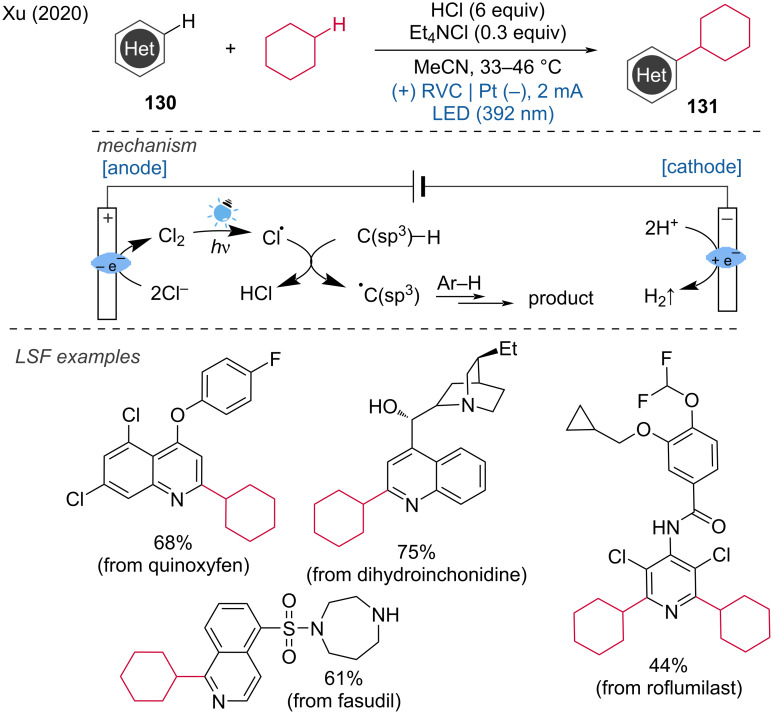
Photoelectrochemical dehydrogenative cross-coupling of heteroarenes with aliphatic C–H bonds.

Furthermore, Xu and coworkers developed a photoelectrochemical method for alkylation via a dehydrogenative cross-coupling of heteroarenes [72]. In this transformation, chlorine is formed at the anode, which is then photochemically homolyzed by light (392 nm) to produce chlorine radicals. These chlorine radicals act as hydrogen-atom-transfer (HAT) reagents to form alkane radicals. The resulting alkyl radicals then undergo a Minisci-type reaction, resulting in alkylated heteroarene products. This method eliminates the need for a metal catalyst or a chemical oxidizer and demonstrates broad compatibility with a variety of heteroarenes as well as activated and non-activated C(sp^3^)–H donors (Scheme 48). This valuable approach highlights the potential of combining photoelectrochemical techniques to achieve efficient and selective transformations in organic synthesis, particularly for the functionalization of heteroarenes.

In this context, Lambert and Shen proposed an photoelectrochemical Ritter-type reaction for the amination of benzyl C–H bonds using trisaminocyclopropenium (TAC) ion as a catalyst [73]. This approach minimizes the risk of adverse outcomes by utilizing cell potentials that are insufficient to oxidize the substrate directly, thus allowing selective one-electron oxidation by the electrophotocatalyst. A divided cell with a carbon felt anode and a platinum cathode is employed under constant voltage (CV) conditions and light irradiation under a nitrogen atmosphere.

**Scheme 49:**
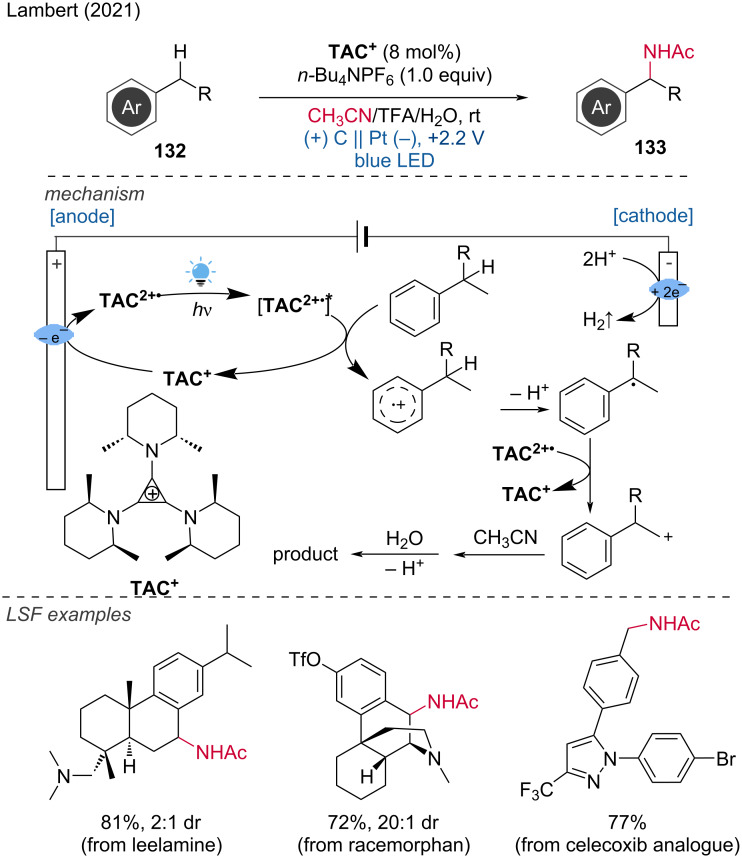
C–H amination via photoelectrochemical Ritter-type reaction.

This method demonstrates broad compatibility with various functional groups and complex substrates, including alcohol, carboxylic acid, esters, alkyl chloride, and tosylate groups. Notably, the LSF of compounds such as leelamine, racemorphan, and analogs of sertraline and celecoxib was achieved with yields ranging from 40% to 92%. The reaction mechanism begins with the photoexcitation of the intermediate [TAC^2+•^]*, which oxidizes the arene substrate to form a cation radical. This radical is deprotonated and then further oxidized, either directly at the anode or by the TAC dication radical. The resulting intermediate undergoes the classic Ritter steps, reacting with acetonitrile to form a nitrile, which is subsequently hydrolyzed to yield the target amide product (Scheme 49).

**Scheme 50:**
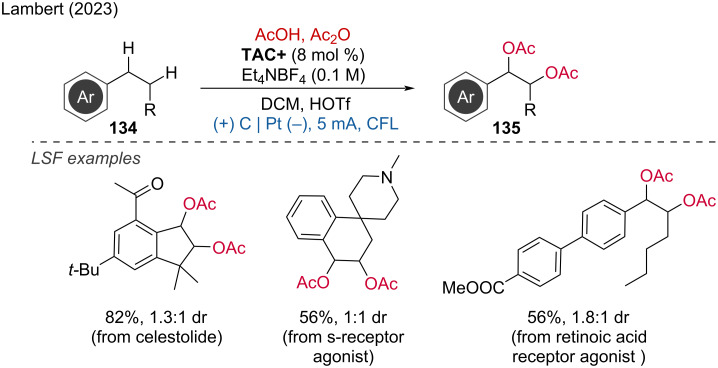
Photoelectrochemical multiple oxygenation of C–H bonds.

The construction of multiple C–O bonds from C–H bonds is challenging due to the risk of overoxidation. Recently, Lambert and coworkers explored the photoelectrochemical multiple oxygenation of C–H bonds using trisaminocyclopropenium (TAC^+^) as a photocatalyst [74]. This method enables the transformation of alkylarenes into the corresponding di- or triacetoxylates, including the LSF of bioactive compound analogues (Scheme 50).

The mechanism involves the photoexcitation of TAC^+^, which facilitates the selective oxidation of C–H bonds in alkylarenes. This process efficiently forms multiple C–O bonds while minimizing the risk of overoxidation. The versatility and mildness of this method were demonstrated by successfully applying it to various substrates, including bioactive compound analogues. This advancement highlights the potential of electrophotocatalysis in achieving complex transformations in organic synthesis, particularly in the selective oxygenation of C–H bonds, thus providing a valuable tool for the functionalization of complex molecules.

**Scheme 51:**
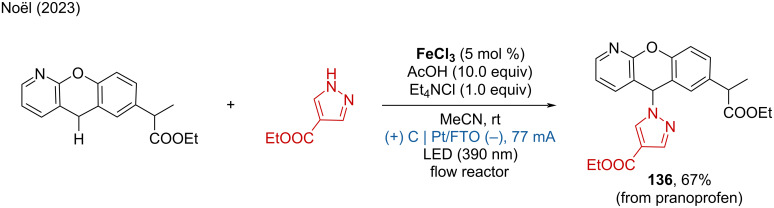
Accelerated C(sp^3^)–H heteroarylations by the f-EPC system.

A combined photoelectrochemical transformation with flow chemistry was developed by the Noël group. The flow electrophotocatalysis (f-EPC) system using FeCl_3_ as photocatalyst accelerates the C(sp^3^)–H heteroarylations [75]. For example, the heteroarylations of the nonsteroidal anti-inflammatory drug pranoprofen was accomplished, producing the desired product in 67% yield only within 30 min (Scheme 51). The f-EPC system integrates the advantages of electrophotocatalysis and flow chemistry, providing a rapid and efficient method for C–H functionalization. The continuous flow setup allows for precise control over reaction conditions, enhanced mass transfer, and improved reaction kinetics, leading to higher efficiency and faster reaction times.

**Scheme 52:**
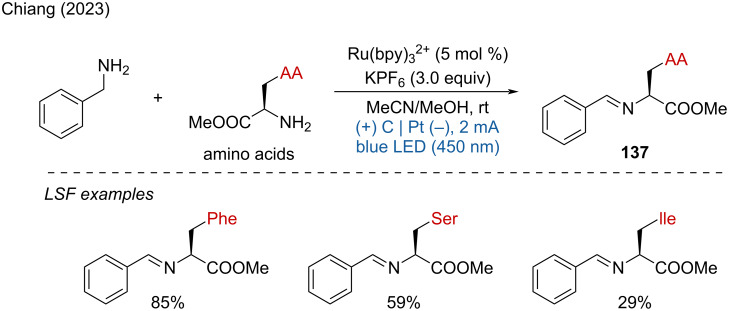
Photoelectrochemical cross-coupling of amines.

In 2023, the Chiang group reported the photoelectrochemical homo-coupling and cross-coupling of different kinds of amines for approaching symmetric and unsymmetric imines [76]. This method achieved the bioconjugation of several amino acids with benzylamine, the use of phenylalanine (Phe), serine (Ser), and isoleucine (Ile) as substrates led to 85%, 59%, and 29% yield, respectively. However, other amino acids such as glycine (Gly), histidine (His), and tyrosine (Try) resulted in much lower yields (Scheme 52).

### 2 LSF via cathodic reduction

Electrochemical cathodic reduction equipped with an anode as sacrifice material, avoids the use of external stoichiometric reducing agents, such as Mn and Zn. In the field of electrosynthesis, cathodic reduction methods are much less developed compared to anodic oxidation. However, cathodic reduction offers significant advantages, including milder reaction conditions, improved safety, and reduced environmental impact due to the avoidance of hazardous chemicals. As such cathodic reduction methods can lead to more sustainable and efficient processes for various transformations, such as the reduction of functional groups, hydrogenation, and the activation of small molecules.

#### 2.1 Direct cathodic reduction of substrates

**Scheme 53:**
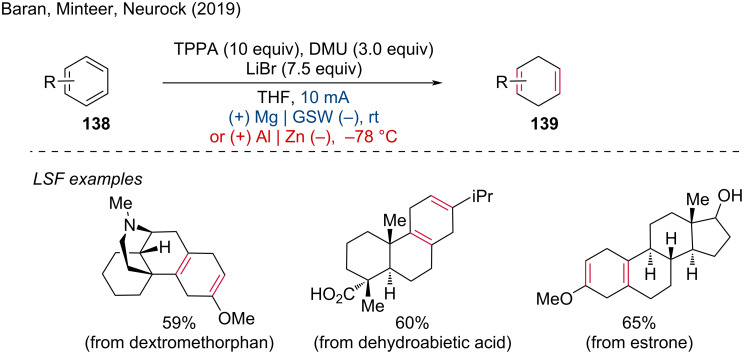
Birch electroreduction of arenes. GSW = galvanized steel wire.

**2.1.1 Electroreduction of unsaturated bonds.** In 2019, Baran, Minteer, Neurock, and coworkers disclosed a scalable electrochemical Birch reduction of arenes, providing a safe alternative to the classical Birch reduction by employing inexpensive magnesium or aluminum as the sacrificial anode [77]. The mild reaction conditions of this method were demonstrated through the chemo- and regioselective reduction of 1,4-dienyl derivatives of dextromethorphan, dehydroabietic acid, and estrone methyl ester (Scheme 53). This electrochemical Birch reduction offers several advantages over traditional methods, including enhanced safety by avoiding the use of hazardous reagents like sodium or lithium in liquid ammonia, scalability for industrial applications, and mild conditions that allow for the selective reduction of sensitive and complex molecules without affecting other functional groups.

**Scheme 54:**
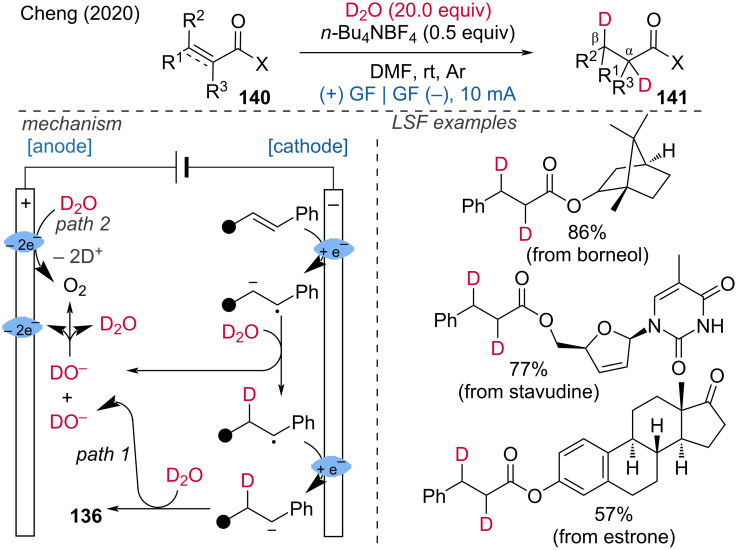
Electroreductive deuterations.

In this context, Cheng and colleagues have developed a mild electrochemical deuteration method using graphite felt electrodes [78]. The reaction utilizes inexpensive D_2_O as a deuterium source and requires neither a transition-metal catalyst nor a stoichiometric reducing agent. The use of graphite felt as both the cathode and anode is crucial for achieving high chemoselectivity and efficient deuterium incorporation. Mechanistic experiments have shown that the release of O_2_ at the anode eliminates the need for an external reducing agent and regulates the pH of the reaction mixture, maintaining it at approximately neutral. This method has been successfully applied to obtain several deuterated pharmaceutical compounds (Scheme 54).

Recently, the Qiu group disclosed two significant advancements in the field of electrochemical reductive deuteration reactions. First, they reported the reductive deuteration of unactivated alkyl halides under electrochemical reaction conditions [79]. Following this, they developed a triphenylphosphine (TPP)-mediated electrochemical reductive deuteration of styrenes [80].

**Scheme 55:**
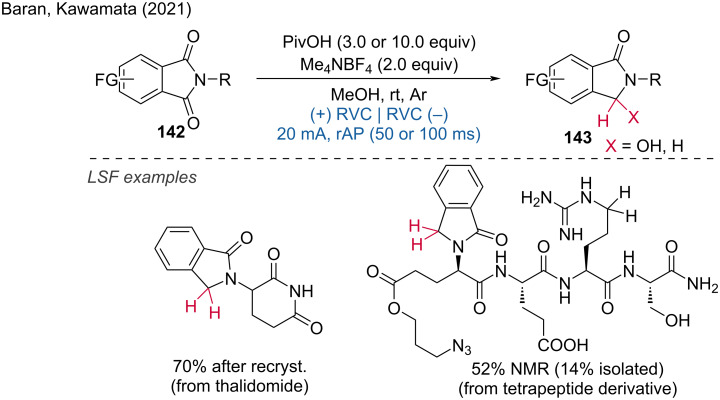
Chemoselective electrosynthesis using rapid alternating polarity.

In 2021, Kawamata and Baran further expanded the possibilities of electroreduction by employing rapid alternating polarity (rAP) electric current with a square current waveform for the highly chemoselective reduction of carbonyl compounds [81]. The synthetic utility of rAP was demonstrated through the successful late stage reduction of unprotected thalidomide on a gram scale (Scheme 55). This approach underscores the potential of rAP in achieving high chemoselectivity and efficiency in the reduction of carbonyl compounds, highlighted by its applicability in the synthesis of complex molecules. The ability to perform such reductions on a gram scale further emphasizes the practical utility of this method in industrial and pharmaceutical settings.

**Scheme 56:**
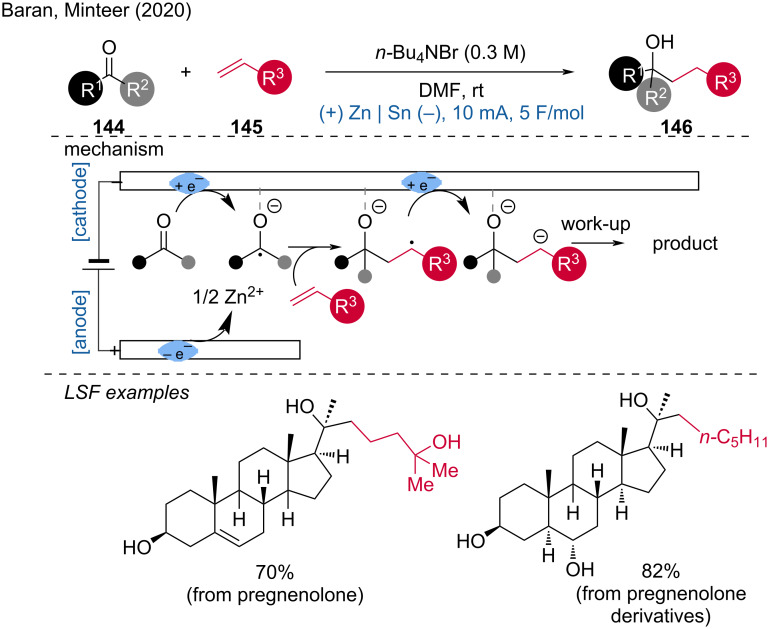
Electroreductive olefin–ketone coupling.

Another notable electroreduction by Baran, Minteer, and coworkers was the electroreductive coupling of unactivated aliphatic ketones with unactivated olefins [82]. This protocol highlights the importance of cathode choice, specifically noting that a tin (Sn) cathode facilitates the formation of ketyl radicals and their subsequent addition to the alkene counterpart. Cyclic voltammetry (CV) and squarewave voltammetry (SWV) results suggested an electrochemical-chemical-electrochemical-chemical (ECEC) mechanism, which begins with the formation of a ketyl radical, followed by its addition to the olefin. This is followed by a one-electron reduction of the resulting anion radical to a dianion, which, after protonation and subsequent work-up, yields the target product (Scheme 56). Recently, Kwak, Kim, and colleagues developed a similar electroreductive aza-Pinacol coupling involving unsaturated C–N and C–O bonds [83]. This further demonstrates the versatility and potential of electroreductive methods in achieving complex molecular transformations.

**Scheme 57:**
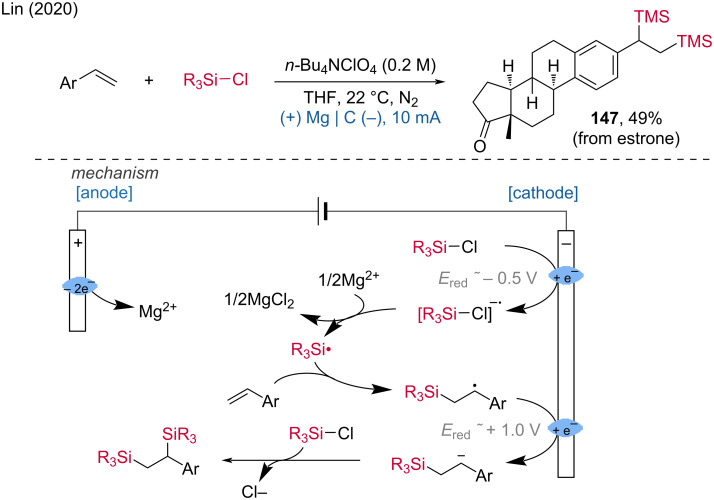
Electroreductive approach to radical silylation.

**2.1.2 Electroreduction of halides.** In 2020, Lin et al. developed an electrochemical di-silica-functionalization of alkenes using *n*-BuN_4_ClO_4_ as the electrolyte, magnesium as sacrificial anode, and graphite as cathode, to obtain an estrone derivative with 49% yield [84]. The proposed mechanism showed that the electrochemical process enables the highly efficient construction of vicinal C–Si bonds (Scheme 57). Furthermore, the same group reported the reductive cross-coupling of alkyl halides using electrochemistry [85]. These methods underscore the potential of electrochemical techniques in facilitating efficient and selective C–Si-bond formations.

**Scheme 58:**
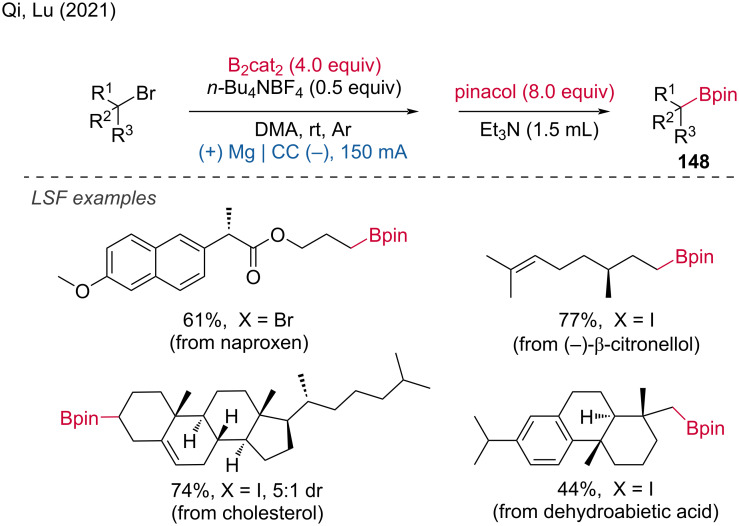
Electrochemical borylation of alkyl halides. CC = carbon close.

An electrochemical borylation method that demonstrated good stability and functional group tolerance was reported by Qi and Lu [86]. This method was successfully applied to the electrochemical borylation of natural products and pharmaceutical derivatives, including derivatives of naproxen, β-citronellol, dehydroabietic acid, and cholesterol, yielding the borylated products in 44–80% yields (Scheme 58). The substrate scope with more than 70 examples tested demonstrated not only its utility in the late-stage functionalization of biologically relevant compounds but also as general electrochemical borylation method.

**Scheme 59:**
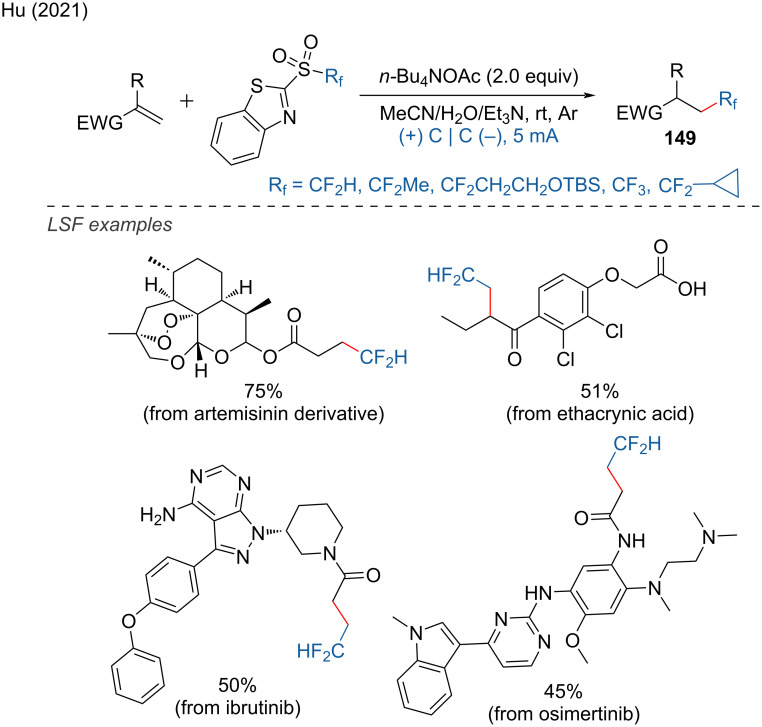
Radical fluoroalkylation of alkenes.

A radical fluoroalkylation can also be achieved through the electrochemical reduction of fluoroalkylsulfones, as demonstrated by Hu and coworkers [87]. The reaction took place in an undivided cell with graphite electrodes in acetonitrile at room temperature. This protocol is particularly suitable for the late-stage modification of biologically active molecules containing alkene functional groups. It was effectively used to obtain hydrodifluoromethylated analogs of an artemisinin derivative. Additionally, the protocol facilitated the hydrodifluoromethylation of ibrutinib, osimertinib, and etacrynic acid (Scheme 59).

**Scheme 60:**
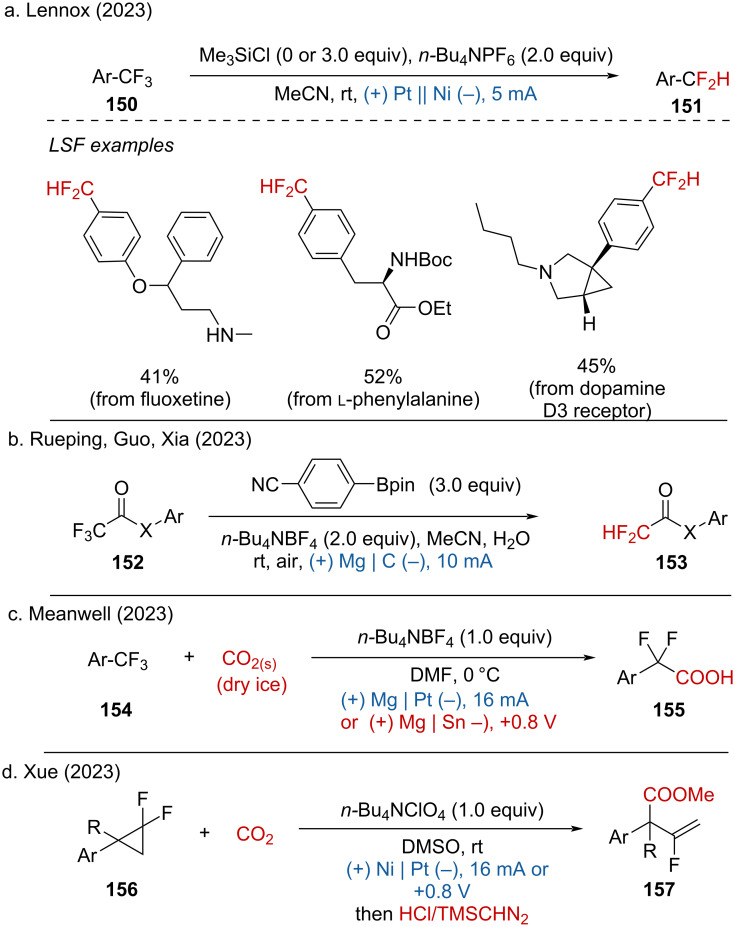
Electrochemical defluorinative hydrogenation/carboxylation.

The gem-difluoromethylene (–CF_2_R) moiety is an important structural component in drugs and agrochemicals. One of the most efficient and economical approaches to synthesize –CF_2_R groups is the selective defluorination of trifluoromethyl (–CF_3_) groups. In 2023, several electrochemical defluorinative hydrogenations and carboxylations via cathodic reduction were reported. Lennox and coworkers demonstrated the hydrodefluorination of aryl–CF_3_ to access aryl–CF_2_H via deep reduction on a nickel (Ni) cathode (Scheme 60a) [88]. In addition, Rueping, Guo and Xia disclosed the (deutero)hydrodefluorination of trifluoromethyl-substituted amides by utilizing an organoboron reagent to control the chemoselectivity (Scheme 60b) [89]. Furthermore, the Meanwell group reported the defluorinative carboxylation of (trifluoromethyl)(hetero)arenes, trifluoroacetates and -acetamides with the addition of CO_2_ (Scheme 60c) [90]. Besides, Xue and coworkers synthesized gem-difluorocyclopropanes via the defluorinative carboxylation of difluorocyclopropylarenes (Scheme 60d) [91].

**Scheme 61:**
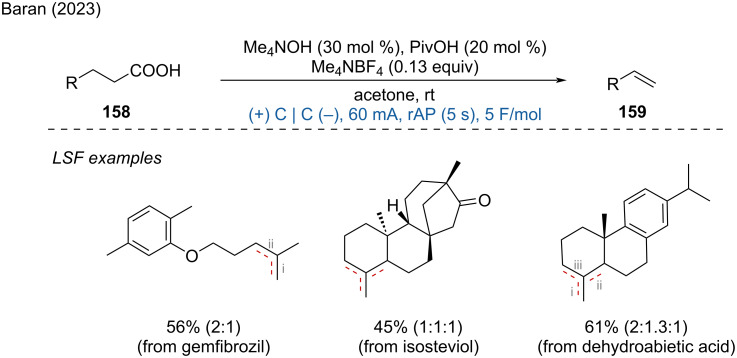
Electrochemical decarboxylative olefination.

**2.1.2 Electroreduction of carboxylic acids.** The Hofer–Moest reaction is a decarboxylative olefination process typically conducted under strongly oxidative conditions. In 2023, Baran and Kawamata reported a direct decarboxylative reduction of alkyl carboxylic acids to produce olefins under mild electrochemical condition [92]. This method allows for scalable synthesis, even at kilogram scales, and enables the LSF of natural products and drugs. Examples include the synthesis of valuable olefins from compounds such as gemfibrozil, isosteviol, and dehydroabietic acid (Scheme 61).

#### 2.2 Metal-catalyzed cathodic reduction

**Scheme 62:**
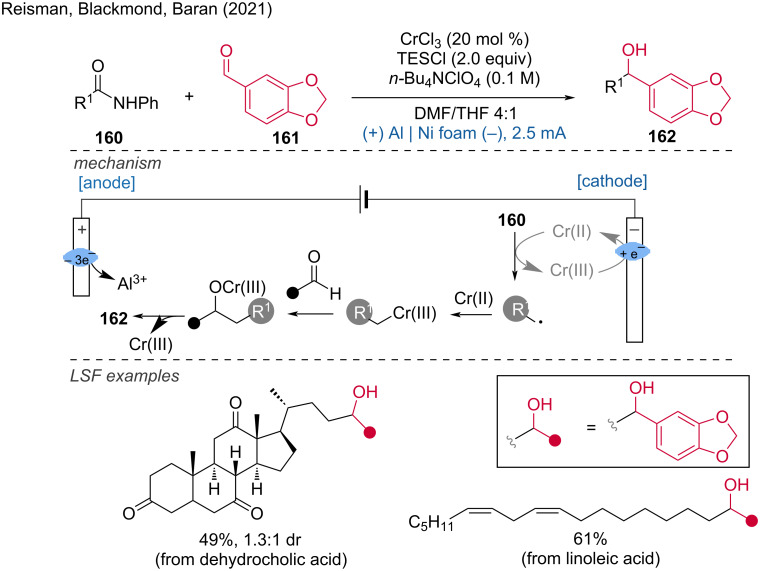
Electrochemical decarboxylative Nozaki–Hiyama–Kishi coupling.

Several metal-catalyzed (Cr [93], Co [94], Ni [95–96]) cathodic reduction-induced organic transformations have been developed in recent years. In 2021, Reisman, Blackmond, and Baran [93] disclosed the decarboxylative Nozaki–Hiyama–Kishi (NHK) coupling by combined electrochemical and Cr catalysis. The authors suggest that the success of the decarboxylating variant of this NHK reaction may be due to the rapid and selective reduction of Cr in contrast to chemical reducing agents. Also, the late stage functionalization of several natural substrates (dehydrocholic, linoleic, mycophenolic, and cholic acid) was demonstrated (Scheme 62).

**Scheme 63:**
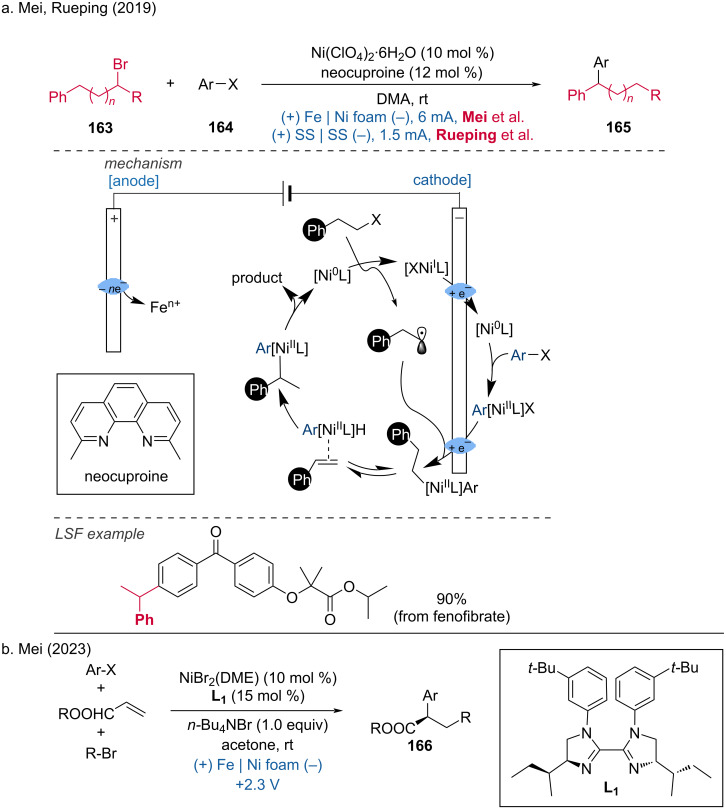
Nickel-catalyzed electrochemical reductive relay cross-coupling.

In 2019, Mei and Rueping reported a reductive relay cross-coupling between an aryl halide and an alkyl halide catalyzed by a Ni catalyst [97–98]. Fenofibrate readily participated in this reductive relay electrochemical coupling, producing the late-stage modified product in excellent yields. The reaction mechanism involves the reduction of Ni(II) to Ni(0), followed by the oxidative addition of an aryl bromide to Ni(0), forming an aryl–Ni(II) complex. After cathodic reduction, the aryl–Ni(I) complex is formed, which can react with the alkyl radical to form a Ni(II) species. Direct reductive elimination can lead to the formation of a linear byproduct, which can be converted to a more thermodynamically stable benzyl–Ni(II) intermediate through multiple β-hydride eliminations and reductions. After reductive elimination, the desired cross-coupled product and a Ni(0) species are formed. The methyl groups on the phenanthroline-derived ligand (neocuproine) are crucial for the reaction's success (Scheme 63a). In 2023, the Mei group extended this strategy to a three-component reaction by adding alkenes [99]. With nickel catalysis and a chiral ligand, the enantioselective difunctionalization of alkenes with electron-withdrawing groups (EWGs) was smoothly achieved (Scheme 63b). This extension highlights the versatility and potential of the nickel-catalyzed reductive relay cross-coupling in synthesizing complex molecules with high selectivity and efficiency.

**Scheme 64:**
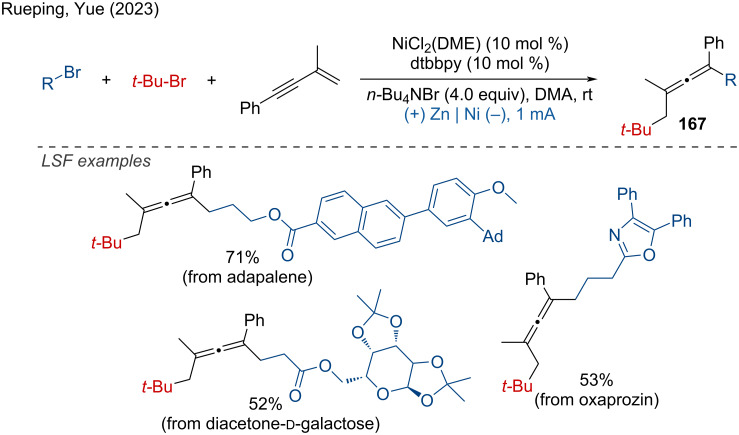
Electrochemical chemo- and regioselective difunctionalization of 1,3-enynes.

Recently, Rueping and Yue reported the electrochemical chemo- and regioselective difunctionalization of 1,3-enynes with two alkyl/aryl bromides [100]. With the readily available Ni(DME)Cl_2_ as catalyst, dtbbpy as ligand, the arylalkylation, dialkylation, and hydro(deutero)alkylation were achieved under mild reaction conditions. The LSF of natural products or drug motifs, such as adapalene, diacetone-ᴅ-galactose, and oxaprozin, proceeded smoothly with good chemo- and regioselectivity (Scheme 64). This method demonstrates the potential for selective modifications of complex molecules, enhancing their properties and expanding their applications in various fields, including pharmaceutical and materials science.

**Scheme 65:**
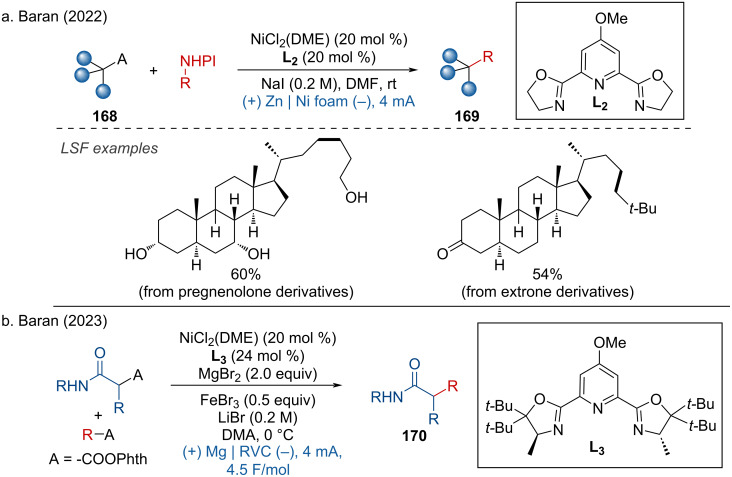
Electrocatalytic doubly decarboxylative cross­coupling.

In the past two years, a tremendous amount of work on the nickel-catalyzed cathodic reduction of halides [101–103], alkylpyridinium salts [104–105], and activated carboxylic esters [106] has been reported. In 2022, Baran and coworkers disclosed the electrocatalytic doubly decarboxylative cross­coupling via Ni(DME)Cl_2_·L2 complex as the catalyst. This method was demonstrated with pregnenolone and estrone derivatives (Scheme 65a) [96,107]. Additionally, the authors reported an asymmetric doubly decarboxylative C(sp^3^)–C(sp^3^) cross-coupling of two carboxylic *N-*hydroxyphthalimide (NHPI) esters, utilizing a PyBox-based chiral ligand. This approach yielded the amide products with good stereoselectivity (Scheme 65b) [108].

**Scheme 66:**
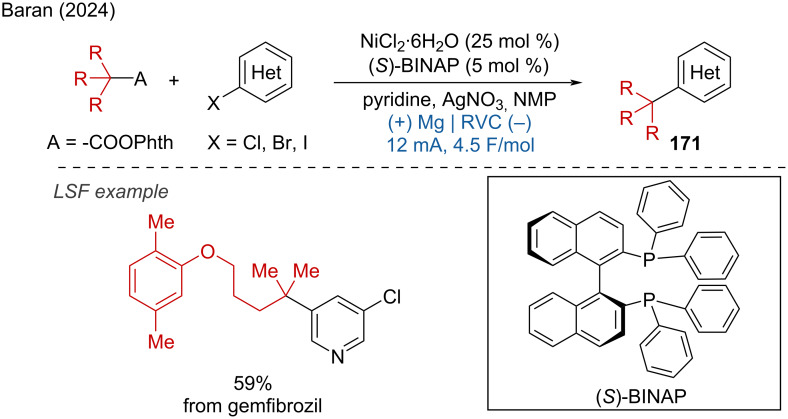
Electrocatalytic decarboxylative cross­coupling with aryl halides.

One year later, Baran and coworkers developed the C(sp^3^)–C(sp^2^) cross-coupling of redox-active esters and aryl halides to access quaternary carbon centers. Notably, gemfibrozil was successfully applied in a scale-up reaction (Scheme 66) [109].

**Scheme 67:**
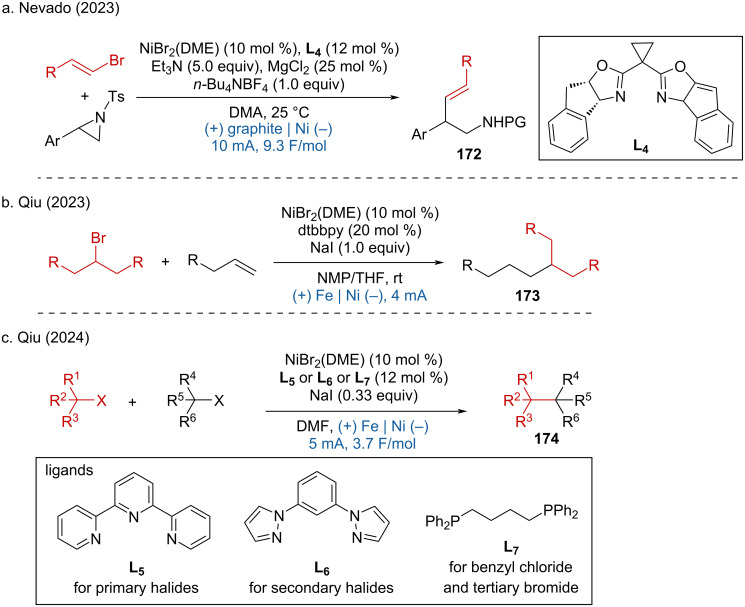
Nickel-catalyzed electrochemical reductive coupling of halides.

A nickel-catalyzed reductive coupling of alkenyl bromides with Ts-protected arylaziridine was reported by Nevado and coworkers achieving high enantioselectivity using a chiral bisoxazoline (L3) as the ligand (Scheme 67a) [110]. Additionally, Qiu and colleagues disclosed several nickel-catalyzed reductive cross-coupling reactions over the past two years. These include the C(sp^3^)–C(sp^3^) coupling of alkyl halides with alkyl alkenes (Scheme 67b) [111], the C(sp^3^)–C(sp^3^) coupling of *gem*-difluoroalkenes with alkyl halides [112], and the C(sp^3^)–C(sp^3^) cross-coupling of two unactivated alkyl halides, which were demonstrated for extended late-stage functionalization (eLSF) [113] (Scheme 67c).

**Scheme 68:**
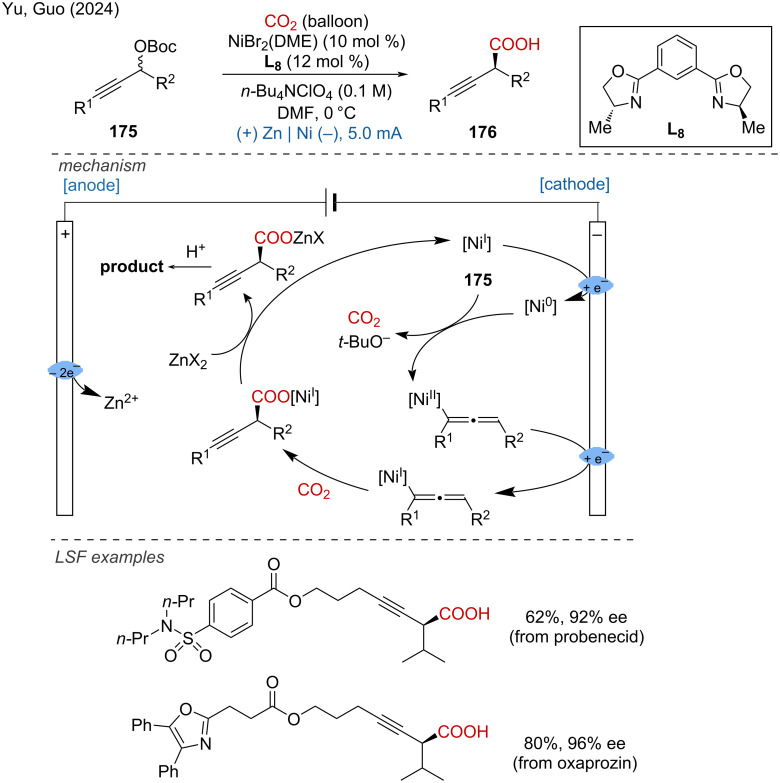
Nickel-electrocatalyzed enantioselective carboxylation with CO_2_.

CO_2_ is a sustainable C1 source for approaching carboxylic acids. In 2024, Yu, Guo and coworkers disclosed the enantioselective carboxylation of racemic propargylic carbonates with CO_2_ via a nickel-based electrocatalysis, for the synthesis of chiral propargylic carboxylic acids with high enantiomeric excess [114]. The efficient LSF of bioactive compounds such probenecid and oxaprozin proved the broad applicability of the method. Mechanistically, the nickel(II) precatalyst was initially reduced to the nickel(0) species, followed by oxidative addition with propargyl carbonate to obtain an allenyl–Ni(II) species. Subsequently, another cathodic reduction generates the allenyl–Ni(I) species, which sequentially undergoes migration insertion toward CO_2_, transmetalation with ZnX_2_, and deprotonation for approaching the desired propargylic carboxylic acid (Scheme 68).

#### 2.3 Cathodic photoelectrochemical reduction

**Scheme 69:**
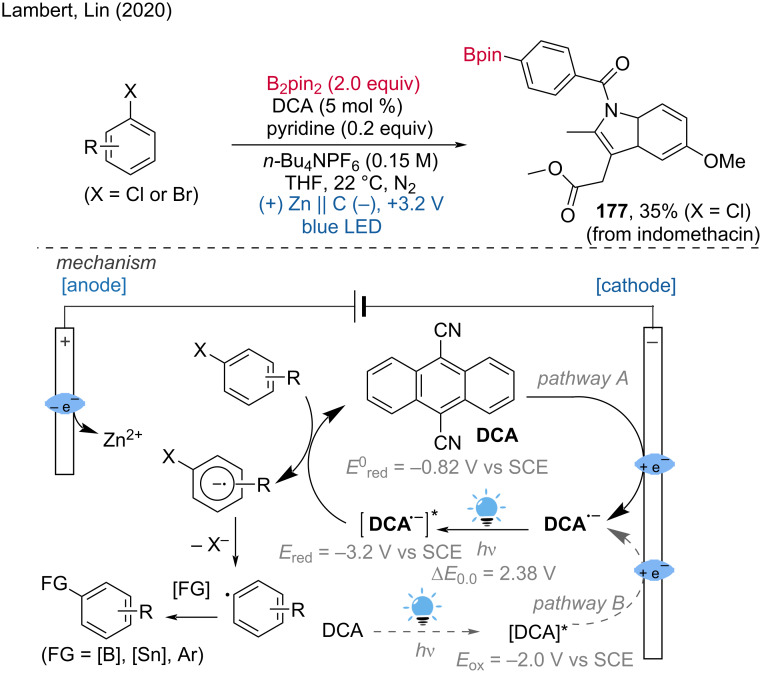
Reductive electrophotocatalysis for borylation.

Lin and Lambert et al. have been actively developing cathodic reduction methods that combine electrochemistry with photoexcitation of an electrogenerated radical anion or cation [115]. They demonstrated the electrochemical borylation of aryl bromides and chlorides using 9,10-dicyanoanthracene (DCA) electrolysis at a constant cell voltage under blue light irradiation. The mechanism involves the cathodic reduction of DCA (*E*_1/2_ = −0.82 V vs. SCE) on a porous carbon electrode, leading to the formation of a DCA radical anion. Subsequent photoexcitation of this radical anion produces a highly reducing radical anion in the excited state (*E*_red_ ≈ −3.2 V vs. SCE) with a calculated lifetime of 13.5 ns (note: the lifetime of DCA radical anion is controversial [116–118]), which is capable of reducing bromide and chloride substrates to facilitate the borylation reaction (Scheme 69).

**Scheme 70:**
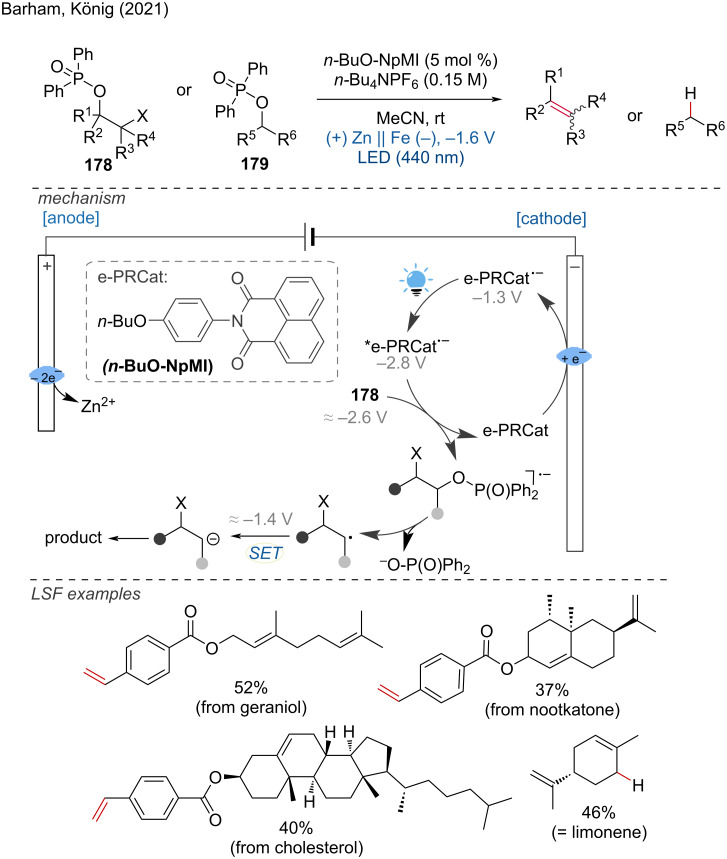
Electromediated photoredox catalysis for selective C(sp^3^)–O cleavages of phosphinated alcohols to carbanions.

Barham and König described an electrochemically mediated photoredox catalysis (ePRC) method for the reduction of phosphinates, achieving selective deoxygenative olefination and hydrogenation using *n*-BuO-NpMI as the catalyst [119]. This base-free approach demonstrated its utility by subjecting alkyl *p*-acetylbenzoate precursors of naturally occurring terpenes to extended late-stage functionalization (eLSF) with satisfactory yields. The authors found that the preassembly between the e-PRC radical anion and substrate is crucial for the chemoselective reduction of the C–O bond instead of C–Cl/Br bonds. Mechanistically, phosphinates of aliphatic alcohols (*E*^p^_red_ = −2.2 to −2.6 V vs. SCE) undergo ePRC reduction to produce carbanions. The SET reduction of the substrate forms an anion radical, which cleaves the C(sp³)–O bond and reduces the benzyl radical to an intermediate carbanion. This intermediate can undergo either olefination (when X = Cl, Br) or deoxygenation (when X = H), independent of hydrogen-atom transfer (HAT) or decarboxylation agents (Scheme 70). This method showcases the potential of integrating electrochemistry with photoredox catalysis to achieve efficient and selective transformations in organic synthesis, particularly for the functionalization of complex molecules.

### 3 LSF via paired electrolysis

Paired electrolysis is fascinating from an atom and energy efficiency perspective because it utilizes both electrodes for product formation, which offers significant advantages, including improved overall efficiency and the potential for more sustainable chemical processes. The further development of effective paired electrolysis methods will lead to innovative solutions in organic synthesis and industrial chemistry, promoting greener and more efficient reactions.

#### 3.1 Direct electrolysis of substrates

**Scheme 71:**
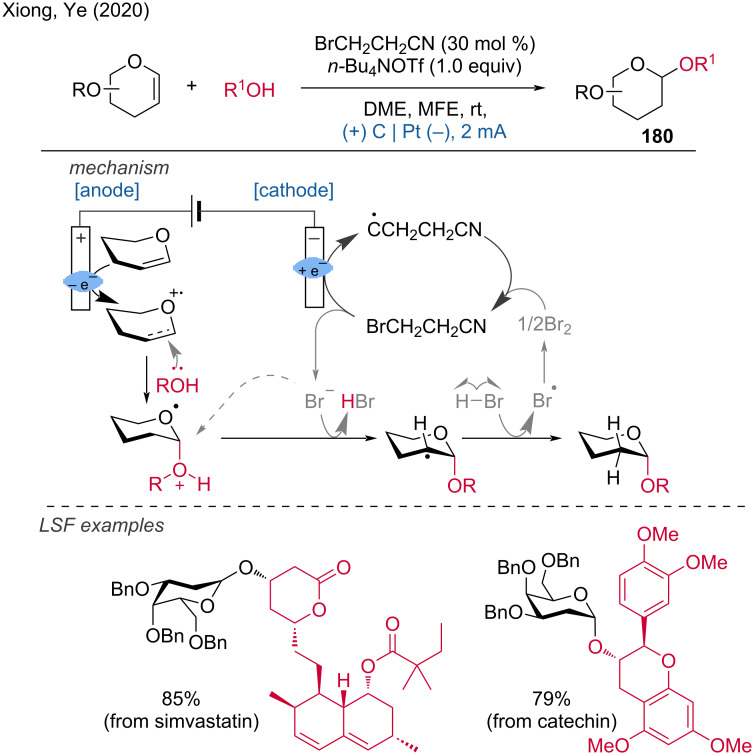
Stereoselective electro-2-deoxyglycosylation from glycals. MFE = methyl nonafluorobutyl ether.

In 2020, Xiong and Ye discovered a highly stereoselective electrochemical 2-deoxyglycosylation in an undivided cell using BrCH₂CH₂CN as an additive [120]. This process is applicable to the modification of a wide range of natural products and drugs, as demonstrated by the LSF of simvastatin, catechin, and estradiol benzoate. The mechanism proposed by the authors involves the cathodic reduction of BrCH₂CH₂CN to produce a propionitrile radical and Br^−^, which combines with H^+^ to form HBr. Concurrently, the anodic oxidation of the glycol substrate results in a radical cation intermediate. This intermediate undergoes a nucleophilic attack by an alcohol, followed by subsequent hydrogen abstraction, forming a glycosyl radical. This radical then abstracts hydrogen from HBr to produce the targeted 2-deoxyglycoside and a Br^•^ radical simultaneously (Scheme 71).

**Scheme 72:**
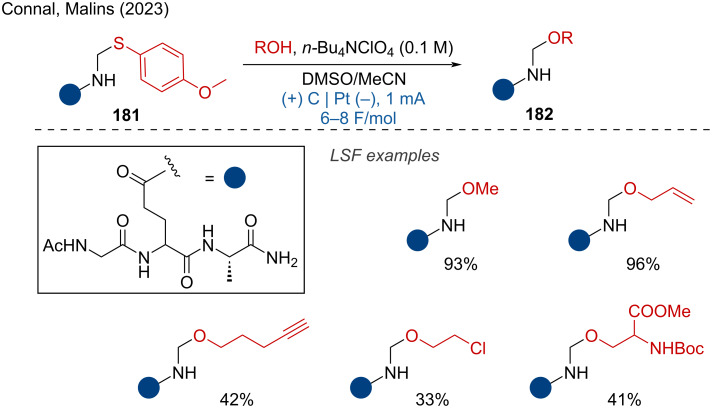
Electrochemical peptide modifications.

Recently, Connal, Malins, and coworker focused on tunable peptide modifications, developing an electrochemical side-chain orthogonal functionalization strategy for a diverse array of peptides [121]. This method successfully employed a wide range of alcohol nucleophiles, resulting in the formation of valuable *N*,*O*-acetals in moderate to good yields (Scheme 72).

**Scheme 73:**
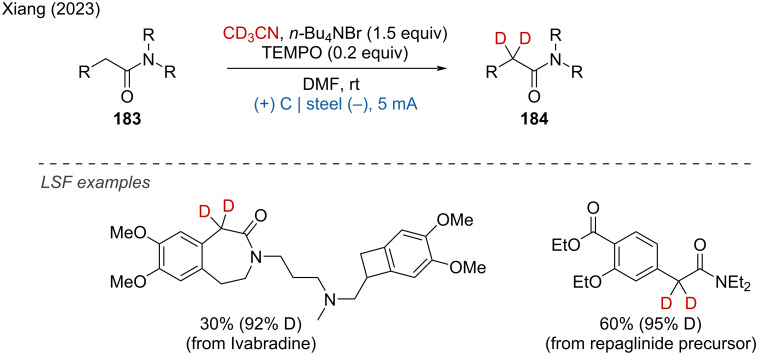
Electrochemical α-deuteration of amides.

The targeted deuteration of bioactive molecules and drugs is of great interest for improving their absorption, distribution, metabolism, and excretion (ADME) properties. Xiang and coworkers reported the H/D exchange of amides under mild electrochemical conditions using CD₃CN as an easily available deuterium source, resulting in α-deuterated amides with good yields and high deuterium incorporation [122]. The LSF of drug analogues demonstrated the method's good functional group tolerance and practicality (Scheme 73).

**Scheme 74:**
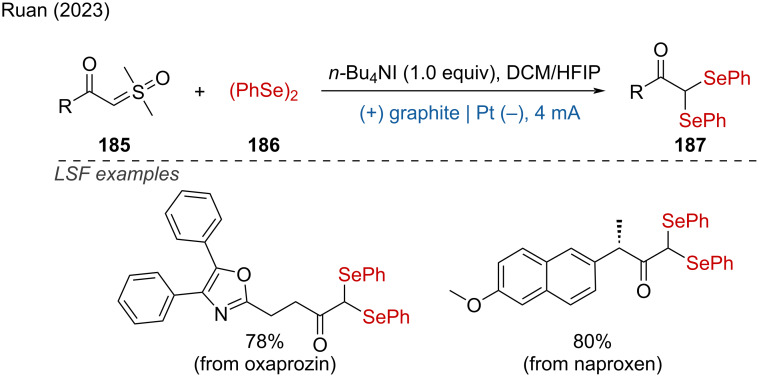
Electrochemical synthesis of *gem*-diselenides.

Furthermore, Ruan and coworkers reported the electrochemical coupling of α-keto sulfoxonium ylides with diselenides, affording *gem*-diselenides with high chemoselectivity and yields [123]. Mechanistic studies indicated that the use of *n*-Bu_4_NI as an electrolyte is a key factor for the efficient C–Se-bond formation. The reaction demonstrated ample scope, and the LSF of complex bioactive molecules such as oxaprozin and naproxen further confirmed the broad functional group tolerance of this method (Scheme 74).

**Scheme 75:**
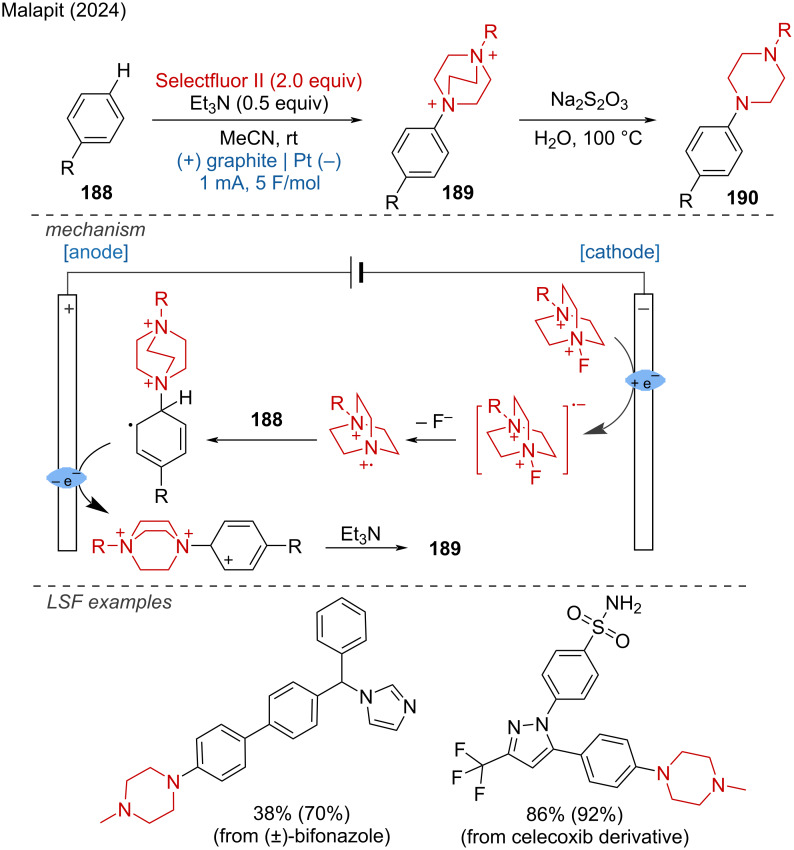
Site-selective electrochemical aromatic C–H amination.

Very recently, Malapit and coworkers developed an electrochemical aromatic C–H amination method using Selectfluor as the amination reagent [124]. This strategy achieved highly site-selective C–H aminations without the need for a directing group or metal catalyst. The mechanism involves the cathodic reduction of Selectfluor to generate highly electrophilic dicationic *N*-centered radicals, which efficiently react with aromatic C–H bonds. This is followed by anodic oxidation to generate the C–N-coupling product (Scheme 75). The method demonstrates excellent functional group tolerance, accommodating a broad range of aryl halides, carbonyls, sulfonamides, and heteroarenes.

**Scheme 76:**
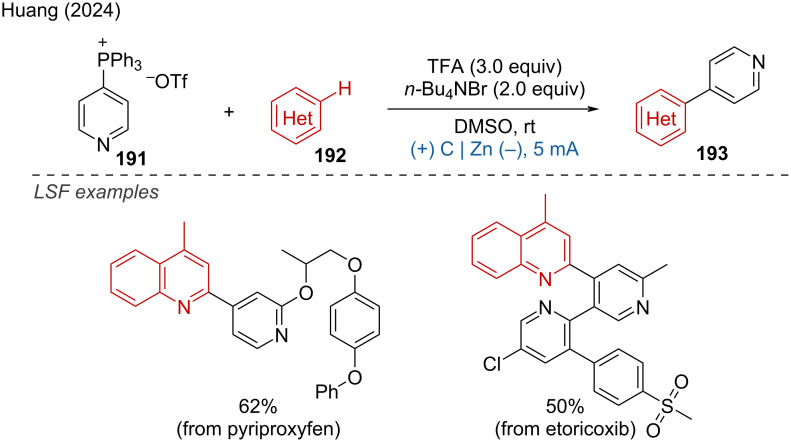
Electrochemical coupling of heteroarenes with heteroaryl phosphonium salts.

In the context of electrochemical biaryl synthesis, Huang and coworkers [125] reported the electrochemical coupling of *N*-heteroarenes with heteroaryl phosphonium salts [125]. This reaction features mild, redox-neutral electrolysis conditions that tolerate moisture and air and it is applicable to the LSF of derivatives of abiraterone, loratadine, etoricoxib, bisacodyl, and pyriproxyfen (Scheme 76).

#### 3.2 Metal-catalyzed paired electrolysis

**Scheme 77:**
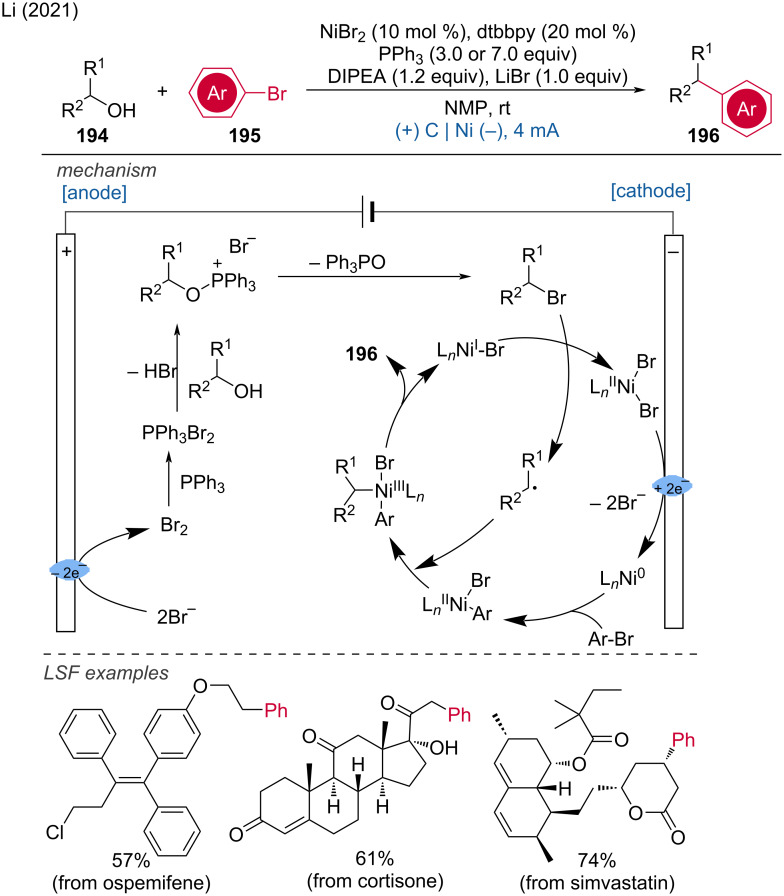
Redox-neutral strategy for the dehydroxyarylation reaction.

**3.2.1 Ni-catalyzed paired electrolysis. *Ni-catalyzed C–C coupling:*** A metal-catalyzed paired electrolysis has been achieved by Li and coworkers [126]. They developed a dehydroxylation reaction that directly activates alkyl alcohols using 3.0 or 7.0 equivalents of PPh_3_ as activation reagent for the cross-coupling with aryl bromides. This method successfully combines the Appel reaction at the anode and the reductive coupling reaction at the cathode. Detailed mechanistic studies indicate that the bromide anion in the electrolyte is first oxidized at the carbon anode of the electrolytic cell, losing two electrons to form elemental bromine. The Br_2_ then reacts with triphenylphosphine to form dibromotriphenylphosphine. The dibromotriphenylphosphine and the alcohol substrate undergo an Appel reaction to form an alkyl bromide. This alkyl bromide is subsequently reduced by Ni(I) to form alkyl radicals. Simultaneously, a Ni(II) salt is reduced at the nickel cathode to Ni(0), which undergoes oxidative addition to form Ni(II). The Ni(II) species then undergoes radical addition with the alkyl radical to form Ni(III). Finally, the Ni(III) species is reduced to afford the product and regenerate Ni(I), thus restarting the catalytic cycle (Scheme 77). The reaction was successfully applied to LSF and provided the products in good yields.

Another paired electrochemical cross-coupling reaction was reported by Liu and coworkers by applying aryl halides or β-bromostyrene electrophiles and benzyl trifluoroborates as nucleophiles [127]. To demonstrate the mildness of the conditions, the drug fenofibrate was successfully subjected to LSF, resulting in a series of coupled compounds with yields ranging from 41% to 86%. The proposed reaction mechanism suggests the initiation of the process by the cathodic reduction of the Ni(II) catalyst to Ni(I), which then undergoes oxidative insertion into the aryl halide substrate to form an Ar–Ni(III) complex.

**Scheme 78:**
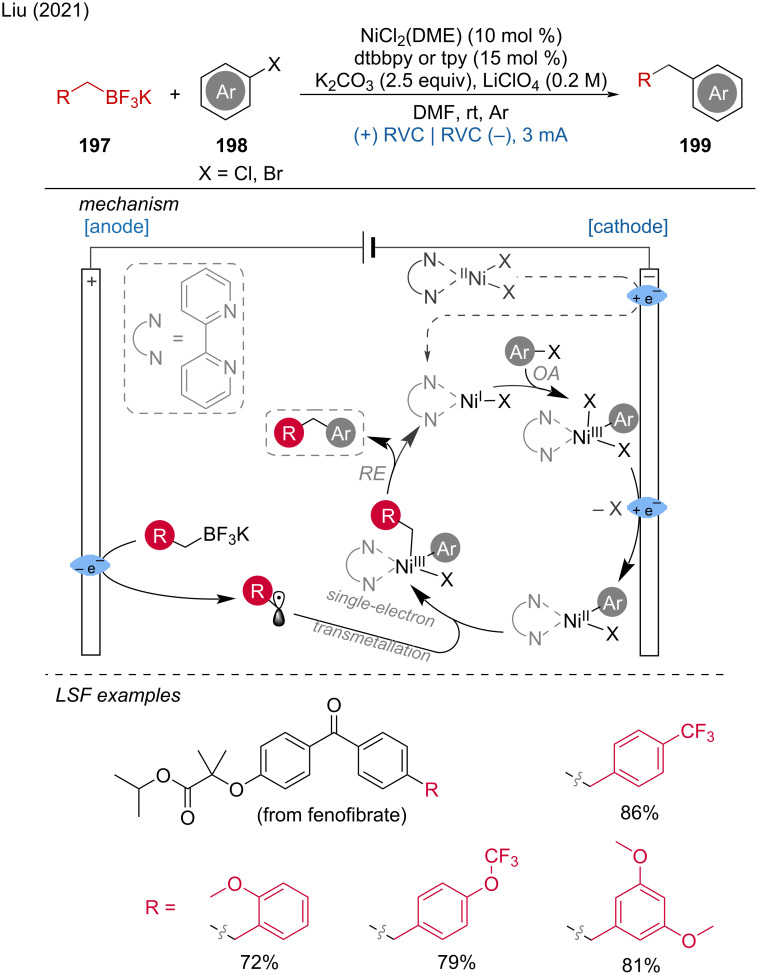
Nickel-catalyzed electrochemical C(sp^3^)–C(sp^2^) cross-coupling of benzyl trifluoroborate and halides.

This complex is subsequently electrochemically reduced to Ar–Ni(II), which interacts with the benzyl radical formed by the oxidative degradation of the potassium trifluoroborate substrate on the anode side. This results in the formation of a high-valent Ar–Ni(III)–Bn complex. The latter undergoes reductive elimination to produce the targeted cross-coupled product (Scheme 78).

**Scheme 79:**
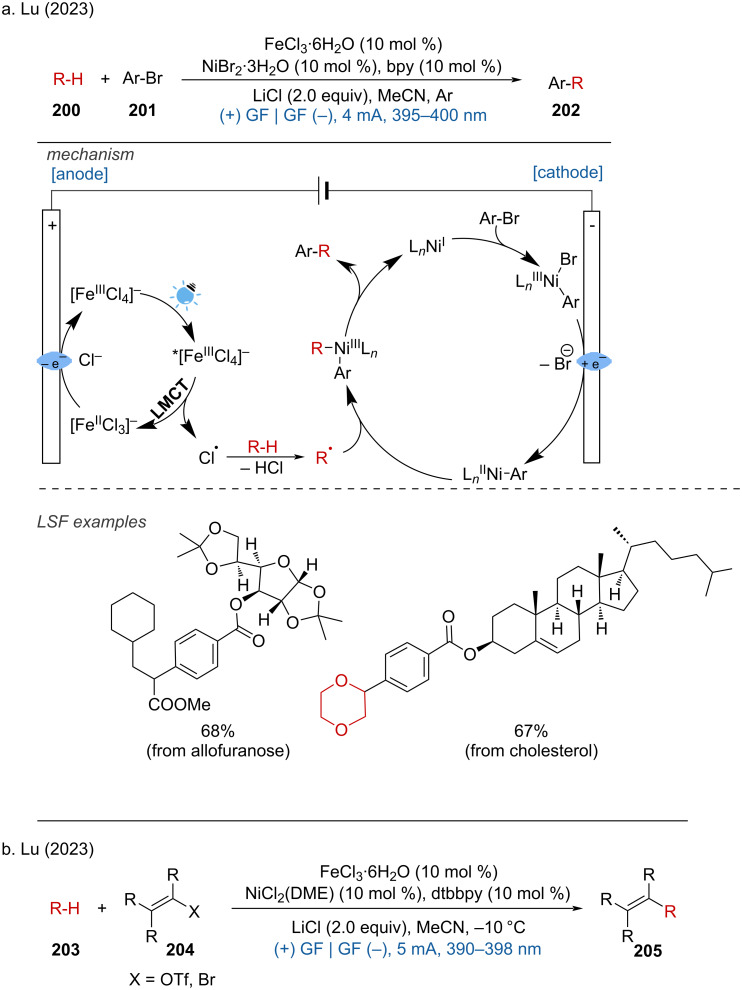
Paired electrocatalysis for C(sp^3^)–C(sp^2^) coupling.

A further paired electrocatalysis strategy for the C(sp^3^)–C(sp^2^) coupling of alkanes with aryl bromides [128] (Scheme 79a) and alkenyl triflate/bromides [129] (Scheme 79b), was recently disclosed by Lu and coworkers. In these reactions, FeCl_3_·6H₂O was employed as the anodic oxidative catalyst, while a nickel complex was used as the cathodic reductive catalyst. As illustrated in Scheme 79a, a chlorine radical (Cl^·^) is generated through ligand-to-metal charge transfer (LMCT) of the photo-irradiated iron catalyst, *[FeCl_4_]^−^. The Cl^·^ (with a bond dissociation energy (BDE) of HCl at 102 kcal/mol) is capable of abstracting a hydrogen atom from the aliphatic C–H bond (with BDEs of alkanes ranging from 96 to 101 kcal/mol), resulting in the formation of an alkyl radical (R^·^). Concurrently, the aryl bromide undergoes oxidative addition with Ni(I) to form an (aryl)Ni(III)Br species, which is then reduced at the cathode to (aryl)Ni(II). This (aryl)Ni(II) species subsequently undergoes radical addition with R^·^ and reductive elimination to yield the desired product. The robustness of this strategy is demonstrated by its broad substrate scope and its successful application to the LSF of natural products and pharmaceutical derivatives.

**Scheme 80:**
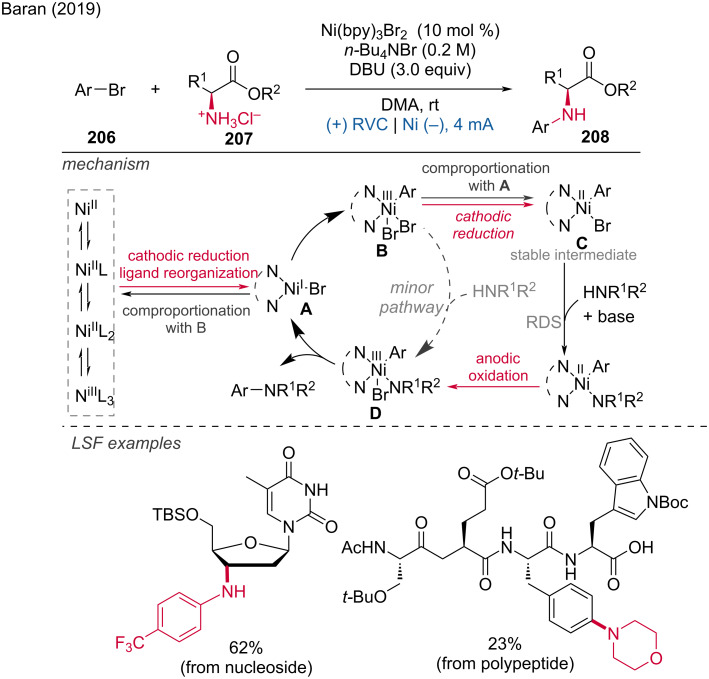
Redox-neutral strategy for amination of aryl bromides.

***Ni-catalyzed C–N coupling:*** C–N couplings via paired electrolysis were first reported by the Baran group [130]. Extensive mechanistic studies and a concomitant deeper mechanistic understanding led to further optimization in 2019, resulting in a protocol for redox-neutral nickel-catalyzed aminations of aryl bromides with far-reaching applicability for LSF [131]. This method allows the arylation of a wide range of amino acid esters and the amination of nucleoside analogs. By slightly adjusting the reaction conditions, the amination of polypeptides was also achievable. Unlike previous protocols, this method successfully enabled the arylation of a broad array of brominated heteroaryls. Additionally, not only amines but also other nitrogen nucleophiles (such as lactams and ammonia) and some oxygen-based nucleophiles were suitable coupling partners (Scheme 80).

**Scheme 81:**
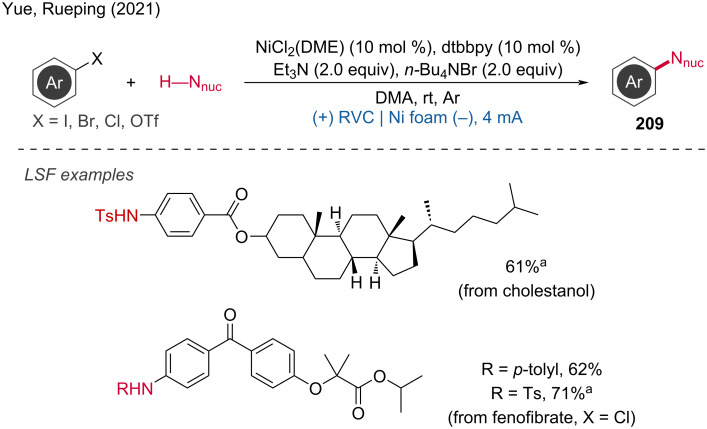
Redox-neutral cross-coupling of aryl halides with weak *N-*nucleophiles. ^a^Protocol with (+) RVC | RVC (–), 2-(*tert*-butyl)-1,1,3,3-tetramethylguanidine instead of Et_3_N.

A nickel-catalyzed cross-amination with weak nitrogen nucleophiles via matched paired electrolysis was reported by Yue and Rueping [132]. The developed approach is efficient for various C–N-bond formations (70 examples), including those involving electron-deficient anilines, using an RVC anode, nickel foam cathode, and triethylamine as the base. The protocol can also be adapted for the preparation of sulfonamides, carbamates, sulfoximines, and imines with slight modifications to the reaction setup (using (+)RVC/(−)RVC as electrodes and BTMG as a base). The LSF of pharmaceutical compounds has been applied to fenofibrate and cholestanol derivatives (Scheme 81).

**Scheme 82:**
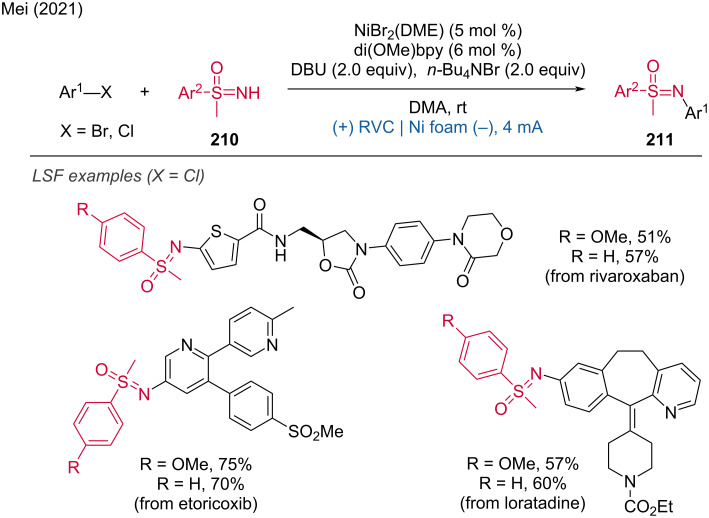
Nickel-catalyzed *N-*arylation of NH-sulfoximines with aryl halides.

Mei and coworkers [133] published a similar strategy for the *N-*arylation of NH-sulfoximines. The sulfoximidoyl derivatives are produced in good to excellent yields, demonstrating the mild conditions under which this method operates. Preliminary mechanistic studies indicate that the anodic oxidation of Ni(II) to Ni(III) is crucial for promoting the reductive elimination of the C–N bond from the formed Ni(III) species at room temperature (Scheme 82).

**Scheme 83:**
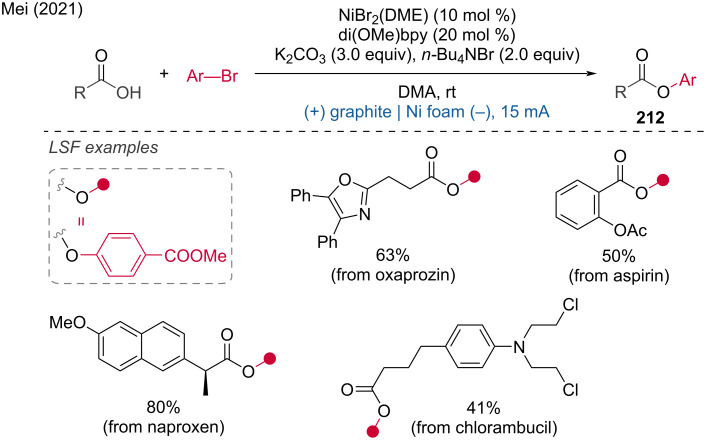
Esterification of carboxylic acids with aryl halides.

***Ni-catalyzed C–O coupling:*** The Mei group also reported an electrochemical method that enables the coupling of carboxylic acids with aryl halides via paired electrolysis [134]. This method was applied to the LSF of aspirin, ibuprofen, and naproxen, showcasing its applicability to various pharmaceutical compounds. Nitrogen- and oxygen-containing heterocycles were well tolerated and oxaprozin also gave a good yield of the desired product. Notably, chlorambucil underwent the reaction while alkyl chloride fragments remained unaffected (Scheme 83).

**Scheme 84:**
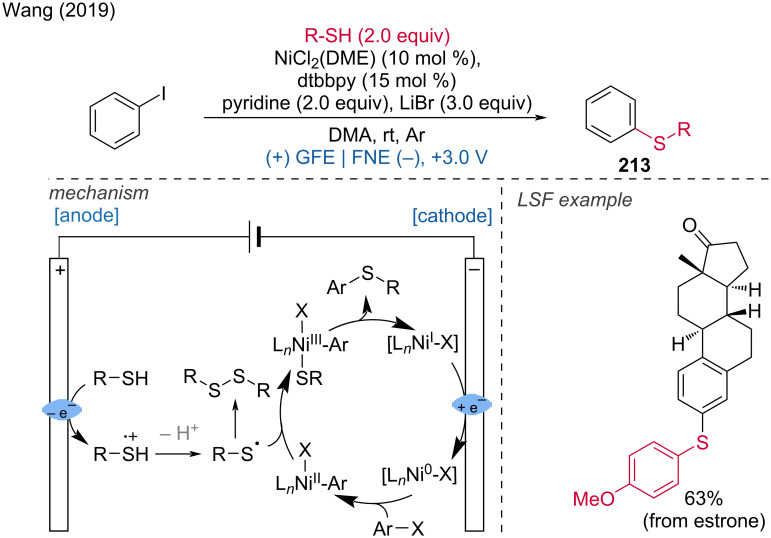
Electrochemically promoted nickel-catalyzed carbon–sulfur-bond formation. GFE = graphite felt electrode; FNE = foamed nickel electrode.

***Ni-catalyzed C–S coupling:*** Apart from C–N and C–O cross-couplings, Wang et al. reported a nickel-catalyzed Ullmann-type thiolation of aryl iodides under mild electrochemical conditions [135]. The reaction was conducted in an undivided cell with a graphene/nickel foam electrode, yielding aryl and alkyl sulfides in good yields. This electrochemical C–S cross-coupling was also applied for the LSF of thiol moieties in iodinated estrone (Scheme 84). In the reaction pathway, thiols undergo oxidation at the anode to form a thiol cation radical, which abstracts a proton from pyridine to generate a thiol radical or aryl disulfide. Concurrently, NiCl_2_·dtbbpy is reduced to Ni(0) at the cathode, which then undergoes oxidative addition with the aryl halide to form an Ar–Ni(II) complex. This complex interacts with the thiol radical to form a Ni(III) complex. Reductive elimination from the Ni(III) complex leads to the desired C–S cross-coupled product.

**Scheme 85:**
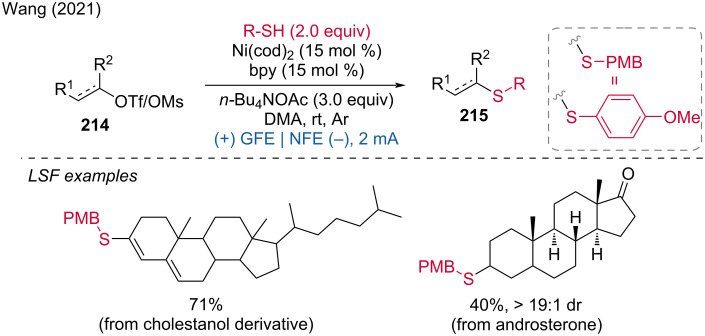
Electrochemical deoxygenative thiolation by Ni-catalysis. GFE = graphite felt electrode; NFE = nickel foam electrode.

Later the group reported the electrochemical deoxygenative thiolation of alcohols and ketone [136]. The reaction was carried out under argon atmosphere at a current of 2 mA with graphite felt as anode and nickel foam as the cathode. LSF for cholestanol derivatives and androsterone demonstrated good substrate tolerance of this protocol (Scheme 85).

**Scheme 86:**
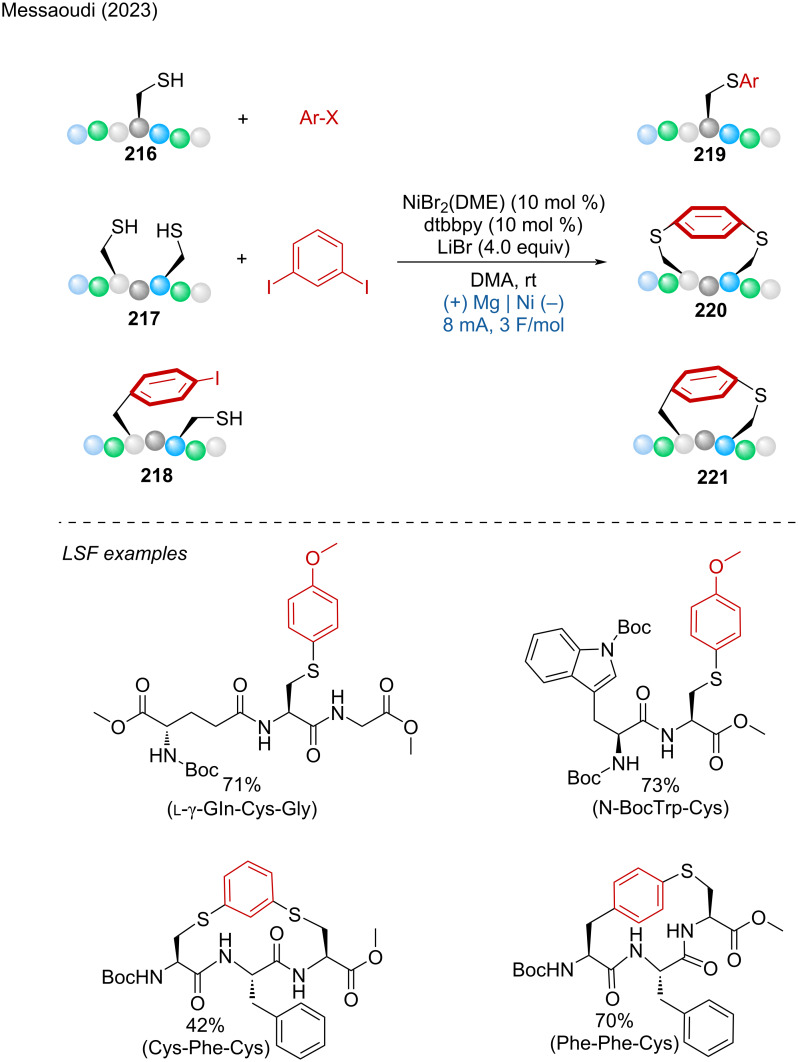
Electrochemical coupling of peptides with aryl halides.

In this context, Messaoudi and coworkers reported the electrochemical site-selective coupling of peptides (at the S–H bond) with aryl halides for the synthesis of *S*-arylated peptides [137]. The optimized reaction conditions include NiBr_2_·glyme and dtbbpy as the nickel catalyst complex, LiBr as the electrolyte, and an undivided cell with a magnesium anode and nickel foam cathode. This powerful method achieved both intramolecular and intermolecular couplings, providing valuable peptide conjugates and cyclic peptides in moderate to good yields (Scheme 86).

**Scheme 87:**
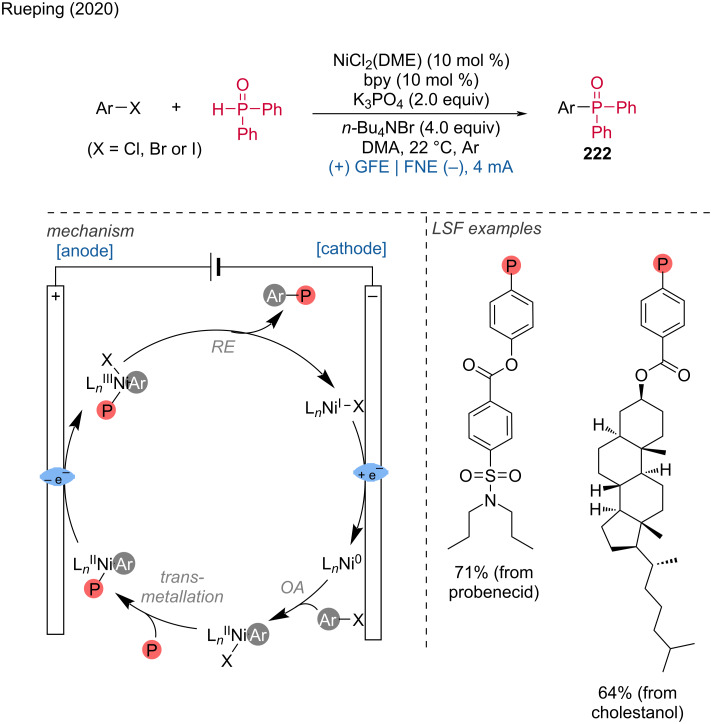
Paired electrolysis for the phosphorylation of aryl halides. GFE = graphite felt electrode, FNE = foamed nickel electrode.

***Ni-catalyzed C–P coupling:*** Organophosphorus compounds are valuable chemicals widely used in pharmaceuticals, agrochemicals, ligands, and materials. In 2020, Rueping and coworkers developed a method to form C–P and C–Se bonds by combining electrolysis and nickel catalysis [138]. The complex motifs such as probenecid and cholestanol were successfully converted into the corresponding phosphorylated derivatives with this method. Mechanistically, the Ni(0) catalyst firstly formed by cathodic reduction on a nickel foam cathode (FNE), then undergoes oxidative addition with aryl bromides, ligand exchange with the deprotonated diphenylphosphine oxide, and single electron oxidation by the graphite felt anode (GFE). Finally, the generated Ni(III) intermediate goes through a reductive elimination process to get the C–P cross-coupling product (Scheme 87). This method highlights the potential of combining paired electrolysis with nickel catalysis for the selective and efficient formation of C–P and C–Se bonds, providing a valuable tool for organic synthesis and the development of new materials and pharmaceuticals.

**Scheme 88:**
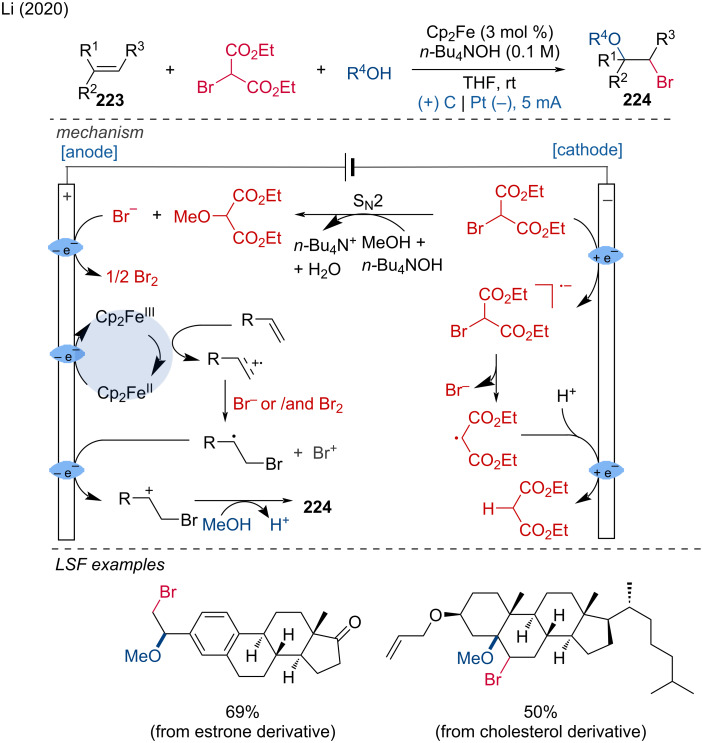
Redox-neutral alkoxyhalogenation of alkenes.

**3.2.2 Fe-induced paired electrolysis.** In 2020, Li and coworkers disclosed an approach for the electrochemical alkoxyhalogenation of alkenes via paired electrolysis in an undivided cell with graphite/platinum electrodes [139]. The authors sought alternative sources of halogens with higher oxidation potentials than alkenes, which could serve as the initial attacking nucleophiles. This strategy provides excellent selectivity and compatibility with a wide range of substrates while avoiding environmental halogen contamination due to the absence of halogen ion release during the cleavage of the carbon–halogen bond. Additionally, the use of Cp_2_Fe catalyst (3 mol %) in THF contributed to reducing the alkene oxidation potential from 1.68 V to 1.60 V, favoring the reaction. The authors successfully applied this strategy to important bioactive compounds such as adamantane, estrone, and ibuprofen derivatives (Scheme 88).

## Conclusion

The ever-expanding number of organic electrochemical reactions opens up new possibilities for medicinal chemists, providing them with novel opportunities to study structure–activity relationships (SARs) that were previously out of reach. Along with light-induced catalysis, synthetic organic electrochemistry is becoming a prominent method for functionalizing bioactive molecules due to its potential for mild reaction conditions, tunable constants and currents, and good functional group tolerance.

In this review, we comprehensively discuss recent advances in the electrochemical LSF of organic molecules, categorizing them into anodic oxidation, cathodic reduction, and paired electrolysis processes. Additionally, we cover photoelectrochemical methods, which combine photocatalysis and electrolysis to achieve previously inaccessible redox potentials, further expanding the scope of synthetic organic electrochemistry, and the pre-assembly between the radical ion photocatalyst and substrate could have great potential in selective functionalization for LSF [140–142]. As such this review aims to provide a systematic overview of these cutting-edge techniques, highlighting their applications and potential in fine-chemicals, pharmaceuticals, materials chemistry and beyond. We anticipate that further merging of electrochemistry with photocatalysis, biocatalysis, and enantioselective synthesis will result in new and improved reactivities, opening up new horizons in the field of organic chemistry and LSF.

## Data Availability

Data sharing is not applicable as no new data was generated or analyzed in this study.
